# Role of Central Arene Rotators and Ag^**+**^, I^**+**^, and PdCl_2_ Centers in Hexagonal Macrocycles as Molecular Rotors

**DOI:** 10.1021/acs.inorgchem.5c03802

**Published:** 2025-11-21

**Authors:** Nathaniel P. Litts, Ayden K. Lutes, Patrick A. Countryman, Eric A. Green, Alex I.P. Amore, William C. Lyons, Nathan J. France, Alexander W. Litts, Nathan P. Bowling, Joseph D. Scanlon

**Affiliations:** † Department of Chemistry, 2719 Wabash College, 301 W Wabash Ave, Crawfordsville, Indiana 47933, United States; ‡ Department of Chemistry and Biochemistry, 14756 University of Wisconsin-Stevens Point, 2101 Fourth Ave, Stevens Point, Wisconsin 54481, United States

## Abstract

Both trapezoidal and hexagonal macrocycles with central arene rotators have previously been reported to be synthesized and have the potential for use as molecular rotors. Hexagonal macrocycles with four different central arene rotators and three different coordinating centers (Ag^+^, PdCl_2_, I^+^) were examined using M06-2X/def2-SVP density functional theory and basis set. Minima were determined along with the rotational potential energy surface of the central arene to investigate the feasibility of the central arene rotating fully around the alkynyl axles and to explore what interactions with the surrounding stator might slow or block the movement of the rotator. Only the smallest central arene, **1**, a phenyl group, was found to be able to rotate fully through the macrocycle. In the gas phase, this rotation occurs with reorganization of the surrounding stator to minimize steric clashes between the endohedral hydrogens of the stator and the hydrogens of the rotator. For the benzothiadiazole rotator in **3** and the extended alkyne rotator of **4**, the sizes of the rotating units prevent full rotation through the macrocycle frame, and coordinating and aryl–aryl interactions with the hexagonal macrocycle, respectively, stabilize the rotator at 40°/140° angles with respect to the plane of the stator. Methoxy substituents on the rotator of **2** block full rotation and provide O···H–C attractions that stabilize the rotator near 40°/140° angles with respect to the plane of the stator. Systems utilizing a **PdCl**_**2**_ coordinating center generally exhibit the highest barriers to rotation, as movement of the Cl–Pd–Cl rotators in concert with the central arene comes with an energetic cost.

## Graphical abstract

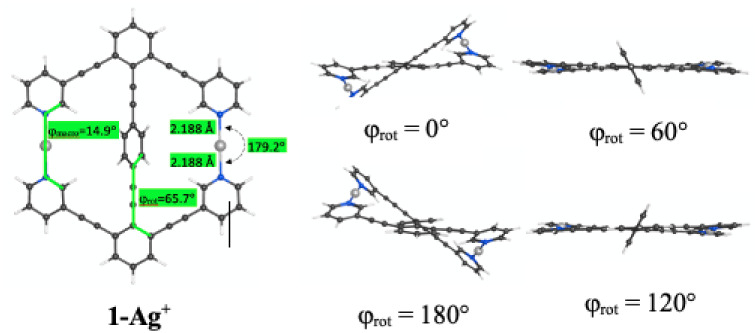

## Introduction

Rotation of a molecular component that is independent of or isolated from the rest of a molecule presents opportunities for the development of molecular turnstiles, switches, gears, and a variety of other molecular-scale rotor functions.^1^ Many types of linkages that provide low-energy barriers to rotation with a defined rotational axis have been investigated in molecular rotor assembly, including metal–ligand,^2^ hydrogen bond,^3^ and halogen bond^4^ linkers, to name a few. Alkynes are commonly employed as axles for rotors, as the barrier to rotation around the single bonds connecting alkynes to the rotating unit is—depending on the size of its substituents—quite small (often <1 kcal/mol).^5^ Because of this low barrier to rotation, arenes supported by alkyne axles are frequently chosen as rotators for molecular rotor design.^6^ Inspired by the seminal work of Moore,^7^ there is a general interest in installing rotating units of this type in the center of rigid macrocycles in order to isolate the rotator for turnstile^8^ or switching applications.^9^ These targets present synthetic challenges, however, that can be reduced if transition metal bridging between remote pyridyl ligands of an arylene ethynylene is used to provide macrocyclic arylene-ethynylene molecular rotors in which a 1,4-acetylene-functionalized arene serves as the rotator with alkynes functioning as the axles.^10^ Using this transition metal coordination strategy, a metal–organic hexagonal stator can be obtained in quantitative yield, providing a central rotator suspended in the center of a macrocyclic stator cavity by alkynyl axles (Figure 1).^11^

**Figure 1:**
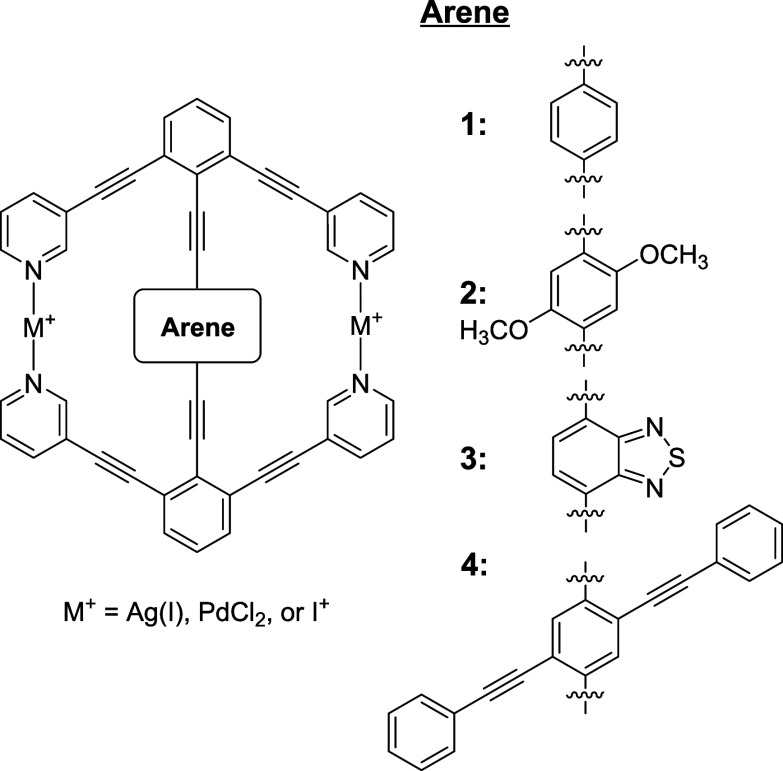
Four different central rotators (**1–4**) are investigated to explore rotational behavior in the cavity of the hexagonal macrocycle.

In prior studies of **1–4**, it has been established that macrocyclic rotors form in solution.^11^ Additionally, analysis in the solid state via X-ray crystallography (**1–3**) clearly illustrates a central arene surrounded by a hexagonal framework. Further characterization of **1** via ^13^C NMR reveals that a simple benzene unit moves and rotates independently of the hexagonal stator in the solid state. However, similar analysis of larger or substituted arenes is inconclusive, as intermolecular interactions necessitated by crystal packing—and not necessarily the size of the hexagonal cavity—prevent rotation of the central rotator. Consequently, there is still a question of what size/types of central arenes can achieve full rotation in our designed cavity rather than simple wobbling.

Different types of applications are achievable depending on the type of motion of a rotator. Fluorescent molecular rotors are commonly used in biological systems to monitor cellular conditions, such as viscosity.^12^ Rotators that cannot rotate freely due to either steric obstruction or some other barrier to rotation can serve as molecular switches, provided there is a suitable energy barrier between two distinct and accessible states and the potential to toggle between those states.^9^ Experimentally, rotation is often measured via variable-temperature solid-state (SS)^2^H NMR spin-echo measurements in comparison to line shape simulations. The movement of the rotator—which determines the line shape—is based on the balance of structural factors that destabilize or stabilize different conformers of the rotator. The objective of the current work is to identify the range of motion of each central arene, with special attention paid to the size limitations for full rotation and attractions or obstructions that may create an energy barrier for rotation between conformers.

## Computational Methods

All calculations were performed with M06-2X/def2-SVP using the Gaussian16 suite of electronic structure programs and visualized with WebMO.^13^ Calculations were performed using the ultrafine grid (int = ultrafine) and tight SCF convergence criteria (SCF = tight). Structures for **1-**, **2-**, **3-**, **4-Ag** ^**+**^, **PdCl**_**2**_, and **I** ^**+**^ were optimized and confirmed as minima by vibrational frequency calculations. Transition states were optimized and confirmed by vibrational frequency calculations for **1-** systems. To test basis set dependence, single-point energy calculations for minima and transition states were performed with the def2-TZVPP basis set.^14^ Minima and transition states were also optimized using ωB97x-d/def2-SVP.^15^ The def2-TZVPP and ωB97x-d calculation results are provided in the Supporting Information. For the lowest-energy conformer, potential energy surfaces for the rotation of the central arene were created by freezing the φ_rot_ dihedral every 10° while allowing the rest of the complex to fully optimize. Structures were visualized with WebMO, with carbon represented by black spheres, hydrogen represented by white spheres, nitrogen represented by blue spheres, chlorine represented by green spheres, palladium represented by navy blue spheres, silver represented by silver spheres, oxygen represented by red spheres, and iodine represented by purple .

## Results and Discussion

To explore the energy landscape for a solid-state rotor, it is sensible to lock the atoms of the stator in place and calculate relative energies as the dihedral angle of the rotator progresses from 0° to 360°. In solution, however—though the motions of the stator and rotator are somewhat independent—the stator is not constrained to a single conformation. Therefore, to mimic the likely behavior of the system in solution, the dihedral angle of the central arene in the gas phase was systematically changed, and the structure of the surrounding hexagon was minimized for each rotator conformation. The relative energies provided represent the lowest-energy conformations of the system at each designated dihedral angle for the central arene.

The choice of cations for this study reflects those that have been, or are most likely to be, used in the formation of the designed macrocycles. Reliable formation of macrocycles **1–4** requires metal centers that provide a linear coordination environment for the pyridine ligands. Common ions include Ag­(I) and Pd­(II).^10a^−c,^11,16^ By taking advantage of the *trans* arrangement of the chloride substituents on commercially available square planar PdCl_2_(CH_3_CN)_2_ or PdCl_2_(PhCN)_2_, displacement of the nitrile ligands provides a *trans*-spanning bipyridyl ligand around each metal center. In recent years, the use of I^+^ as a coordination site for supramolecular assembly has become common.^17^ Simple addition of I_2_ to the Ag­(I) salt results in precipitation of AgI and replacement of the Ag­(I) cation in the assembly with a similarly linear-coordinated I^+^. As the location of the conjugate anions that would be associated with Ag­(I) and I^+^ cations (often ^–^OTf, ^–^PF_6_, or ^–^BF_4_) would be impossible to reliably predict, they are ignored for the study.

### **1-Ag** ^**+**^, **1-PdCl**_**2**_, and **1-I** ^**+**^ Systems

For both **1-Ag** ^**+**^ and **1-I** ^**+**^, it is found that a simple benzene rotor will move with near-barrierless rotation in the cavity of a planar metal–organic macrocycle until it approaches coplanarity with the surrounding stator, at which point deformation of the macrocycle is energetically favorable in order to alleviate steric clash between the hydrogens of the central arene and the inner-facing hydrogens of the pyridine ligands. Though this distortion looks fairly dramatic (0° and 180° in Figure 2A), crossing through/full rotation of the central benzene is energetically feasible. Transition state structures were found for **1-Ag^+^** and **1-I^+^** with barrier heights of 5.2 and 4.2 kcal/mol, respectively (Figure 2B). The energetically identical 0° and 180° conformers indicate that the direction of the twist in the stator—at least computationally—depends on the approach direction from the rotation of the central rotator.

**Figure 2:**
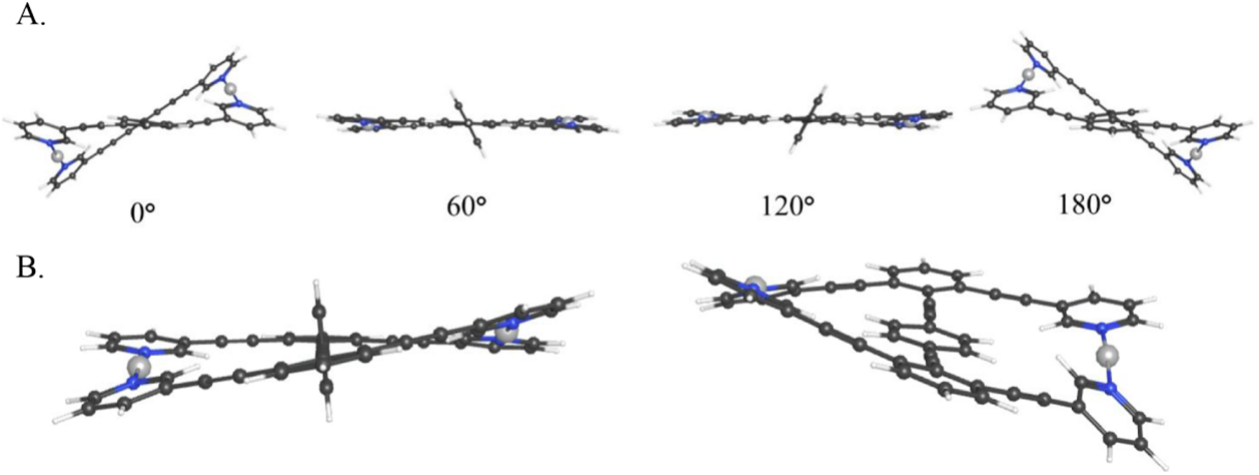
(A) Side view of **1-Ag** ^**+**^ for φ_rot_ = 0°, 60°, 120°, and 180°, showing twisting from a hexagonal stator as the central arene rotates. (B) Internal rotation transition state between symmetric minima and rotational transition state through the stator for **1-Ag** ^**+**^.

The internal rotational energy barrier for **1-Ag** ^**+**^ and **1-I** ^**+**^ can be seen in the graph in Figure 3, which illustrates a small (∼0.1 kcal/mol) rotational barrier between the two symmetric minima—reflective of the decrease in conjugation that occurs with a perpendicular arrangement of the rotor with the unsaturated stator—along with showing the increase in energy at 0° and 180° that reflects the distortion of the stator to minimize steric clashing with the hydrogens of the rotator. The N–I^+^–N angle in **1-I** ^**+**^ was found to have the identical value of 179.5° as in Lindblad et al.’s trapezoid analogue to **1-I** ^**+**^, showing the halogen bond is unstrained and there is no interaction between the benzene and I^+^.^10a^

**Figure 3:**
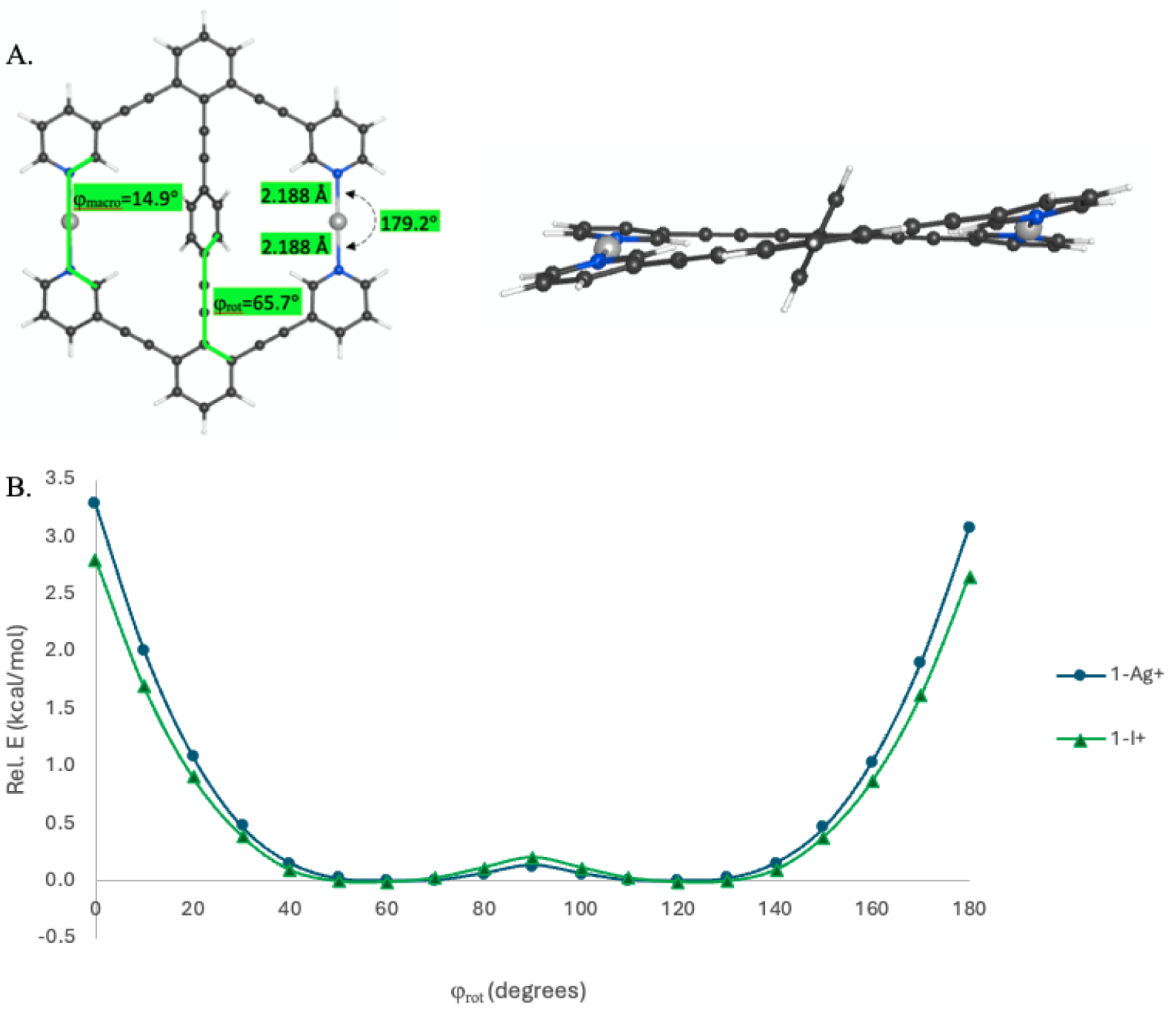
(A) Front and side views of the lowest energy conformer of **1-Ag** ^**+**^. (B) Rotation potential energy surface of the central arene with respect to the benzene units of the macrocycle, measured by the φ_rot_ dihedral for **1-Ag** ^**+**^ and **1-I** ^**+**^.

The Cl–Pd–Cl units in **1-PdCl**_**2**_ complicate the rotation of the central benzene in ways that are impossible to accurately convey without treating the Cl–Pd–Cl units themselves as independent rotators. This can be seen from the three structural depictions in Figure 4 where the Cl–Pd–Cl units are allowed to rotate to energetically minimized positions relative to the central benzene. **1-PdCl**_**2**_ has three distinct minima due to the interaction of the central phenyl arene with the chloro ligands of the palladium. The lowest energy conformer has the plane of the phenyl group almost perpendicular to the two Cl–Pd–Cl planes, while the conformer with one Cl–Pd–Cl perpendicular is 2.7 kcal/mol higher, and the conformer with the phenyl and both Cl–Pd–Cl groups parallel is 5.2 kcal/mol higher.

**Figure 4:**
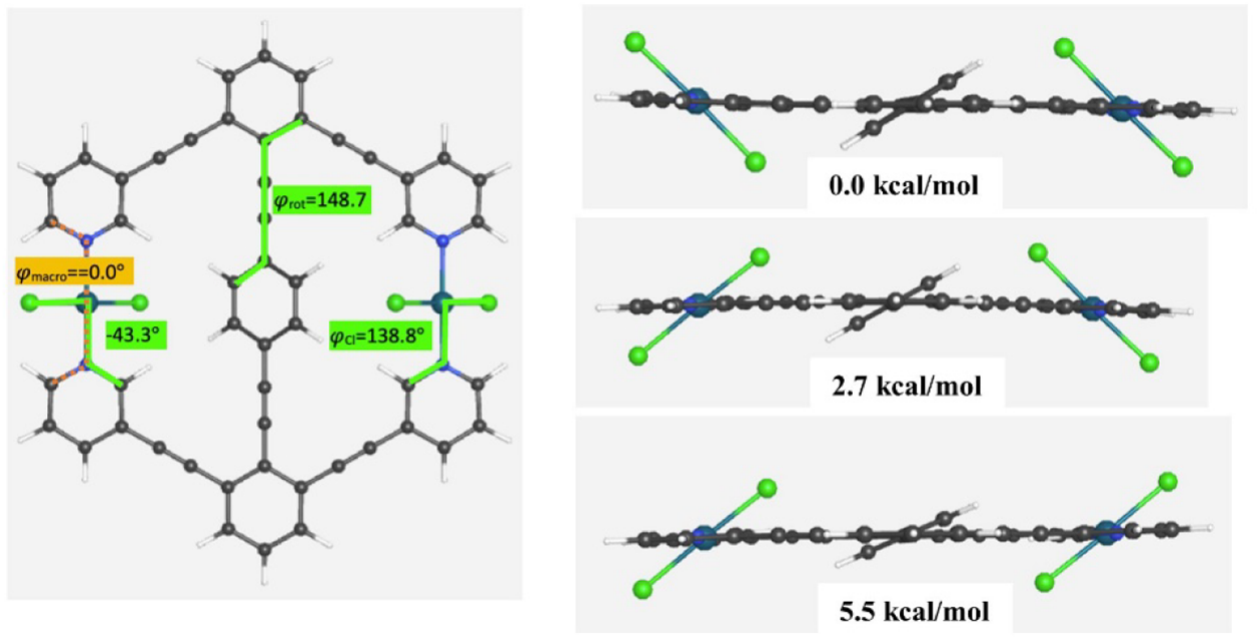
Top view of the lowest energy conformer of **1-PdCl**_**2**_, along with side views of the other two conformers of **1-PdCl**_**2**_ with relative energies listed.

As the movement of each rotator influences the position of the other two, there is potential for molecular gearing that is worth exploring. To this end, two different sets of calculations were performed. As with the Ag^+^ and I^+^ systems, the potential energy surface for the lowest energy conformer was found when freezing the φ_rot_ dihedral every 10° while allowing the rest of the complex to fully optimize, including the Cl–Pd–Cl rotators (Figure 5A). For the second, a new dihedral for the Cl–Pd–Cl units, φ_Cl_, was frozen at values from 0 to 180° while allowing the benzene rotator to change position (Figure 5B). With the Cl–Pd–Cl rotators, there is an energetic minimum when the Cl–Pd–Cl rotator is 40° out of plane from the attached pyridines, likely due to hydrogen bonding between the Cl and the polarized hydrogens of the pyridines within the coordination sphere. The minimum for the central rotator, therefore, is expected to reside where there are the most supportive attractions between the rotator and these tilted Cl–Pd–Cl units while minimizing steric or nonsupportive interactions. Cl···H-rotator attractions likely explain the minimum (Figure 4, top) observed for 1-PdCl_2_.

**Figure 5:**
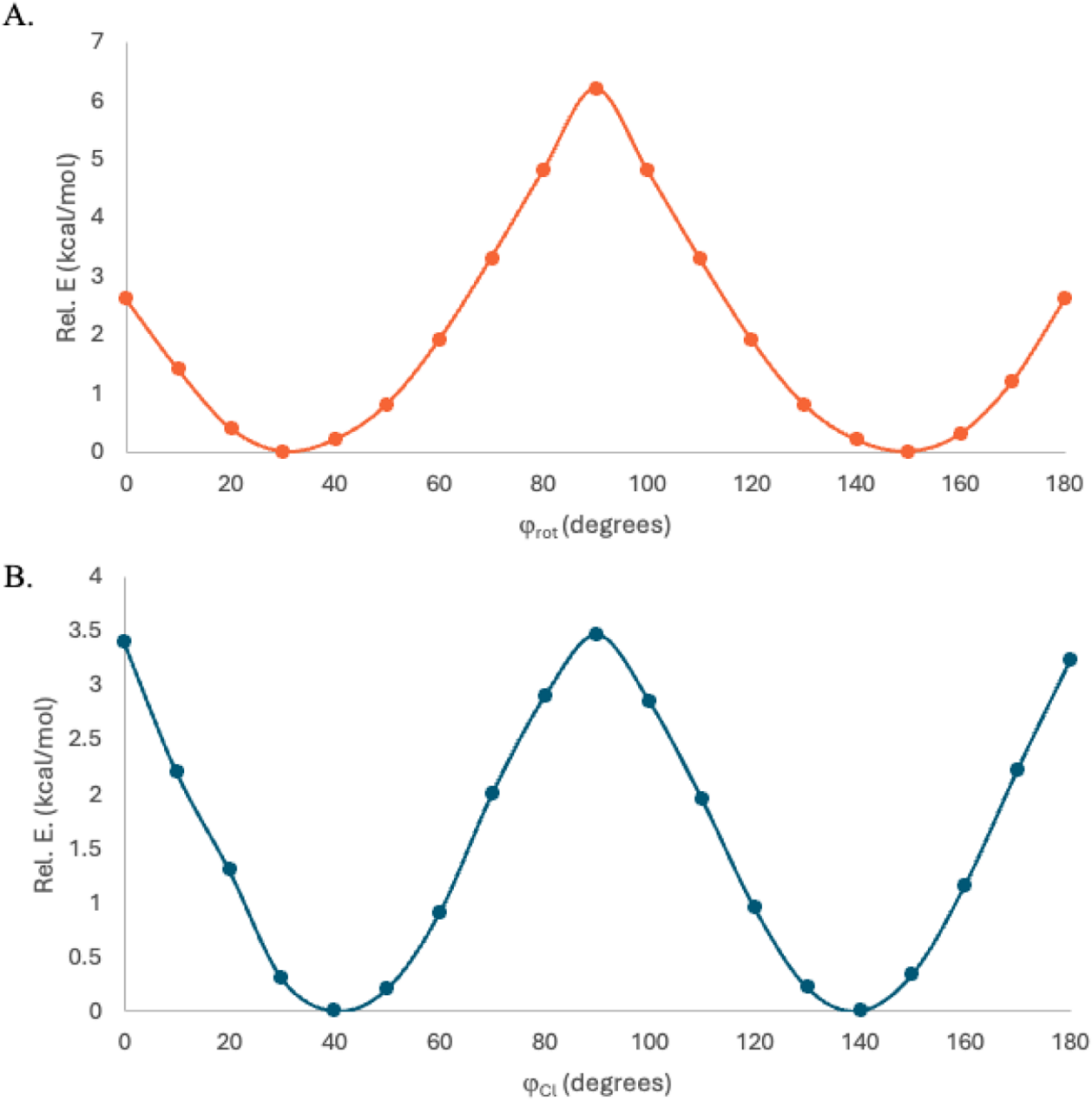
Rotation potential energy surface of: A. The central arene with respect to the benzene units of the macrocycle, measured by the φ_rot_ dihedral for **1-PdCl**_**2**_. B. The Cl–Pd–Cl units with respect to the pyridine units of the macrocycle, measured by φ_Cl_.

As φ_rot_ dihedral was frozen at different values from 0 to 90°, the relative position of the central benzene and Cl–Pd–Cl changes little; instead, the macrocycle twists (Figure 6A). After 90°, the Cl–Pd–Cl rotators flip to adopt the lowest energy orientation, as shown in Figure 4. Similarly, as φ_Cl_ is held at different values, the macrocycle twists allow the central benzene and Cl–Pd–Cl rotators to stay in the same conformation until 90° is reached (Figure 6B). Then, the central benzene and the other Cl–Pd–Cl rotor flip to the lowest energy orientation, where the central benzene is perpendicular to the Cl–Pd–Cl rotators. With the flexibility of the macrocycle, it appears that no gearing is occurring in solution.

**Figure 6:**
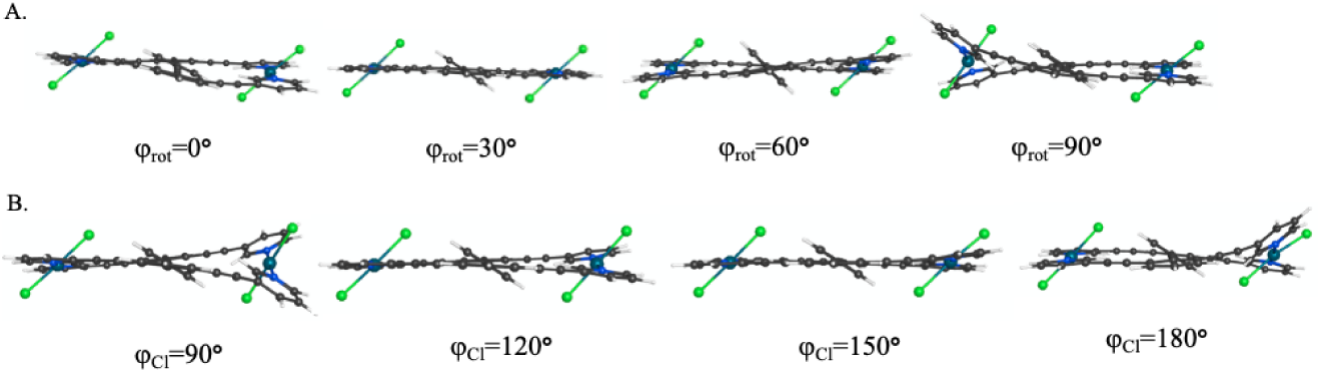
(A) Side view of **1-PdCl**_**2**_ for φ_rot_ = 0°, 30°, 60°, and 90°, showing twisting from the hexagonal stator as the central arene rotates. (B) Side view of **1-PdCl**_**2**_ for φ_Cl_ = 90°, 120°, 150°, and 180°, showing twisting from the hexagonal stator as the Cl–Pd–Cl axis rotates.

### **2-Ag** ^**+**^, **2-PdCl**_**2**_, and **2-I** ^**+**^ Systems

Both **2-Ag** ^**+**^ and **2-I** ^**+**^ (shown in Figure 7) have the lowest-energy conformer with both methyl groups of the methoxy substituents out of the plane of the central arene, along with higher-energy conformers where one methyl is out of plane and one is in plane, or both are in plane. The lowest-energy conformer also allows interaction between the oxygen of the methoxy group and the highly polarized pyridine hydrogen, which are 2.217 Å apart. The **2-PdCl**_**2**_ has a parallel arrangement of rotators with the methyl groups in the plane to minimize steric interaction while maximizing O···H-pyridine attractions. As in the case of both **2-Ag** ^**+**^ and **2-I** ^**+**^, other conformers were found with one or both methyl groups of the methoxy groups out of the plane.

**Figure 7:**
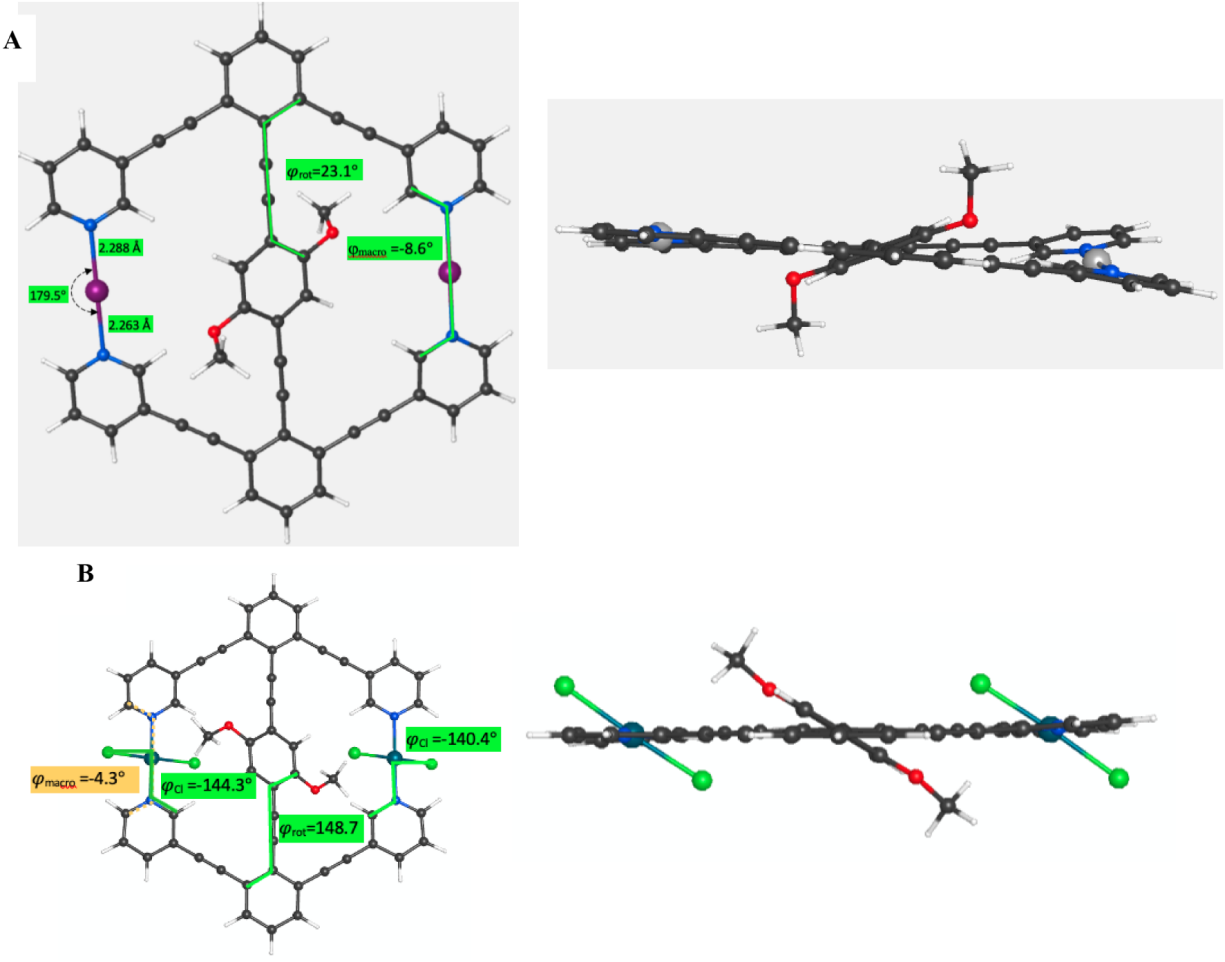
(A) Side and top views of the lowest energy conformer of **2-I** ^**+**^ with a φ_rot_ dihedral of 23.1°, showing the methyl groups out of the plane of the central arene. (B) Side and top views of the lowest energy conformer of **2-PdCl**_**2**_ with a φ_rot_ dihedral of 148.7°, showing the methyl groups in the plane of the central arene.

Scanning the φ_rot_ dihedral for the **2-** compounds results in a different rotational potential energy surface than the **1-** compounds (Figure 8). Initially, the energy decreases until the first minimum is found for the position of the central arene. Larger barriers to internal rotation were found at 90° for **2-Ag** ^**+**^ and **2-I** ^**+**^, measuring 4.7 and 7.8 kcal/mol, respectively, compared to the **1-Ag** ^**+**^ and **1-I** ^**+**^ systems. This is due to the reduced attractions between the oxygen of the methoxy group and the highly polarized pyridine H. After the central arene rotates past 90° for **2-Ag** ^**+**^ and **2-I** ^**+**^, steric clash between the methoxy group and the hexagonal macrocycle stator causes the methoxy group to rotate out of plane in the opposite direction. For **2-PdCl**_**2**_, the Cl–Pd–Cl groups rotate to face the opposite direction after φ_rot_ = 90° to avoid steric clash with the methoxy groups. We were not able to locate a transition state for the central arene rotating through the plane of the macrocycle. Attempting to extend the rotational energy surface past 180° resulted in larger twisting of the macrocycle (φ_rot_) and increasing energy. To get an upper bound for the barrier to the central arene passing through the stator, we performed calculations changing φ_rot_ dihedral without allowing the rest of the molecule to relax (Figure 8B). This resulted in an extremely large barrier of 87.6 kcal/mol, suggesting that the presence of methoxy groups on **2** makes full rotation of the central arene not energetically feasible. The barrier is not located at φ_rot_ = 0° due to slight twisting of the macrocycle. This inability of the methoxy-functionalized rotator to pass through the metal–organic macrocycle is consistent with the findings of Varnali^8^ for the fully covalent macrocycle of the same size.

**Figure 8:**
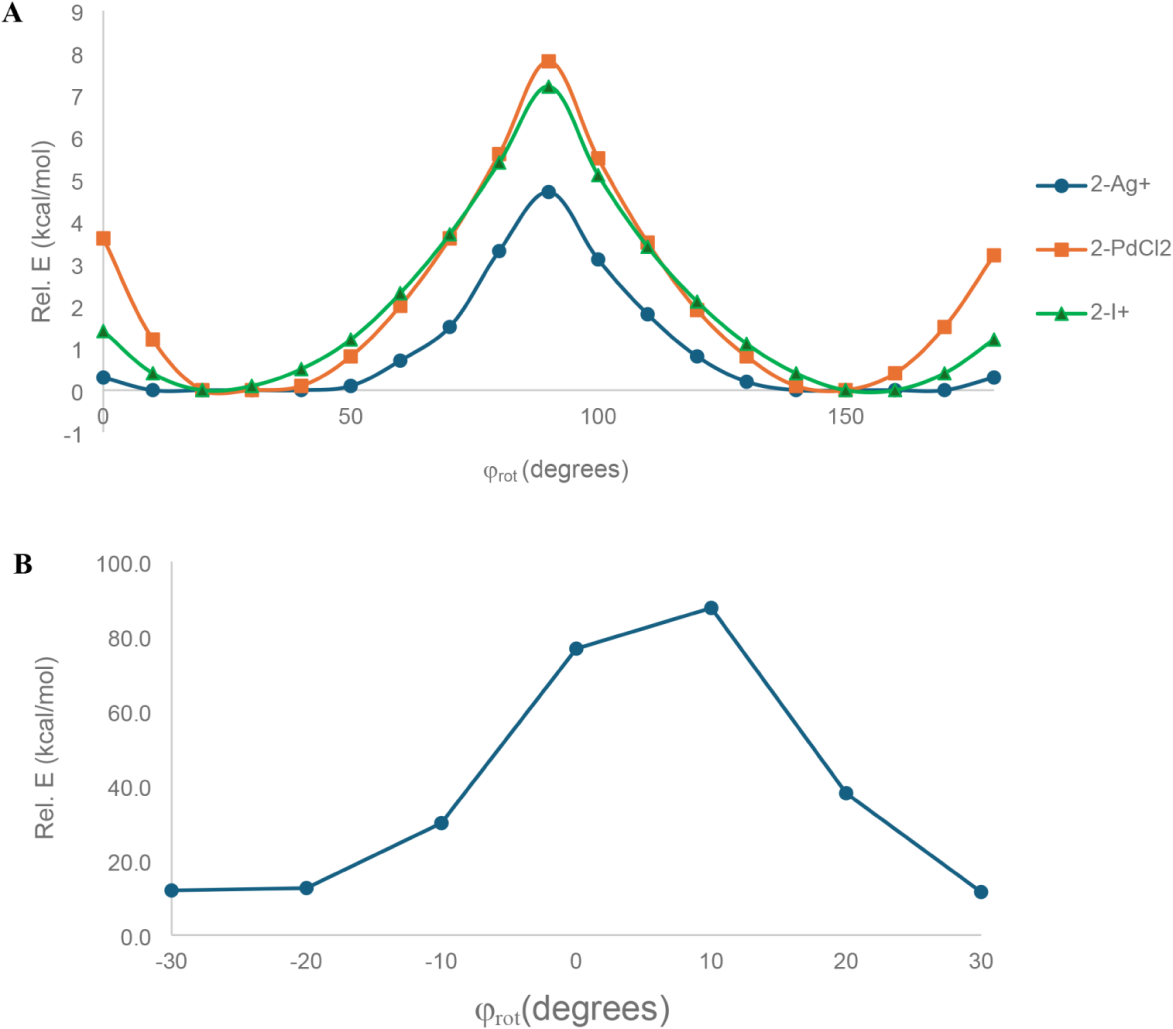
(A) Rotation potential energy surface of the central arene with respect to the benzene units of the macrocycle, measured by the φ_rot_ dihedral for **2-Ag** ^**+**^, **2-PdCl**_**2**_, and **2-I** ^**+**^. (B) Nonoptimized rotation potential energy surface of the central arene through the plane of the hexagonal macrocycle stator for **2-Ag** ^**+**^.

### **3-Ag** ^**+**^, **3-PdCl**_**2**_, and **3-I** ^***+***^ Systems

Both the **3-Ag** ^**+**^ and **3-I** ^**+**^ were found to have two symmetric minima, as shown in Figure 9. The **3-Ag** ^**+**^ was found to have an attractive interaction between the rotator and stator. This is evidenced by the Ag^+^–S distance of 3.260 Å (Figure 9) while the I^+^–S distance was 3.831 Å, and the N–Ag^+^–N angle was deformed from linearity to 174.6° while the N–I^+^–N angle was 179.3°. For **3-I** ^**+**^, this is a larger angle than the angle of 177.0° previously found by Lindblad et al. in the trapezoid analogue.^10a^ The stronger interaction in **3-Ag** ^**+**^ leads to a larger barrier to internal rotation of **3-Ag** ^**+**^ compared to **3-I** ^**+**^, 4.2 to 2.7 kcal/mol. This interaction of the central arene with the hexagonal macrocycle stator creates symmetrical energy wells on the potential energy surface (Figure 10) suggesting that the 2,1,3-benzothiadiazole rotator may not be able to move as quickly from one Ag^+^ to the Ag^+^ on the other side as **1-Ag** ^**+**^.

**Figure 9:**
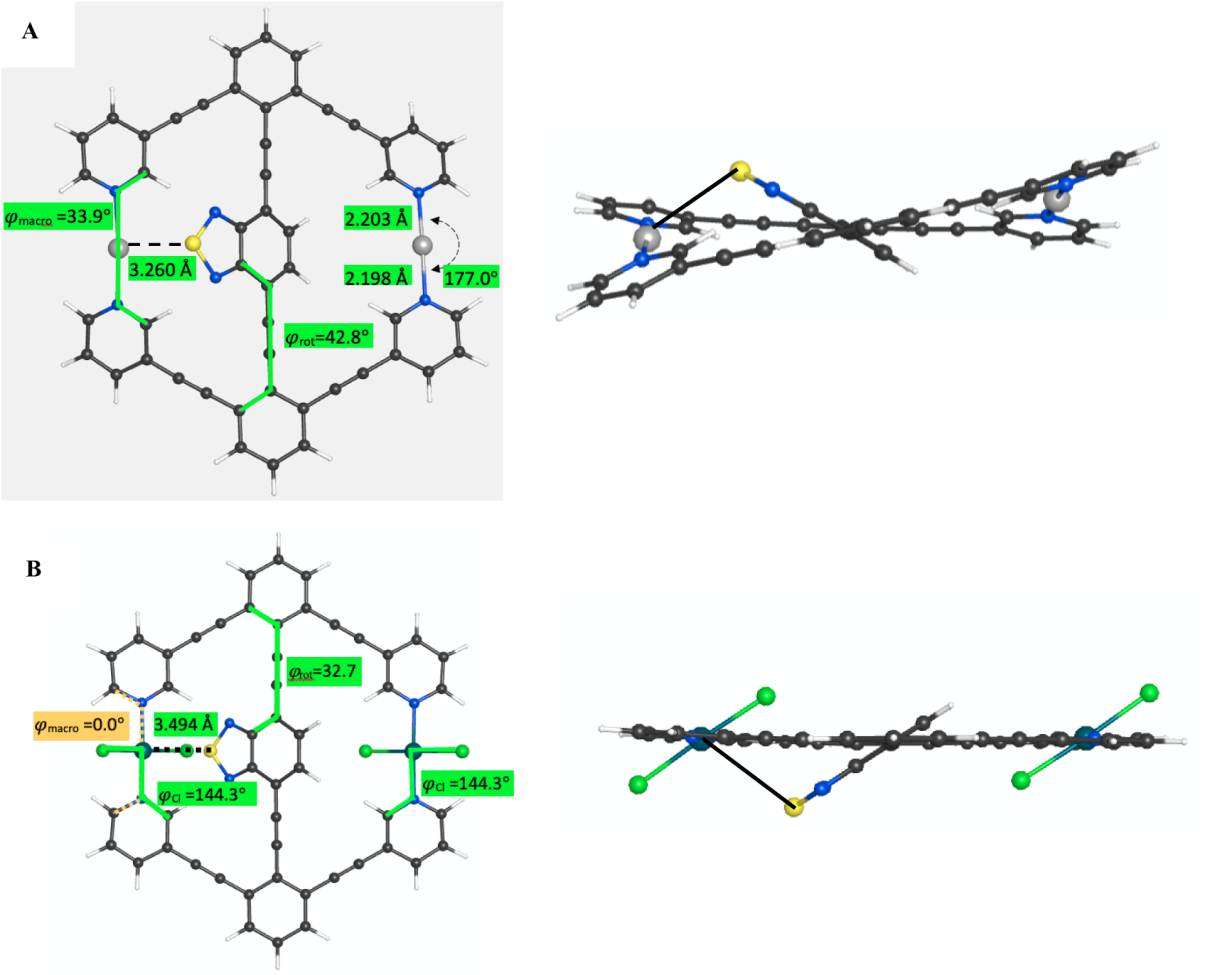
(A) Lowest energy conformer of **3-Ag** ^**+**^ with φ_rot_ dihedral of 42.8°, showing the interaction of the Ag^+^ and sulfur of the central arene as a dashed line with a distance of 3.260 Å. (B) Lowest energy conformer of **3-PdCl**_**2**_ with φ_rot_ dihedral of 32.7°, with both Cl–Pd–Cl planes parallel to the central arene and a Pd–S distance of 3.494 Å.

**Figure 10:**
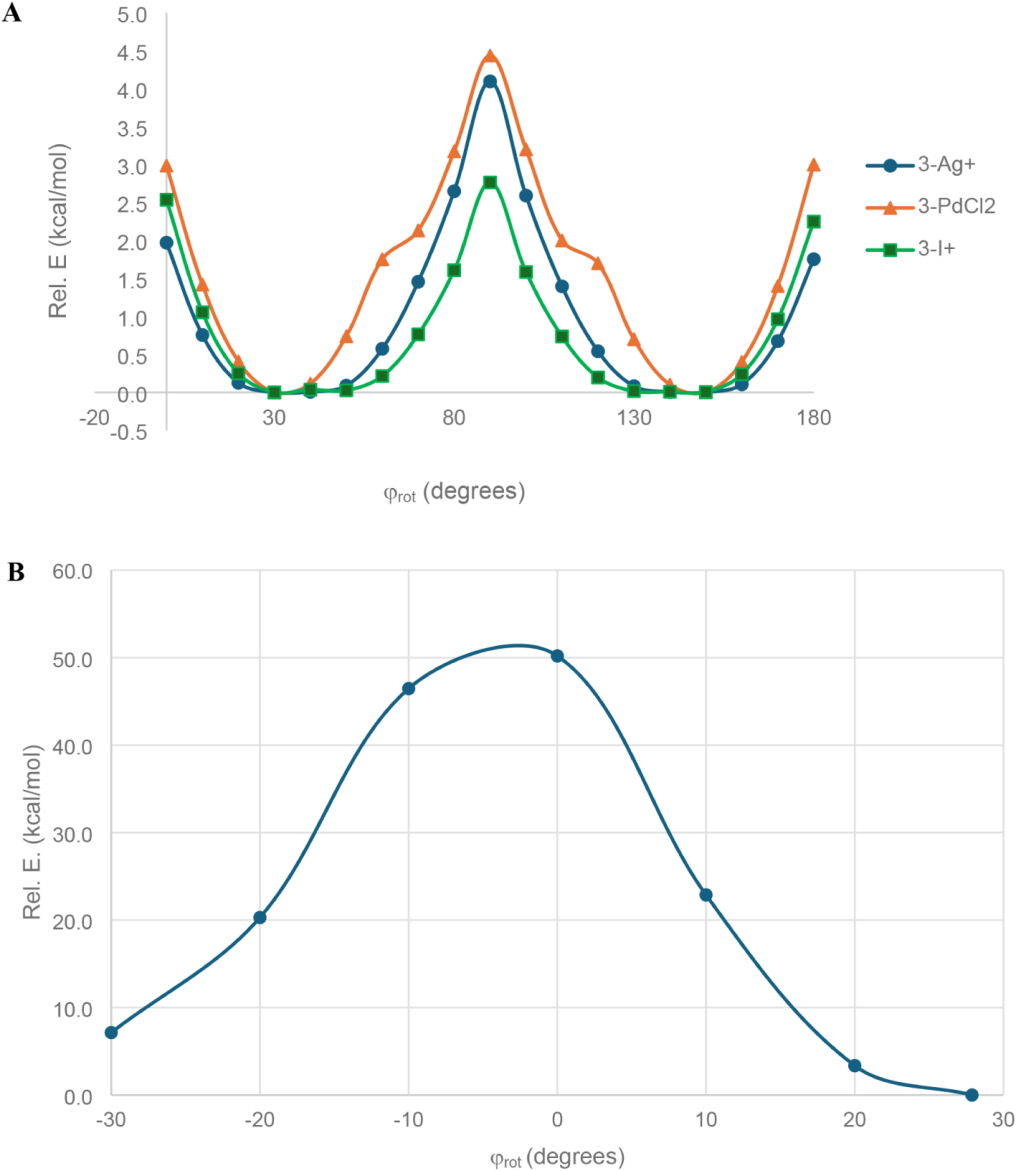
(A) Rotation potential energy surface of the central arene with respect to the benzene units of the macrocycle, measured by the φ_rot_ dihedral for **3-Ag** ^**+**^, **3-PdCl**_**2**_, and **3-I** ^**+**^. (B) Nonoptimized rotation potential energy surface of the central arene through the plane of the hexagonal macrocycle stator for **3-Ag** ^**+**^.

**3-PdCl**_**2**_ has two asymmetric minima. The minimum with φ_rot_ = 32.7° has both the Cl–Pd–Cl planes parallel to the central arene, as shown in Figure 9B. The other minimum at φ_rot_ = 120.9° has the near Cl–Pd–Cl plane perpendicular to the central arene. Steric hindrance, with an S–Cl distance of 3.252 Å, causes this minimum to be over 5 kcal/mol higher in energy than the φ_rot_ = 32.7° minimum.

As with **2-** systems, we could not locate a transition state for the central arene rotating through the plane of the macrocycle, and freezing with increasing φ_rot_ results in the macrocycle twisting instead of the central arene rotating through the macrocycle. For **3-Ag** ^**+**^, we performed calculations changing φ_rot_ dihedral but did not allow the rest of the molecule to relax (Figure 10B). This resulted in a barrier of 50.2 kcal/mol, suggesting that, for all **3-** systems, the size of the 2,1,3-benzothiadiazole rotator makes full rotation energetically unfeasible.

### **4-Ag** ^**+**^, **4-PdCl**_**2**_, and **4-I** ^***+***^ Systems

The minima of all three species showed slight twisting of the phenylalkynyl arms of the central arene to allow the phenyl groups to interact with the pyridine groups of the stator (Figure 11). This kind of stabilizing aryl–aryl interaction between the rotator and stator was observed to a lesser extent in Lindblad et al.’s study with trapezoid analogues of **3-Ag** ^**+**^ and another study where naphthalene was the central arene.^10a^ In **4-Ag** ^**+**^, the nearest C—C distance between the two rings is 3.116 Å. This may be the cause of the larger barriers to internal rotation: 8.3 kcal/mol for **4-PdCl**_**2**_, 6.2 kcal/mol for **4-Ag** ^**+**^, and 2.7 kcal/mol for **4-I** ^**+**^ (Figure 12). The symmetric minima that result from π-π stacking hint at the switching behavior that is obtainable when electron-rich and electron-poor groups, which are easily oxidized or reduced respectively, are placed on the ends of the extended alkyne rotator.^9^ Like the other PdCl_2_ systems, **4-PdCl**_**2**_ exhibited two asymmetric minima. A close contact of 2.726 Å exists between the chloro ligand and a hydrogen of the phenyl ring in the lowest energy conformer.

**Figure 11:**
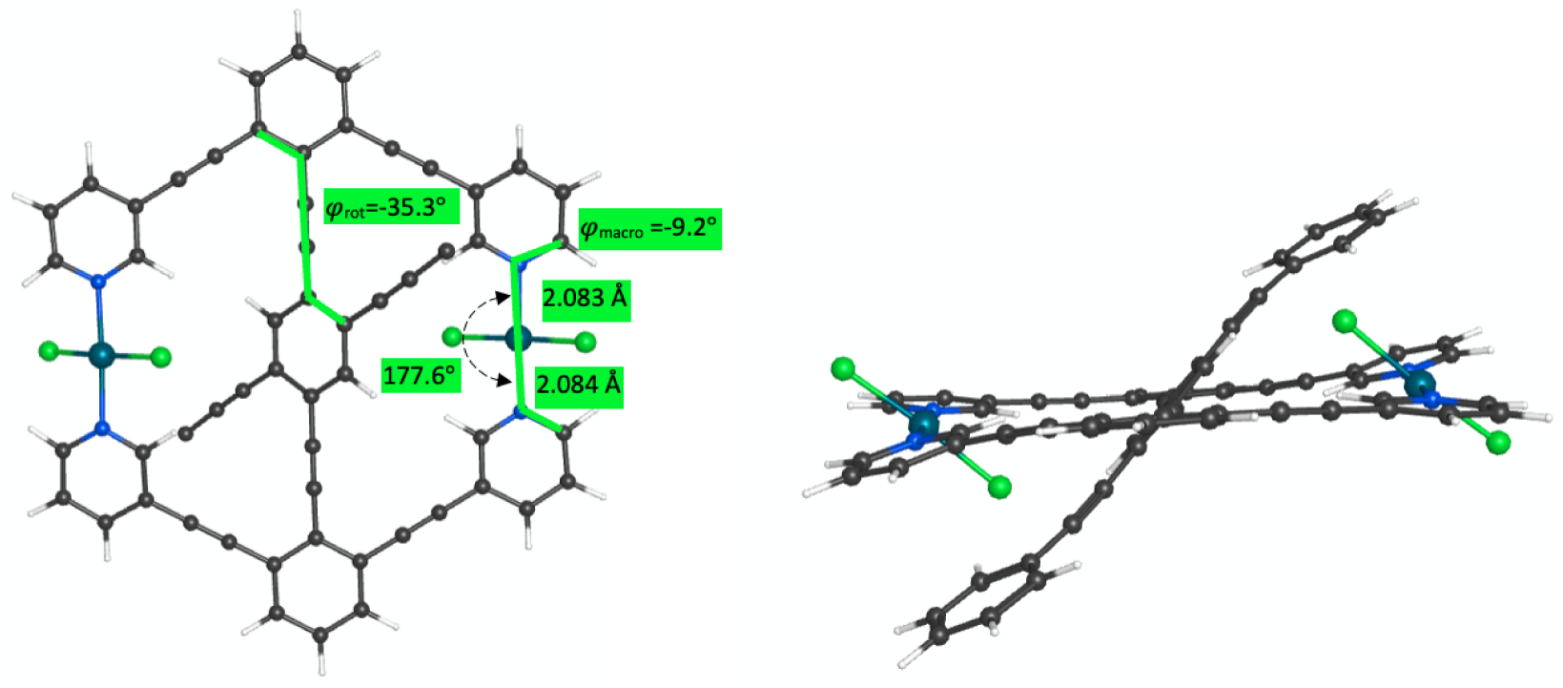
Top (phenyl groups removed for clarity) and side views of the φ_rot_ dihedral of the 43.6° conformer of **4-PdCl**_**2**_ showing deformation of phenylalkynyl groups of the central arene.

**Figure 12:**
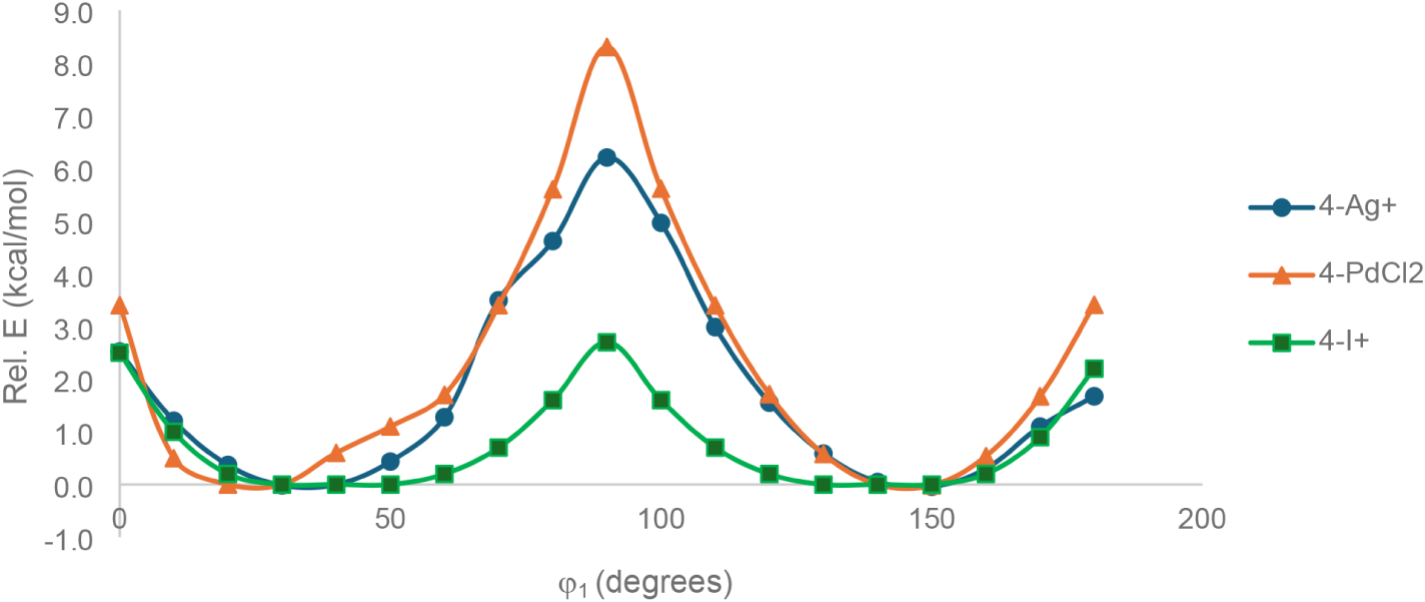
Rotation potential energy surface of the central arene with respect to the benzene units of the macrocycle, measured by φ_rot_ dihedral measurement for **4-Ag** ^**+**^, **4-PdCl**_**2**_, and **4-I** ^**+**^.

## Conclusion

The minima and rotation of four different central arenes in macrocycles formed by coordination to Ag^+^, PdCl_2_, and I^+^ were studied. Only the smallest central arene, a phenyl, was found to fully rotate through the macrocycle. In systems too large for full rotation, the Ag^+^ and I^+^ complexes of **3-** and **4-** display symmetric minima stabilized by S–Ag/S–I interactions and π–π stacking, respectively. Methoxy substituents on the rotator of **2-** make it incapable of full rotation but provide some conformer stabilization/minima, likely from O···HC interactions with the polarized hydrogens of the stator. For all compounds, the Cl–Pd–Cl units complicate minimum positions for the central arene rotator due to the positions of the Cl substituents relative to the central arene. Superficially, this raises the possibility of molecular gears in which the two Cl–Pd–Cl units move in concert with the central arene. Our calculations, however, seem to suggest that movement is mostly independent, with some conformations where the Cl–Pd–Cl plane is parallel to the arene and others where it is perpendicular.

## Supplementary Material



## Notes

Computed structures and energies for all compounds along with comparison of basis set and density functional (PDF)
