# Supplementary material for: Role of Central Arene Rotators and Ag + , I + , and PdCl2 Centers in Hexagonal Macrocycles as Molecular Rotors
Source: Inorg Chem. 2025 Nov 21;64(48):23525–34. doi: 10.1021/acs.inorgchem.5c03802 (PMC12690580; doi:10.1021/acs.inorgchem.5c03802)
Supplement: Supplementary file 1 [file ic5c03802_si_001.pdf]

Supporting Information

Role of Central Arene Rotators and Ag^{+}, I^{+}, and $PdCl_2$ centers in Hexagonal Macrocycles as Molecular Rotors

Nathaniel P. Litts,† Ayden K. Lutes,† Patrick A. Countryman,† Eric A. Green,† Alex I. P. Amore,† William C. Lyons,† Nathan J. France,† Alexander W. Litts, † Nathan P. Bowling,‡ Joseph D. Scanlon†*

†Department of Chemistry, Wabash College, 301 W Wabash Ave. Crawfordsville, IN 47933
‡Department of Chemistry and Biochemistry, University of Wisconsin-Stevens Point, 2101 Fourth Ave., Stevens Point, WI 54481

*email: scanlonj@wabash.edu

Table of Contents

1-Ag^+, $PdCl_2$, I^+ Structures page S2-S31
2-Ag^+, $PdCl_2$, I^+ Structures page S32-S37
3-Ag^+, $PdCl_2$, I^+ Structures page S38-S49
4-Ag^+, $PdCl_2$, I^+ Structures page S50-S64
Relative Energy Tables page S65-S67

1- Structures

1-Ag^+ $\varphi_1 = 65.6°$
E = -2430.68964504 Hartrees
G = -2430.163832 Hartrees
E(TZVPP) = -2433.085803 Hartrees

N	0.00000000	0.00000000	0.00000000
C	1.14128300	0.69413000	-0.06430700
C	1.19290500	2.08296300	0.11981700
C	-0.01194100	2.75478700	0.37956100
C	-1.19284800	2.02585000	0.44427700
C	-1.14866100	0.64999100	0.24998200
H	-2.06002200	0.05137800	0.29588200
H	-2.14656700	2.51331800	0.64399500
H	-0.00896200	3.83533900	0.52714800
C	2.44454800	2.76747600	0.03691200
C	3.51123800	3.34095600	-0.04087600
C	4.75737300	4.03832000	-0.14347100
C	5.96997800	3.31889500	-0.25635500
C	7.18258400	4.03832000	-0.36923800
C	7.16967700	5.44069300	-0.36991300
C	5.96997900	6.13550100	-0.25635400
C	4.77028000	5.44069300	-0.14279500
H	3.82567000	5.97745900	-0.05520800
H	5.96997900	7.22553100	-0.25635300
H	8.11428700	5.97745900	-0.45750000
C	8.42871800	3.34095500	-0.47183400
C	9.49540800	2.76747500	-0.54962200
C	10.7470510	2.08296100	-0.63252700
C	10.7986720	0.69412800	-0.44840400
N	11.9399550	-0.00000200	-0.51271100
C	13.0886160	0.64998900	-0.76269400
C	13.1328040	2.02584700	-0.95698900
C	11.9518970	2.75478500	-0.89227100
H	11.9489190	3.83533700	-1.03985800

H	14.0865230	2.51331500	-1.15670700
H	13.9999770	0.05137500	-0.80859400
Ag	11.9375310	-2.16752300	-0.21550100
N	11.9403350	-4.33887300	0.05066700
C	13.0908310	-4.99544600	0.27350000
C	13.1370790	-6.37679000	0.42316700
C	11.9566450	-7.10456500	0.33934800
C	10.7498190	-6.42584800	0.10832100
C	10.7991140	-5.03154600	-0.02856100
H	9.87612000	-4.47593700	-0.20747800
C	9.49843300	-7.10878900	0.01013100
C	8.43161500	-7.68108500	-0.07406000
C	7.18457000	-8.37805500	-0.16876300
C	5.96997600	-7.65876900	-0.25635500
C	5.96997600	-6.22680900	-0.25635500
C	5.96997600	-5.01210500	-0.25635500
C	5.96997600	-3.57655900	-0.25635500
C	5.39258800	-2.86386500	0.80952500
C	5.39200500	-1.47486200	0.80926700
C	5.96997700	-0.76219000	-0.25635500
C	5.96997800	0.67280800	-0.25635500
C	5.96997800	1.88758200	-0.25635500
C	6.54794900	-1.47486300	-1.32197700
C	6.54736500	-2.86386600	-1.32223600
H	6.98302900	-3.41428800	-2.15705900
H	6.98456600	-0.92423900	-2.15619900
H	4.95538800	-0.92423900	1.64348800
H	4.95692400	-3.41428700	1.64434800
C	4.75538200	-8.37805500	-0.34394800
C	4.76812900	-9.78050300	-0.34343400
C	5.96997600	-10.4753140	-0.25635500
C	7.17182300	-9.78050300	-0.16927600
H	8.11798800	-10.3171980	-0.09999900
H	5.96997600	-11.5653470	-0.25635400
H	3.82196300	-10.3171980	-0.41271000
C	3.50833600	-7.68108500	-0.43865000
C	2.44151900	-7.10878800	-0.52284100
C	1.19013300	-6.42584700	-0.62103000
C	1.14083900	-5.03154400	-0.48415000
H	2.06383400	-4.47593600	-0.30523400
N	-0.00038200	-4.33887100	-0.56337700
C	-1.15087900	-4.99544400	-0.78620800
C	-1.19712700	-6.37678800	-0.93587400
C	-0.01669400	-7.10456400	-0.85205500
H	-0.01553000	-8.18943300	-0.96355600
H	-2.15215800	-6.86949200	-1.11544700

H -2.06198400 -4.39772800 -0.84658200
Ag 0.00242300 -2.16752100 -0.29721000
H 11.9554810 -8.18943500 0.45084900
H 14.0921090 -6.86949500 0.60274100
H 14.0019370 -4.39773000 0.33387400
H 9.87782400 0.14446900 -0.24182400
H 2.06213100 0.14447100 -0.27088600

1-Ag^+ $\varphi_1 = 123.8°$
E = -2430.689645 Hartrees
G = -2430.163832 Hartrees
E(TZVPP) = -2433.085803 Hartrees
N 0.00000000 0.00000000 0.00000000
C 1.14124100 0.69263900 -0.07921300
C 1.19055600 2.08695200 0.05754500
C -0.01627100 2.76571700 0.28842700
C -1.19672600 2.03797700 0.37223100
C -1.15049800 0.65661800 0.22269300
H -2.06162100 0.05892700 0.28305300
H -2.15175800 2.53071900 0.55169700
H -0.01509000 3.85059700 0.39983000
C 2.44196200 2.76985900 -0.04061300
C 3.50878800 3.34214700 -0.12475400
C 4.75584200 4.03910900 -0.21939800
C 5.97044000 3.31981500 -0.30688500
C 7.18504400 4.03909300 -0.39443200
C 7.17229600 5.44154200 -0.39398400
C 5.97044600 6.13636000 -0.30700800
C 4.76859300 5.44155600 -0.21997000
H 3.82242500 5.97825700 -0.15077900
H 5.97045000 7.22638700 -0.30705400
H 8.11846600 5.97823400 -0.46322400
C 8.43209400 3.34211700 -0.48900300
C 9.49892000 2.76981800 -0.57307200
C 10.7503200 2.08687800 -0.67112000
C 10.7995990 0.69256200 -0.53432900
N 11.9408400 -0.00009600 -0.61343600
C 13.0913640 0.65650300 -0.83604900
C 13.1376250 2.03785700 -0.98560900
C 11.9571760 2.76561700 -0.90191600
H 11.9560230 3.85049500 -1.01334400
H 14.0926800 2.53057800 -1.16501100
H 14.0024830 0.05879900 -0.89633000
Ag 11.9380200 -2.17147800 -0.34753500
N 11.9404060 -4.33903600 -0.05063800
C 13.0890540 -4.98908700 0.19925700

C	13.1332100	-6.36496500	0.39340600
C	11.9522840	-7.09386400	0.32863400
C	10.7474490	-6.42198200	0.06898200
C	10.7990990	-5.03312900	-0.11499600
H	9.87826200	-4.48342600	-0.32150600
C	9.49579100	-7.10646300	-0.01393200
C	8.42909400	-7.67993300	-0.09169400
C	7.18295100	-8.37728700	-0.19421200
C	5.97033500	-7.65784900	-0.30690200
C	5.97034900	-6.22653400	-0.30681600
C	5.97037600	-5.01176100	-0.30676500
C	5.97040300	-3.57676200	-0.30675200
C	6.54834900	-2.86409600	0.75888800
C	6.54777900	-1.47509100	0.75914400
C	5.97043100	-0.76239600	-0.30675800
C	5.97043100	0.67315100	-0.30677400
C	5.97043200	1.88785400	-0.30681800
C	5.39307600	-1.47508500	-1.37265900
C	5.39248000	-2.86408600	-1.37239800
H	4.95588600	-3.41470500	-2.20663500
H	4.95744400	-0.92466100	-2.20749800
H	6.98342500	-0.92467200	1.59397800
H	6.98493700	-3.41472000	1.59312400
C	4.75771300	-8.37726200	-0.41968000
C	4.77061400	-9.77963500	-0.42044300
C	5.97032200	-10.4744560	-0.30707500
C	7.17003700	-9.77966100	-0.19361900
H	8.11465500	-10.3164360	-0.10618000
H	5.97031300	-11.5644840	-0.30713900
H	3.82599100	-10.3163900	-0.50794200
C	3.51156600	-7.67989100	-0.52209400
C	2.44486500	-7.10641400	-0.59974800
C	1.19320700	-6.42191200	-0.68252400
C	1.14161600	-5.03305400	-0.49858800
H	2.06249800	-4.48337000	-0.29222800
N	0.00032200	-4.33893200	-0.56280600
C	-1.14838000	-4.98895900	-0.81250900
C	-1.19260000	-6.36484400	-1.00660000
C	-0.01168300	-7.09377300	-0.94197300
H	-0.00872900	-8.17434600	-1.08941000
H	-2.14635100	-6.85234000	-1.20609800
H	-2.05974700	-4.39035100	-0.85834800
Ag	0.00278400	-2.17137600	-0.26590000
H	11.9492800	-8.17443000	0.47611300
H	14.0869190	-6.85247800	0.59306100
H	14.0004310	-4.39050200	0.24520400

H 9.87658200 0.13693700 -0.35557800
H 2.06423600 0.13699600 -0.25802600

TS 1-Ag^+ φ_1 = 175.4° ifreq= 56.9 cm^{-1}
E = -2430.681357 Hartrees
G = -2430.15789 Hartrees
E(TZVPP) = -2433.07750692 Hartrees
N 0.00000000 0.00000000 0.00000000
C -1.16230200 0.63116200 -0.20262300
C -1.40877500 1.93546200 0.24849900
C -0.36681400 2.60241800 0.91291600
C 0.84386700 1.94821100 1.10312000
C 0.98848300 0.64441100 0.64131400
H 1.92367800 0.10160000 0.78937100
H 1.67405700 2.43576700 1.61325700
H -0.51905200 3.62008300 1.27434400
C -2.68860400 2.54177700 0.04349800
C -3.77832500 3.05617900 -0.10541800
C -5.05360700 3.68322700 -0.29463600
C -6.22984500 2.89933500 -0.37692400
C -7.47053300 3.54334400 -0.59962000
C -7.52235300 4.93997900 -0.71336900
C -6.36147700 5.70021700 -0.61324100
C -5.13353200 5.07803300 -0.40907300
H -4.21936800 5.66770300 -0.34040000
H -6.41300100 6.78546500 -0.70109200
H -8.48559800 5.42104600 -0.88310600
C -8.67090500 2.77260900 -0.72961300
C -9.69279000 2.13113100 -0.86515400
C -10.8806500 1.35357200 -1.03542600
C -10.8429090 -0.03101800 -0.82096000
N -11.9113080 -0.82110100 -0.97065000
C -13.0789740 -0.27232200 -1.34447100
C -13.2127750 1.09219200 -1.57738600
C -12.1063350 1.91853700 -1.42202200
H -12.1776780 2.99249400 -1.59782000
H -14.1790440 1.49583500 -1.87832000
H -13.9310880 -0.94412900 -1.46078200
Ag -11.7446310 -2.96889500 -0.54994700
N -11.5103050 -5.08184900 0.00016100
C -12.4988140 -5.72631100 0.64138300
C -12.3542200 -7.03015700 1.10306500
C -11.1435390 -7.68435500 0.91283400
C -10.1015530 -7.01734300 0.24851500
C -10.3480020 -5.71299700 -0.20248200
H -9.57054100 -5.17789700 -0.75057300

C -8.82171900 -7.62363900 0.04350100
C -7.73198900 -8.13800100 -0.10547600
C -6.45670100 -8.76501200 -0.29477200
C -5.28045900 -7.98111900 -0.37699500
C -5.35475900 -6.56312400 -0.23884900
C -5.44642600 -5.35821500 -0.11504000
C -5.59467400 -3.93953600 -0.02687200
C -6.87750500 -3.36657200 0.00279100
C -7.03533600 -1.99003300 -0.00036400
C -5.91552500 -1.14224800 -0.02689100
C -6.06380700 0.27642800 -0.11505700
C -6.15552500 1.48133400 -0.23884700
C -4.63268700 -1.71521000 0.00260400
C -4.47485800 -3.09175200 -0.00052600
H -3.47970400 -3.53432300 0.01059000
H -3.76615500 -1.05507600 0.02160500
H -8.03048800 -1.54745900 0.01085700
H -7.74403200 -4.02670600 0.02193100
C -4.03977100 -8.62512100 -0.59971600
C -3.98795700 -10.0217480 -0.71356500
C -5.14884000 -10.7819870 -0.61351700
C -6.37678200 -10.1598100 -0.40931700
H -7.29094900 -10.7494810 -0.34069500
H -5.09732100 -11.8672280 -0.70145300
H -3.02471200 -10.5028070 -0.88332100
C -2.83938900 -7.85439000 -0.72963900
C -1.81747600 -7.21294300 -0.86512400
C -0.62956700 -6.43543600 -1.03529200
C -0.66734300 -5.05079000 -0.82117800
H -1.60568700 -4.58485000 -0.51921900
N 0.40112500 -4.26076900 -0.97071900
C 1.56888800 -4.80966800 -1.34405900
C 1.70272100 -6.17423600 -1.57663500
C 0.59621800 -7.00051900 -1.42139700
H 0.66759000 -8.07452000 -1.59691400
H 2.66906800 -6.57797200 -1.87719100
H 2.42106000 -4.13791400 -1.46025200
Ag 0.23446300 -2.11293000 -0.55017800
H -10.9913200 -8.70205600 1.27417000
H -13.1844280 -7.51775500 1.61313400
H -13.4340060 -5.18350400 0.78946700
H -9.90465100 -0.49686400 -0.51859100
H -1.93973500 0.09611400 -0.75080800

Internal TS 1-Ag^+ $\varphi_1 = 96.073°$ ifreq= 21.3 cm^{-1}
E = -2430.689391 Hartrees

G = -2430.165565 Hartrees
E(TZVPP) = -2433.08571576 Hartrees

N	0.00000000	0.00000000	0.00000000
C	1.13591900	-0.70023100	0.08847900
C	1.18686200	-2.08670800	-0.11276400
C	-0.01220200	-2.74911500	-0.41895200
C	-1.18722800	-2.01348200	-0.51061900
C	-1.14314000	-0.64083700	-0.29496000
H	-2.05003100	-0.03725400	-0.36028300
H	-2.13632300	-2.49340200	-0.74722100
H	-0.00942800	-3.82754100	-0.58134000
C	2.43277100	-2.77741600	-0.00029200
C	3.49621900	-3.35300500	0.10143600
C	4.74007100	-4.05108200	0.22596100
C	5.95096300	-3.33187100	0.35327900
C	7.16185700	-4.05107700	0.48059300
C	7.14920800	-5.45355800	0.48069400
C	5.95096900	-6.14835100	0.35327000
C	4.75272700	-5.45356200	0.22585100
H	3.80920500	-5.99019500	0.12645400
H	5.95097100	-7.23838800	0.35326600
H	8.09273200	-5.99018700	0.58008800
C	8.40570500	-3.35299500	0.60512500
C	9.46915300	-2.77740600	0.70685900
C	10.7150640	-2.08670400	0.81933700
C	10.7660110	-0.70022600	0.61809900
N	11.9019310	0.00000300	0.70658700
C	13.0450680	-0.64083800	1.00155000
C	13.0891520	-2.01348400	1.21720300
C	11.9141260	-2.74911400	1.12552800
H	11.9113490	-3.82754100	1.28791200
H	14.0382450	-2.49340700	1.45380900
H	13.9519600	-0.03725700	1.06688100
Ag	11.9011130	2.15892500	0.35337300
N	11.9019660	4.31784300	0.00012600
C	13.0450950	4.95867500	-0.29488900
C	13.0891620	6.33130800	-0.51063100
C	11.9141270	7.06693200	-0.41900500
C	10.7150740	6.40453000	-0.11276100
C	10.7660410	5.01806800	0.08858300
H	9.84899600	4.47589800	0.32917400
C	9.46915200	7.09522200	-0.00033100
C	8.40569500	7.67080100	0.10136800
C	7.16184300	8.36887700	0.22589400
C	5.95095500	7.64966700	0.35326400
C	5.95095500	6.21688600	0.35327200

C	5.95095700	5.00226300	0.35327700
C	5.95096000	3.56554900	0.35328100
C	5.95240600	2.85346900	-0.85919800
C	5.95135100	1.46433400	-0.85919700
C	5.95096300	0.75225900	0.35328500
C	5.95096200	-0.68446100	0.35328600
C	5.95096200	-1.89908400	0.35328300
C	5.95057300	1.46433800	1.56576500
C	5.94951600	2.85347300	1.56576200
H	5.94832900	3.40304100	2.50771700
H	5.95040800	0.91477300	2.50772200
H	5.95151600	0.91476600	-1.80115200
H	5.95359300	3.40303400	-1.80115500
C	4.74006600	8.36887700	0.48062800
C	4.75271800	9.77135700	0.48072600
C	5.95095200	10.4661480	0.35324500
C	7.14918800	9.77135800	0.22577600
H	8.09270700	10.3079890	0.12634100
H	5.95095100	11.5561850	0.35323700
H	3.80919900	10.3079880	0.58015700
C	3.49621600	7.67080000	0.60517500
C	2.43276100	7.09522200	0.70689500
C	1.18684400	6.40452700	0.81935400
C	1.13587800	5.01806600	0.61800600
H	2.05291800	4.47590100	0.37738400
N	-0.00004100	4.31783500	0.70649500
C	-1.14316400	4.95866000	1.00154600
C	-1.18723200	6.33129200	1.21729300
C	-0.01220300	7.06692200	1.12563500
H	-0.00941500	8.14533600	1.28810100
H	-2.13631400	6.81120400	1.45396200
H	-2.05005600	4.35507900	1.06686900
Ag	0.00081600	2.15892000	0.35322900
H	11.9113370	8.14534700	-0.58146700
H	14.0382480	6.81122600	-0.74727000
H	13.9519920	4.35509900	-0.36018600
H	9.84895200	-0.15804900	0.37757500
H	2.05298100	-0.15805800	0.32900200

1-Ag^{+} $\varphi_1 = 0.0°$
E = -2430.684400 Hartrees

N	0.00000000	0.00000000	0.00000000
C	1.06538000	0.78028000	0.21054500
C	1.11397800	2.12450500	-0.18402800
C	-0.00980000	2.65435800	-0.83668500
C	-1.10918100	1.83432500	-1.06193100

C	-1.07076500	0.51381100	-0.62774600
H	-1.92163400	-0.15089000	-0.78657500
H	-1.99773700	2.21019600	-1.56823600
H	-0.00948600	3.69607600	-1.15950700
C	2.28474600	2.89555300	0.09669600
C	3.29437500	3.51478600	0.36181200
C	4.49339500	4.23062300	0.68006100
C	5.67794100	3.51712300	0.98006800
C	6.85438100	4.23828600	1.29275800
C	6.83296800	5.64003400	1.30479400
C	5.66175400	6.32966200	1.00636900
C	4.49854600	5.63243200	0.69445100
H	3.57847000	6.16745200	0.45930300
H	5.65553500	7.41965200	1.01667200
H	7.74700500	6.18108800	1.54950000
C	8.06296100	3.53168000	1.59507800
C	9.08306100	2.92342400	1.84519200
C	10.2683370	2.16861300	2.10860800
C	10.3341670	0.82762300	1.70566300
N	11.4140160	0.06329000	1.90059400
C	12.4832810	0.59075000	2.51949000
C	12.5054620	1.90936600	2.96054100
C	11.3906190	2.71272800	2.75213900
H	11.3771110	3.75248500	3.08094600
H	13.3934600	2.29674500	3.45909300
H	13.3463020	-0.06110400	2.66543400
Ag	11.4466770	-1.97207300	1.08909500
N	11.4081660	-3.95072000	0.14524700
C	12.3977030	-4.34335100	-0.67422900
C	12.3690780	-5.56668800	-1.33498300
C	11.2792560	-6.40972100	-1.15318400
C	10.2399470	-6.00599100	-0.30105100
C	10.3610580	-4.76116500	0.33351600
H	9.57634100	-4.42957800	1.01709800
C	9.07935300	-6.80424400	-0.05656400
C	8.07694900	-7.44073700	0.19465900
C	6.89449000	-8.16065100	0.56209400
C	5.76654900	-7.44441700	1.02916900
C	5.74658400	-6.01731600	0.98740900
C	5.70812800	-4.80548200	0.90838500
C	5.67995100	-3.37580000	0.86820400
C	4.61386500	-2.66990700	1.45419600
C	4.62071000	-1.28302100	1.49011300
C	5.68879000	-0.55861700	0.93201000
C	5.68757700	0.87303400	0.95232400
C	5.68500100	2.08766200	0.96607300

C 6.76092300 -1.26456900 0.35881100
C 6.76746900 -2.65196200 0.34850300
H 7.59486000 -3.19744000 -0.10742100
H 7.57993300 -0.70520800 -0.09534600
H 3.81211400 -0.73927900 1.98068700
H 3.79954100 -3.23035300 1.91532900
C 4.61546300 -8.16411500 1.42978800
C 4.62417100 -9.56553900 1.41059200
C 5.74726300 -10.2569340 0.96584100
C 6.87384200 -9.56158600 0.53620800
H 7.75457700 -10.0979680 0.18311600
H 5.74194600 -11.3468210 0.94741000
H 3.73237600 -10.1050800 1.72952900
C 3.42585600 -7.44956100 1.78478000
C 2.41350100 -6.82349600 2.02178300
C 1.24160300 -6.03736400 2.25260700
C 1.10091800 -4.80745900 1.59368100
H 1.87678300 -4.48038900 0.89799000
N 0.04509800 -4.00615700 1.77218600
C -0.93581500 -4.39568800 2.60338600
C -0.88932400 -5.60629500 3.28623900
C 0.21095000 -6.43821400 3.11646300
H 0.28334200 -7.39053800 3.64305000
H -1.70952000 -5.88476000 3.94724300
H -1.78339800 -3.71907600 2.72539600
Ag -0.01121900 -2.03559800 0.81172700
H 11.2218560 -7.37315600 -1.66104600
H 13.1954170 -5.84689700 -1.98754400
H 13.2375640 -3.65876800 -0.80481400
H 9.47311400 0.37646000 1.20959900
H 1.92841800 0.33994700 0.71288000

1-Ag^+ $\varphi_1 = 60.0°$
E = -2430.6896382 Hartrees
N 0.00000000 0.00000000 0.00000000
C 1.14772100 -0.68515700 -0.04270300
C 1.19488000 -2.08510800 0.01344000
C -0.02136900 -2.77793400 0.12032300
C -1.20865700 -2.05811600 0.16606100
C -1.15955500 -0.67025000 0.10314000
H -2.07584200 -0.07837300 0.13643500
H -2.17106500 -2.56198600 0.24963800
H -0.02193300 -3.86752400 0.16668500
C 2.45287200 -2.76091000 -0.03820600
C 3.52355100 -3.33066900 -0.08034500
C 4.77369400 -4.02712600 -0.12387800

C	5.99113600	-3.30823400	-0.15964200
C	7.20801700	-4.02808300	-0.19537000
C	7.19469800	-5.43055800	-0.19481500
C	5.99002900	-6.12495900	-0.15957900
C	4.78590700	-5.42961200	-0.12437200
H	3.83741600	-5.96587800	-0.09561400
H	5.98960300	-7.21498900	-0.15955200
H	8.14276600	-5.96757300	-0.22354200
C	8.45872900	-3.33264200	-0.23891900
C	9.52990800	-2.76381800	-0.28099600
C	10.7885220	-2.08915400	-0.33248900
C	10.8370160	-0.68926100	-0.27595200
N	11.9853880	-0.00518600	-0.31853300
C	13.1442990	-0.67651400	-0.42193400
C	13.1920790	-2.06440700	-0.48523500
C	12.0041070	-2.78310400	-0.43962500
H	12.0036350	-3.87268200	-0.48628700
H	14.1540010	-2.56917100	-0.56900900
H	14.0611490	-0.08550400	-0.45514100
Ag	11.9809280	2.17906400	-0.19669500
N	11.9877620	4.36160700	-0.04635300
C	13.1468570	5.02880200	0.07948100
C	13.1954750	6.41430300	0.18237500
C	12.0080720	7.13487500	0.15532500
C	10.7923550	6.44526100	0.02438000
C	10.8401100	5.04763600	-0.07408100
H	9.91071300	4.48377400	-0.17915000
C	9.53431700	7.12195500	-0.01252000
C	8.46372200	7.69229200	-0.04888100
C	7.21391700	8.38875600	-0.10038700
C	5.99744100	7.66956000	-0.15904900
C	5.99722000	6.23801400	-0.15870800
C	5.99716500	5.02328200	-0.15922600
C	5.99644300	3.58803100	-0.15963500
C	5.43969600	2.87599300	-1.23688900
C	5.43848200	1.48697100	-1.23694200
C	5.99353200	0.77381800	-0.15956800
C	5.99244400	-0.66168800	-0.15965400
C	5.99175100	-1.87638700	-0.15965600
C	6.54974000	1.48587000	0.91793900
C	6.55115800	2.87488500	0.91794000
H	6.97106300	3.42499500	1.76100100
H	6.96829800	0.93501800	1.76117200
H	5.01866900	0.93695600	-2.08009700
H	5.02055600	3.42693700	-2.07978500
C	4.78136200	8.38923700	-0.21970000

C 4.79456500 9.79168200 -0.22163200
C 5.99817000 10.4863160 -0.16196500
C 7.20144000 9.79120000 -0.10108600
H 8.14915700 10.3276790 -0.05553400
H 5.99843700 11.5763440 -0.16300900
H 3.84713100 10.3285540 -0.26843400
C 3.53115100 7.69343800 -0.27036900
C 2.46001000 7.12408400 -0.30608700
C 1.20111600 6.44893100 -0.34208900
C 1.15159700 5.05136000 -0.24375700
H 2.08032200 4.48624500 -0.13948800
N 0.00300500 4.36686200 -0.27066400
C -1.15530400 5.03560000 -0.39549100
C -1.20217600 6.42117300 -0.49820200
C -0.01379800 7.14016100 -0.47201900
H -0.01254100 8.22791600 -0.55044800
H -2.16368100 6.92390200 -0.59757000
H -2.07242900 4.44436600 -0.41376200
Ag 0.00694700 2.18427500 -0.12101600
H 12.0081930 8.22262200 0.23387400
H 14.1575660 6.91574800 0.28255800
H 14.0631830 4.43635200 0.09843100
H 9.90752200 -0.12179100 -0.19399900
H 2.07776400 -0.11855900 -0.12445700

1-Ag^+ $\varphi_1 = 120.0°$
E = -2430.68963824 Hartrees
N 0.00000000 0.00000000 0.00000000
C -1.14830000 -0.68414900 -0.04352300
C -1.19675700 -2.08403600 0.01313800
C 0.01877500 -2.77790500 0.12145200
C 1.20665900 -2.05913300 0.16804900
C 1.15885200 -0.67124700 0.10453300
H 2.07564000 -0.08018300 0.13849200
H 2.16853900 -2.56382200 0.25275800
H 0.01831900 -3.86747700 0.16824400
C -2.45528400 -2.75877900 -0.03942100
C -3.52639600 -3.32766900 -0.08231100
C -4.77702400 -4.02320200 -0.12674800
C -5.99394100 -3.30342200 -0.16276100
C -7.21132000 -4.02238700 -0.19931800
C -7.19900700 -5.42487000 -0.19938900
C -5.99485100 -6.12014500 -0.16393500
C -4.79024600 -5.42567700 -0.12786300
H -3.84215500 -5.96264000 -0.09890500
H -5.99520100 -7.21017600 -0.16440600

H	-8.14744600	-5.96119500	-0.22877300
C	-8.46148100	-3.32597600	-0.24301100
C	-9.53215400	-2.75620500	-0.28513100
C	-10.7901020	-2.08030800	-0.33658500
C	-10.8372040	-0.68041100	-0.27909100
N	-11.9848770	0.00484900	-0.32152500
C	-13.1444280	-0.66523800	-0.42574200
C	-13.1935820	-2.05304200	-0.49004100
C	-12.0063500	-2.77296700	-0.44457800
H	-12.0069530	-3.86251300	-0.49200400
H	-14.1559890	-2.55677200	-0.57446200
H	-14.0606740	-0.07328300	-0.45879400
Ag	-11.9779750	2.18897500	-0.19809600
N	-11.9821850	4.37140300	-0.04631300
C	-13.1403830	5.03992400	0.08059800
C	-13.1872860	6.42545200	0.18397500
C	-11.9990560	7.14461600	0.15625000
C	-10.7842420	6.45360400	0.02418600
C	-10.8337180	5.05607200	-0.07465700
H	-9.90507400	4.49112900	-0.18057100
C	-9.52549400	7.12893400	-0.01354500
C	-8.45441400	7.69830900	-0.05070500
C	-7.20427500	8.39409800	-0.10322300
C	-5.98811800	7.67437400	-0.16171400
C	-5.98827600	6.24283100	-0.16008900
C	-5.98838300	5.02809900	-0.15967900
C	-5.98908000	3.59285100	-0.15993800
C	-6.54577400	2.88027800	0.91699300
C	-6.54691300	1.49126000	0.91641000
C	-5.99183500	0.77863000	-0.16129600
C	-5.99281600	-0.65687600	-0.16181800
C	-5.99342500	-1.87157500	-0.16224100
C	-5.43569800	1.49121200	-1.23848900
C	-5.43434800	2.88023000	-1.23785100
H	-5.01447300	3.43074700	-2.08066200
H	-5.01711400	0.94077900	-2.08198300
H	-6.96667200	0.94083600	1.75932600
H	-6.96491700	3.43080700	1.76015900
C	-4.77176200	8.39352600	-0.22347400
C	-4.78437800	9.79596400	-0.22661300
C	-5.98768600	10.4911260	-0.16707400
C	-7.19120700	9.79654400	-0.10513900
H	-8.13868600	10.3334550	-0.05970100
H	-5.98752700	11.5811530	-0.16905100
H	-3.83674500	10.3324140	-0.27424400
C	-3.52193600	7.69703300	-0.27403200

C -2.45129600 7.12673800 -0.30972400
C -1.19318400 6.45014500 -0.34595200
C -1.14529000 5.05259100 -0.24661700
H -2.07463700 4.48868300 -0.14135100
N 0.00245800 4.36667400 -0.27375100
C 1.16148800 5.03392200 -0.39978900
C 1.20996900 6.41937300 -0.50349900
C 0.02248500 7.13982100 -0.47710300
H 0.02248700 8.22752100 -0.55630300
H 2.17202600 6.92085900 -0.60380700
H 2.07788400 4.44156600 -0.41824200
Ag -0.00439900 2.18420900 -0.12241000
H -11.9978230 8.23234000 0.23511800
H -14.1487120 6.92799700 0.28503500
H -14.0573970 4.44855500 0.10004000
H -9.90716000 -0.11394000 -0.19647700
H -2.07775100 -0.11674100 -0.12638100

1-Ag^+ $\varphi_1 = 180.0°$
E = -2430.68472437 Hartrees
N 0.00000000 0.00000000 0.00000000
C -1.06719600 0.78903100 0.16286700
C -1.09854300 2.12393300 -0.26370300
C 0.04581000 2.63454200 -0.89563700
C 1.14772100 1.80558100 -1.07000400
C 1.09077900 0.49529300 -0.60782100
H 1.94272000 -0.17622700 -0.72717400
H 2.05233000 2.16653900 -1.55842100
H 0.05936700 3.66856900 -1.24202800
C -2.27330200 2.90536000 -0.03376100
C -3.28681900 3.53402400 0.19112100
C -4.48700800 4.26489400 0.46737800
C -5.67676600 3.56808100 0.78439800
C -6.85143200 4.30571100 1.06419100
C -6.82414100 5.70676900 1.02258900
C -5.64843600 6.37976300 0.70440300
C -4.48617900 5.66632600 0.42806400
H -3.56188100 6.18807500 0.18001000
H -5.63767400 7.46930200 0.67275600
H -7.73696800 6.26053100 1.24206000
C -8.06329700 3.61692500 1.39365500
C -9.08521300 3.02467200 1.67305700
C -10.2746290 2.29203000 1.97764400
C -10.3612090 0.93830300 1.62408100
N -11.4471760 0.19465500 1.86094800
C -12.5012940 0.75639900 2.47556200

C	-12.5022390	2.08998000	2.86954100
C	-11.3815130	2.87203400	2.61657800
H	-11.3513890	3.92268600	2.90750700
H	-13.3784190	2.50545100	3.36638300
H	-13.3694810	0.12039300	2.65665600
Ag	-11.5251050	-1.86132300	1.10604100
N	-11.5481550	-3.85283100	0.18866100
C	-12.5774570	-4.24117600	-0.58245800
C	-12.5836490	-5.46234800	-1.24772100
C	-11.4866750	-6.30657000	-1.12433700
C	-10.4059300	-5.90728800	-0.32299300
C	-10.4949700	-4.66581100	0.32344100
H	-9.67843600	-4.33895800	0.97106000
C	-9.23353400	-6.70551000	-0.14570100
C	-8.21794800	-7.34361200	0.04013400
C	-7.01665300	-8.07017600	0.32229000
C	-5.90414100	-7.38239900	0.86370700
C	-5.92857500	-5.96143900	1.00300800
C	-5.92214700	-4.74766000	1.05820300
C	-5.88709400	-3.31745300	1.04462700
C	-6.90509300	-2.58870000	0.40335900
C	-6.84381400	-1.20451400	0.33069500
C	-5.76803200	-0.50539200	0.90629500
C	-5.72221700	0.92508000	0.85746700
C	-5.69504200	2.13900000	0.82298100
C	-4.74115900	-1.23485700	1.53044200
C	-4.78935200	-2.62076000	1.57962700
H	-3.99912200	-3.18409100	2.07797400
H	-3.91554500	-0.69480100	1.99586600
H	-7.61591400	-0.64423300	-0.19863900
H	-7.72621300	-3.13022200	-0.06834000
C	-4.73039200	-8.11205100	1.16828900
C	-4.70152300	-9.50108100	0.98334600
C	-5.81021200	-10.1668090	0.46866800
C	-6.95882400	-9.45722300	0.13105000
H	-7.82807000	-9.97215500	-0.27798900
H	-5.77584200	-11.2464500	0.32239100
H	-3.79214400	-10.0491170	1.22998900
C	-3.55402500	-7.41376500	1.59408400
C	-2.54889700	-6.80049900	1.88817400
C	-1.37893600	-6.02980000	2.17598300
C	-1.21439500	-4.78058400	1.56064500
H	-1.97577100	-4.42153900	0.86464500
N	-0.15234300	-3.99822600	1.78168800
C	0.80775000	-4.42332800	2.61971300
C	0.73390300	-5.65230300	3.26622600

C   -0.37038200 -6.46758500 3.04785300
H   -0.46227800 -7.43539200 3.54221000
H   1.53714800 -5.95919700 3.93540100
H   1.66070400 -3.76091400 2.77711700
Ag -0.03388100 -2.02497900 0.83480100
H   -11.4551300 -7.26753700 -1.63906700
H   -13.4419370 -5.73937900 -1.85909100
H   -13.4214000 -3.55470800 -0.66957000
H   -9.51260600 0.45869900 1.13316200
H   -1.94587500 0.36398200 0.65117900

1-$PdCl_2$ Central arene perpendicular to Cl-Pd-Cl φ_1 = 43.3°
E = -4233.2743378 Hartrees
G = -4233.266047 Hartrees
E(TZVPP)= -4236.290614 Hartrees
Pd   0.00000000 0.00000000 0.00000000
N   -0.00823000 2.07660400 -0.00140800
C   -1.17852500 2.73067600 0.01505900
C   -1.23630600 4.11960100 -0.00577900
C   -0.05343900 4.84541300 -0.02270300
C   1.17020300 4.16021800 -0.02495000
C   1.14038800 2.75746000 -0.02480200
H   2.06012600 2.17207900 -0.05428200
C   2.41703800 4.86331100 -0.02355500
C   3.46596300 5.47439700 -0.00921500
C   4.70890900 6.18547700 0.02189000
C   5.92900000 5.46832200 0.06579200
C   7.14906400 6.18552300 0.10962000
C   7.13438200 7.58723000 0.11030000
C   5.92895300 8.28105800 0.06562100
C   4.72354600 7.58718500 0.02103800
H   3.77499400 8.12322800 -0.01272900
H   5.92893500 9.37129500 0.06555500
H   8.08291600 8.12330800 0.14402300
C   8.39202900 5.47448300 0.14092000
C   9.44096400 4.86341400 0.15524300
C   10.6878990 4.16049800 0.15644000
C   10.7179120 2.75774200 0.15629700
H   9.79826100 2.17223200 0.18589700
N   11.8666250 2.07704900 0.13279600
Pd   11.8587150 0.00044200 0.13144300
N   11.8669250 -2.07615400 0.13299000
C   13.0372160 -2.73023500 0.11660100
C   13.0949880 -4.11915900 0.13753000

C	11.9121170	-4.84496300	0.15446400
C	10.6884790	-4.15976200	0.15663700
C	10.7183020	-2.75700300	0.15639800
H	9.79856800	-2.17161500	0.18582400
C	9.44164100	-4.86285100	0.15527900
C	8.39273800	-5.47397600	0.14095500
C	7.14978600	-6.18504000	0.10965700
C	5.92970500	-5.46787000	0.06576500
C	5.92969100	-4.04321900	0.06590600
C	5.92966200	-2.82888100	0.06605000
C	5.92952800	-1.39851700	0.06600400
C	4.91472300	-0.69323400	-0.60312100
C	4.91458500	0.69346500	-0.60314100
C	5.92925100	1.39896900	0.06596400
C	5.92911100	2.82933300	0.06594900
C	5.92903100	4.04367100	0.06590100
C	6.94404900	0.69368500	0.73509400
C	6.94418600	-0.69301100	0.73511400
H	7.73495300	-1.22540400	1.26495400
H	7.73471700	1.22625000	1.26490900
H	4.12379800	1.22585900	-1.13295000
H	4.12403800	-1.22579900	-1.13290900
C	4.70963700	-6.18505600	0.02177000
C	4.72430900	-7.58676400	0.02089600
C	5.92973100	-8.28060600	0.06555600
C	7.13513900	-7.58674800	0.11031900
H	8.08368500	-8.12280200	0.14407700
H	5.92974200	-9.37084300	0.06546400
H	3.77577400	-8.12283000	-0.01296400
C	3.46667700	-5.47400700	-0.00950100
C	2.41773300	-4.86295300	-0.02384800
C	1.17079800	-4.16004000	-0.02522200
C	1.14078600	-2.75728600	-0.02494600
H	2.06044500	-2.17177400	-0.05431700
N	-0.00793000	-2.07659300	-0.00157400
C	-1.17813500	-2.73083200	0.01473300
C	-1.23571900	-4.11976300	-0.00624400
C	-0.05274900	-4.84540600	-0.02313900
H	-0.05884500	-5.93589900	-0.03062100
H	-2.20483900	-4.61680600	-0.00002800
H	-2.07354400	-2.11213100	0.07228900
H	11.9183610	-5.93545500	0.16190400
H	14.0641740	-4.61607100	0.13125000
H	13.9325380	-2.11141000	0.05902000
Cl	10.1744870	0.00039900	1.78231500
Cl	13.5943110	0.00049500	-1.44988700

C 13.0368250 2.73128800 0.11620600
C 13.0944110 4.12022200 0.13702900
C 11.9114440 4.84586500 0.15406600
H 11.9175400 5.93635900 0.16143900
H 14.0635300 4.61726400 0.13058900
H 13.9322240 2.11258100 0.05855200
H -0.05968900 5.93590600 -0.03006100
H -2.20549600 4.61650700 0.00057200
H -2.07384200 2.11184200 0.07263100
Cl 1.68438600 0.00018400 -1.65071100
Cl -1.73572100 -0.00018700 1.58119000

1-$PdCl_2$ Central arene parallel to Cl-Pd-Cl $\varphi_1 = 142.3°$
E = -4233.266047 Hartrees
G = -4233.274338 Hartrees
E(TZVPP)= -4236.28355946 Hartrees
Pd 0.00000000 0.00000000 0.00000000
N -0.02088800 -2.08899000 -0.01289100
C 1.11985700 -2.78373800 -0.03114800
C 1.13911400 -4.18579800 0.00570800
C -0.08801600 -4.86307300 0.02870300
C -1.26391200 -4.12582200 0.02415100
C -1.19560900 -2.73702900 0.01477000
H -2.08804700 -2.11361500 0.05761900
H -2.23783600 -4.61324700 0.03940300
H -0.10326700 -5.95329600 0.05034400
C 2.38616700 -4.88787900 0.02323900
C 3.44240900 -5.48546200 0.05121900
C 4.68844000 -6.19034000 0.08219900
C 5.90845000 -5.47149800 0.10831400
C 7.12844100 -6.19037100 0.13439200
C 7.11375600 -7.59214000 0.13318600
C 5.90841600 -8.28658800 0.10822500
C 4.70309200 -7.59210800 0.08331500
H 3.75373000 -8.12734400 0.06251700
H 5.90840100 -9.37681800 0.10819000
H 8.06310400 -8.12740000 0.15396800
C 8.37449000 -5.48553000 0.16549700
C 9.43074600 -4.88797200 0.19343600
C 10.6778160 -4.18591800 0.21077900
C 10.6971330 -2.78385800 0.24766000
N 11.8379090 -2.08916600 0.22925200
C 13.0126010 -2.73724800 0.20139000
C 13.0808460 -4.12604200 0.19198700
C 11.9049160 -4.86324100 0.18760200
H 11.9201180 -5.95346400 0.16595200

H	14.0547470	-4.61351100	0.17658000
H	13.9050520	-2.11386000	0.15839600
Pd	11.8171220	-0.00020900	0.21638400
N	11.8380860	2.08886000	0.22820300
C	13.0128000	2.73687100	0.19955800
C	13.0811250	4.12565800	0.18951400
C	11.9052430	4.86293500	0.18533800
C	10.6781180	4.18569100	0.20934500
C	10.6973670	2.78364300	0.24679100
H	9.77319700	2.21028800	0.32160700
C	9.43107000	4.88779300	0.19226800
C	8.37484400	5.48542100	0.16468400
C	7.12878200	6.19026000	0.13403900
C	5.90878100	5.47139000	0.10828700
C	5.90879500	4.04591300	0.10824500
C	5.90879000	2.83116300	0.10822800
C	5.90873200	1.40067100	0.10822400
C	4.78251400	0.69259000	0.55937400
C	4.78244600	-0.69256000	0.55942100
C	5.90858700	-1.40077800	0.10830500
C	5.90852600	-2.83127000	0.10838200
C	5.90847500	-4.04602100	0.10837900
C	7.03480000	-0.69269800	-0.34286300
C	7.03487500	0.69245400	-0.34289600
H	7.90973200	1.23851600	-0.69444800
H	7.90958100	-1.23888100	-0.69442000
H	3.90759300	-1.23861400	0.91099100
H	3.90771400	1.23876100	0.91089300
C	4.68876600	6.19023700	0.08257500
C	4.70342100	7.59200600	0.08376700
C	5.90875300	8.28648100	0.10835700
C	7.11409800	7.59202900	0.13291400
H	8.06345500	8.12728500	0.15342800
H	5.90874300	9.37671100	0.10837900
H	3.75405400	8.12724400	0.06327100
C	3.44272000	5.48537200	0.05185200
C	2.38646800	4.88779200	0.02420100
C	1.13935800	4.18580100	0.00704700
C	1.11999400	2.78376100	-0.03038600
H	2.04411900	2.21032500	-0.10513400
N	-0.02078600	2.08906500	-0.01187000
C	-1.19544800	2.73717500	0.01664800
C	-1.26365600	4.12597100	0.02667100
C	-0.08771600	4.86314900	0.03094600
H	-0.10288400	5.95336400	0.05305100
H	-2.23754500	4.61344700	0.04262400

H	-2.08791100	2.11380600	0.05965500
H	11.9205060	5.95314800	0.16321100
H	14.0550520	4.61305300	0.17346200
H	13.9052060	2.11342100	0.15647200
Cl	9.93693800	0.00012400	1.62272200
Cl	13.6629680	-0.00074400	-1.24719700
H	9.77297900	-2.21040100	0.32187000
H	2.04405300	-2.21033000	-0.10520000
Cl	-1.84614600	-0.00039600	1.46320700
Cl	1.88037400	0.00022400	-1.40610200

TS 1-$PdCl_2$ $\varphi_1 = 142.3°$ ifreq= 49.9 cm^{-1}
E = -4233.266047 Hartrees
G = -4233.274338 Hartrees
E(TZVPP)= -4236.28242575 Hartrees

Pd	0.00000000	0.00000000	0.00000000
N	0.00449100	2.09351600	-0.00520000
C	-1.14402400	2.77421000	0.04570800
C	-1.19071300	4.17452400	-0.02154300
C	0.02531500	4.86749900	-0.10705700
C	1.21167000	4.14721900	-0.13455800
C	1.16660500	2.75768900	-0.09573700
H	2.06625400	2.14696500	-0.16549600
H	2.17612800	4.64914600	-0.19984100
H	0.02429600	5.95706400	-0.15348400
C	-2.44874300	4.86050500	-0.01756500
C	-3.50848900	5.45349800	-0.04374200
C	-4.75144100	6.16598600	-0.09766200
C	-5.98002800	5.46055200	-0.10336200
C	-7.19428000	6.18641400	-0.18516300
C	-7.16561600	7.58636100	-0.24551200
C	-5.95332300	8.26962000	-0.22984700
C	-4.75445400	7.56664500	-0.15922400
H	-3.80103200	8.09503400	-0.15251100
H	-5.94260900	9.35877300	-0.27692700
H	-8.10856200	8.12977500	-0.30726700
C	-8.44521700	5.48728900	-0.21011300
C	-9.50196200	4.88930700	-0.23355400
C	-10.7522750	4.19013700	-0.22897200
C	-10.7826640	2.79020400	-0.31779200
N	-11.9225540	2.09555700	-0.26001300
C	-13.0912500	2.74635200	-0.15244300
C	-13.1523130	4.13452500	-0.09678800
C	-11.9745420	4.86856000	-0.12243200
H	-11.9847520	5.95722200	-0.05914600
H	-14.1221360	4.62399400	-0.01789800

H	-13.9839460	2.12602300	-0.08185200
Pd	-11.9040970	-0.00015400	-0.26090700
N	-11.9224730	-2.09594500	-0.25925100
C	-13.0910690	-2.74675400	-0.15062900
C	-13.1520350	-4.13490300	-0.09426400
C	-11.9742510	-4.86890100	-0.12029200
C	-10.7520870	-4.19047300	-0.22796700
C	-10.7825930	-2.79058400	-0.31746100
H	-9.86786300	-2.21873100	-0.46773900
C	-9.50175400	-4.88960800	-0.23297000
C	-8.44500200	-5.48759100	-0.20981500
C	-7.19404900	-6.18669900	-0.18514600
C	-5.97979600	-5.46082300	-0.10346200
C	-5.99835000	-4.03972600	-0.03156400
C	-6.02316800	-2.82722900	0.02915400
C	-6.03174800	-1.39980800	0.07150400
C	-7.24092000	-0.69245300	0.16350700
C	-7.24096400	0.69209100	0.16349400
C	-6.03183900	1.39952700	0.07150200
C	-6.02333700	2.82695000	0.02916600
C	-5.99856300	4.03945000	-0.03153400
C	-4.82153900	0.69152400	0.00238900
C	-4.82149400	-0.69173000	0.00237800
H	-3.87733500	-1.22857300	-0.04824300
H	-3.87741400	1.22842800	-0.04819600
H	-8.18450800	1.23033100	0.22186200
H	-8.18442400	-1.23076000	0.22191700
C	-4.75119600	-6.16623400	-0.09798200
C	-4.75419100	-7.56688700	-0.15965700
C	-5.95305700	-8.26987600	-0.23018400
C	-7.16536600	-7.58664000	-0.24562500
H	-8.10831000	-8.13006600	-0.30729600
H	-5.94233000	-9.35902500	-0.27736200
H	-3.80076000	-8.09526000	-0.15311300
C	-3.50825500	-5.45371600	-0.04418400
C	-2.44853400	-4.86067300	-0.01812000
C	-1.19053900	-4.17462800	-0.02229300
C	-1.14388000	-2.77433700	0.04547500
H	-2.05371500	-2.19009400	0.16898200
N	0.00458500	-2.09357000	-0.00568300
C	1.16668700	-2.75767300	-0.09692000
C	1.21179400	-4.14718800	-0.13625100
C	0.02548000	-4.86752600	-0.10855500
H	0.02448300	-5.95707300	-0.15539800
H	2.17624700	-4.64905100	-0.20209400
H	2.06629000	-2.14689700	-0.16682700

H -11.9843710 -5.95753200 -0.05644600
H -14.1217890 -4.62437300 -0.01451700
H -13.9837560 -2.12644200 -0.07977800
Cl -10.0953530 -0.00026300 -1.75787700
Cl -13.6722690 0.00022900 1.29271400
H -9.86780700 2.21832400 -0.46716500
H -2.05389100 2.18988800 0.16858400
Cl -1.78557300 -0.00012900 1.52599200
Cl 1.77577100 0.00030800 -1.54163300

1-I$^+$ φ_1 = 52.5°
E = -2732.026113 Hartrees
G = -2731.499945 Hartrees
E(TZVPP)= -2734.378757 Hartrees
N 0.00000000 0.00000000 0.00000000
C 1.15702700 0.65862900 0.09022000
C 1.22549400 2.04688600 -0.08156900
C 0.03035200 2.73242900 -0.35732800
C -1.16125800 2.02250600 -0.44954300
C -1.14424700 0.64543300 -0.26365700
H -2.05123400 0.04086500 -0.32332100
H -2.10349600 2.52623100 -0.66319100
H 0.04927000 3.81387300 -0.49659200
C 2.48269300 2.71519200 0.02997500
C 3.55609600 3.27221900 0.13017800
C 4.80467300 3.96280100 0.24487900
C 6.01644800 3.24102700 0.35288000
C 7.22822100 3.96280600 0.46086700
C 7.21578900 5.36544100 0.46039300
C 6.01644500 6.06119400 0.35282900
C 4.81710200 5.36543500 0.24529800
H 3.87189000 5.90155400 0.16000300
H 6.01644500 7.15118300 0.35280700
H 8.16099900 5.90156400 0.54567700
C 8.47679500 3.27223000 0.57563000
C 9.55019200 2.71520400 0.67589900
C 10.8074030 2.04692200 0.78745200
C 10.8758940 0.65866800 0.61565400
N 12.0329270 0.00005200 0.70590500
C 13.1771580 0.64550000 0.96959900
C 13.1941440 2.02257300 1.15549000
C 12.0025280 2.73248100 1.06324600
H 11.9835930 3.81392400 1.20251300
H 14.1363700 2.52630900 1.36916800
H 14.0841500 0.04094400 1.02929000

I	12.0462910	-2.25113300	0.37954300
N	12.0349760	-4.50153100	0.04235600
C	13.1790360	-5.14349700	-0.23029400
C	13.1950080	-6.51709800	-0.44057900
C	12.0020510	-7.22681700	-0.36624000
C	10.8071140	-6.54496900	-0.08046000
C	10.8772420	-5.16055900	0.11985900
H	9.97983600	-4.58110500	0.34826100
C	9.54850600	-7.21326400	0.01394200
C	8.47436600	-7.77057600	0.10464500
C	7.22627100	-8.46122000	0.22319700
C	6.01650600	-7.73925200	0.35289000
C	6.01649500	-6.30777400	0.35293200
C	6.01646600	-5.09264000	0.35295100
C	6.01646700	-3.65696400	0.35293800
C	5.38549000	-2.94421400	1.38782700
C	5.38594700	-1.55536700	1.38808900
C	6.01646300	-0.84267600	0.35292000
C	6.01644400	0.59369700	0.35290300
C	6.01644600	1.80873700	0.35289800
C	6.64697400	-1.55537900	-0.68224200
C	6.64744100	-2.94422400	-0.68196000
H	7.12698200	-3.49370200	-1.49292000
H	7.12589100	-1.00616000	-1.49371600
H	4.90701700	-1.00614000	2.19955000
H	4.90594200	-3.49368300	2.19878900
C	4.80675100	-8.46124100	0.48256800
C	4.81942600	-9.86380000	0.48343800
C	6.01652400	-10.5595680	0.35284200
C	7.21361400	-9.86377800	0.22227500
H	8.15725700	-10.3999520	0.12138200
H	6.01653200	-11.6495510	0.35281800
H	3.87578800	-10.3999880	0.58431000
C	3.55864900	-7.77061600	0.60114500
C	2.48451900	-7.21328600	0.69186200
C	1.22590600	-6.54500700	0.78632400
C	1.15575400	-5.16059800	0.58600900
H	2.05314400	-4.58113300	0.35757300
N	-0.00198800	-4.50158600	0.66354800
C	-1.14603000	-5.14356700	0.93623200
C	-1.16197700	-6.51717000	1.14651300
C	0.03098500	-7.22687200	1.07213600
H	0.05114300	-8.30534200	1.23266800
H	-2.10423300	-7.01819200	1.36637200
H	-2.05368400	-4.53893700	0.98407200
I	-0.01334100	-2.25118700	0.32635200

H 11.9819120 -8.30528500 -0.52678400
H 14.1372760 -7.01810700 -0.66041500
H 14.0866830 -4.53885400 -0.27810500
H 9.97760300 0.07566900 0.40055300
H 2.05533100 0.07564000 0.30529300

1-I+ φ_1 = 122.2°
E = -2732.026122 Hartrees
G = -2731.500055 Hartrees
E(TZVPP)= -2734.378777 Hartrees
N 0.00000000 0.00000000 0.00000000
C 1.16884000 0.64385800 -0.00676800
C 1.23456400 2.04282300 -0.02135700
C 0.02344700 2.75542800 -0.02626000
C -1.18056300 2.06096500 -0.01686500
C -1.15945500 0.67156000 -0.00434300
H -2.07541900 0.07772400 0.00198800
H -2.13534100 2.58592400 -0.01985300
H 0.04004000 3.84577900 -0.03717800
C 2.50406900 2.69685300 -0.03025700
C 3.58499000 3.24841000 -0.03507900
C 4.83982200 3.93707200 -0.03271700
C 6.05630600 3.21509500 -0.02106900
C 7.27280200 3.93704800 -0.00940100
C 7.26028100 5.33972000 -0.00870100
C 6.05633200 6.03560500 -0.02105000
C 4.85237000 5.33974300 -0.03340100
H 3.90334900 5.87581200 -0.04186200
H 6.05634400 7.12558700 -0.02104900
H 8.20931300 5.87576900 -0.00023000
C 8.52761900 3.24836000 -0.00702600
C 9.60851500 2.69675300 -0.01173400
C 10.8779940 2.04267100 -0.02058300
C 10.9436620 0.64370500 -0.03540700
N 12.1124780 -0.00019800 -0.04212300
C 13.2719590 0.67131400 -0.03750400
C 13.2931220 2.06071600 -0.02474300
C 12.0891380 2.75522600 -0.01539100
H 12.0725890 3.84557600 -0.00428500
H 14.2479200 2.58563400 -0.02153300
H 14.1879000 0.07744100 -0.04380300
I 12.1215870 -2.27496300 -0.04753100
N 12.1123780 -4.54973200 -0.04187800
C 13.2718270 -5.22129800 -0.03702400
C 13.2929230 -6.61070000 -0.02411300

C	12.0889060	-7.30515100	-0.01484700
C	10.8777950	-6.59253800	-0.02028000
C	10.9435310	-5.19357900	-0.03525200
H	10.0335140	-4.58945400	-0.04227500
C	9.60828500	-7.24656200	-0.01151900
C	8.52735500	-7.79810200	-0.00681800
C	7.27253300	-8.48678000	-0.00941100
C	6.05604600	-7.76481500	-0.02124900
C	6.05605200	-6.33301400	-0.02122700
C	6.05608500	-5.11795100	-0.02121600
C	6.05612400	-3.68196500	-0.02118500
C	6.69172700	-2.96932000	1.01091200
C	6.69177300	-1.58047300	1.01093200
C	6.05621800	-0.86775500	-0.02114400
C	6.05627400	0.56823100	-0.02113600
C	6.05629300	1.78329400	-0.02111100
C	5.42062200	-1.58040100	-1.05324400
C	5.42057600	-2.96924900	-1.05326500
H	4.93771600	-3.51857400	-1.86233800
H	4.93779700	-1.03101900	-1.86229900
H	7.17463600	-1.03114700	1.82000200
H	7.17456100	-3.51870300	1.81996100
C	4.83955600	-8.48677800	-0.03312300
C	4.85209000	-9.88944900	-0.03384800
C	6.05604300	-10.5853240	-0.02131500
C	7.25999800	-9.88945300	-0.00875100
H	8.20902100	-10.4255130	-0.00014800
H	6.05604300	-11.6753060	-0.02134600
H	3.90306300	-10.4255060	-0.04247500
C	3.58473300	-7.79810200	-0.03568600
C	2.50383300	-7.24650000	-0.03113600
C	1.23435600	-6.59241100	-0.02245800
C	1.16869600	-5.19344800	-0.00733700
H	2.07874600	-4.58937500	-0.00012700
N	-0.00011600	-4.54953500	-0.00079200
C	-1.15960100	-5.22103700	-0.00587100
C	-1.18077300	-6.61043600	-0.01893400
C	0.02320700	-7.30495400	-0.02812200
H	0.03975100	-8.39530300	-0.03946500
H	-2.13557500	-7.13534400	-0.02251300
H	-2.07553900	-4.62715600	0.00029700
I	-0.00920100	-2.27476800	0.00506200
H	12.0723010	-8.39549900	-0.00362200
H	14.2476970	-7.13566300	-0.02071400
H	14.1877970	-4.62746800	-0.04325000
H	10.0336160	0.03962300	-0.04223700

H 2.07886200 0.03973600 -0.00017500

TS-1-I$^+$ φ_1 = 122.2° ifreq= 49.9 cm^{-1}
E = -2732.026122 Hartrees
G = -2731.500055 Hartrees
E(TZVPP)= -2734.371684 Hartrees

N	0.00000000	0.00000000	0.00000000
C	-1.15374100	0.65088300	0.16087400
C	-1.28012300	2.01541500	-0.12720100
C	-0.13362700	2.69407900	-0.57494000
C	1.06113900	1.99843400	-0.72166600
C	1.09572400	0.63947400	-0.43073700
H	2.00490800	0.04561200	-0.54208600
H	1.96569900	2.49833100	-1.06686600
H	-0.19290700	3.75816800	-0.80627400
C	-2.54527400	2.66288300	0.02575000
C	-3.62658600	3.20309600	0.14009300
C	-4.88272100	3.87806100	0.27793100
C	-6.09016800	3.14332100	0.36516000
C	-7.30859400	3.84927500	0.51431200
C	-7.30615600	5.25076600	0.56528900
C	-6.11311600	5.95974500	0.47081900
C	-4.90811600	5.27918900	0.32913200
H	-3.96858500	5.82734300	0.25875100
H	-6.12214900	7.04896300	0.51020400
H	-8.25406200	5.77656100	0.67996600
C	-8.55095300	3.14487800	0.62327000
C	-9.61968900	2.57840500	0.72837800
C	-10.8669900	1.89345400	0.86031100
C	-10.9271420	0.51122800	0.64517800
N	-12.0652400	-0.17385900	0.76868400
C	-13.2064000	0.44412300	1.10251000
C	-13.2354060	1.81564400	1.32785000
C	-12.0604110	2.54930300	1.20789500
H	-12.0535810	3.62585500	1.38193500
H	-14.1751960	2.29701000	1.59688200
H	-14.1003020	-0.17664400	1.18737800
I	-12.0379660	-2.42303100	0.39712700
N	-11.9734210	-4.66832100	0.00012200
C	-13.0692550	-5.30785800	-0.43023800
C	-13.0347340	-6.66685200	-0.72101900
C	-11.8399290	-7.36247400	-0.57450800
C	-10.6933230	-6.68374900	-0.12714200
C	-10.8196310	-5.31917400	0.16076700
H	-9.96812000	-4.74668900	0.53102500
C	-9.42814700	-7.33120600	0.02564800

C	-8.34682000	-7.87140100	0.13992800
C	-7.09067000	-8.54634400	0.27772900
C	-5.88324500	-7.81157300	0.36498300
C	-5.89723100	-6.38569100	0.30415000
C	-5.91729600	-5.17194900	0.25042700
C	-5.95056100	-3.74258200	0.21213400
C	-7.17714800	-3.05746700	0.19849600
C	-7.21276500	-1.67262900	0.20131600
C	-6.02302600	-0.92566500	0.21218700
C	-6.05625500	0.50370100	0.25051700
C	-6.07625300	1.71744100	0.30428600
C	-4.79643900	-1.61078100	0.19879400
C	-4.76082400	-2.99561300	0.20156500
H	-3.80656600	-3.51923300	0.19875400
H	-3.87109900	-1.03726000	0.18898500
H	-8.16702400	-1.14900500	0.19831800
H	-8.10248100	-3.63099300	0.18845200
C	-4.66480200	-8.51749900	0.51414100
C	-4.66721100	-9.91899200	0.56508800
C	-5.86023000	-10.6280000	0.47058400
C	-7.06524400	-9.94747200	0.32889900
H	-8.00476300	-10.4956470	0.25850200
H	-5.85117000	-11.7172190	0.50994400
H	-3.71929300	-10.4447670	0.67976700
C	-3.42244700	-7.81310200	0.62316100
C	-2.35369800	-7.24666600	0.72833500
C	-1.10639600	-6.56173100	0.86037400
C	-1.04620900	-5.17951400	0.64519800
H	-1.94055200	-4.62155900	0.36702200
N	0.09186800	-4.49441600	0.76884300
C	1.23298100	-5.11238700	1.10284800
C	1.26195500	-6.48390200	1.32822900
C	0.08697900	-7.21756900	1.20813600
H	0.08012800	-8.29411500	1.38221300
H	2.20170800	-6.96525900	1.59740700
H	2.12687100	-4.49161700	1.18782600
I	0.06460500	-2.24526100	0.39716500
H	-11.7807060	-8.42659300	-0.80571500
H	-13.9393820	-7.16679700	-1.06592300
H	-13.9784790	-4.71402200	-0.54140000
H	-10.0327590	-0.04674100	0.36714700
H	-2.00515000	0.07845800	0.53146100

Internal TS-1-I^+ $\varphi_1 = 95.9°$ ifreq= 20.0 cm^{-1}
E = -2732.026122 Hartrees
G = -2731.500055 Hartrees

E(TZVPP)= -2734.37865289 Hartrees

N	0.00000000	0.00000000	0.00000000
C	1.15642800	-0.66037700	0.08505400
C	1.22462300	-2.04611000	-0.10675500
C	0.02995800	-2.72729500	-0.39479600
C	-1.16127600	-2.01574800	-0.47928200
C	-1.14389100	-0.64131800	-0.27506300
H	-2.05032900	-0.03541200	-0.32932500
H	-2.10330800	-2.51609500	-0.70159200
H	0.04886800	-3.80667100	-0.54927100
C	2.48141800	-2.71626600	-0.00164800
C	3.55471100	-3.27378200	0.09652500
C	4.80266300	-3.96460100	0.21744100
C	6.01268300	-3.24293400	0.34167300
C	7.22270300	-3.96460100	0.46590500
C	7.21033600	-5.36730900	0.46619000
C	6.01268200	-6.06306200	0.34167200
C	4.81502900	-5.36730900	0.21715500
H	3.87091700	-5.90334700	0.11996500
H	6.01268200	-7.15305500	0.34167100
H	8.15444800	-5.90334800	0.56338000
C	8.47065400	-3.27378300	0.58682200
C	9.54394700	-2.71626700	0.68499500
C	10.8007420	-2.04610900	0.79010400
C	10.8689360	-0.66037600	0.59829700
N	12.0253630	0.00000100	0.68335200
C	13.1692550	-0.64131700	0.95841500
C	13.1866410	-2.01574600	1.16263300
C	11.9954070	-2.72729400	1.07814500
H	11.9764980	-3.80667100	1.23261800
H	14.1286730	-2.51609400	1.38494200
H	14.0756920	-0.03541000	1.01267800
I	12.0373370	2.24936000	0.34167000
N	12.0253640	4.49871900	-0.00000700
C	13.1692570	5.14003900	-0.27506100
C	13.1866430	6.51447000	-0.47926900
C	11.9954080	7.22601600	-0.39478200
C	10.8007420	6.54482900	-0.10675200
C	10.8689370	5.15909500	0.08504700
H	9.97106400	4.58004100	0.31246000
C	9.54394600	7.21498500	-0.00164500
C	8.47065200	7.77250000	0.09652600
C	7.22270000	8.46331800	0.21744000
C	6.01268000	7.74165000	0.34167100
C	6.01268200	6.30845300	0.34167300

C	6.01268400	5.09352600	0.34167300
C	6.01268700	3.65587500	0.34167300
C	6.01303800	2.94390100	-0.87065100
C	6.01213600	1.55481400	-0.87065100
C	6.01268700	0.84284000	0.34167300
C	6.01268500	-0.59481000	0.34167300
C	6.01268400	-1.80973800	0.34167300
C	6.01323800	1.55481400	1.55399700
C	6.01233500	2.94390100	1.55399700
H	6.01214100	3.49259000	2.49637400
H	6.01359500	1.00612500	2.49637400
H	6.01177800	1.00612500	-1.81302800
H	6.01323200	3.49259000	-1.81302800
C	4.80266000	8.46331700	0.46590000
C	4.81502500	9.86602400	0.46618300
C	6.01267900	10.5617780	0.34166700
C	7.21033300	9.86602600	0.21715300
H	8.15444500	10.4020640	0.11996300
H	6.01267800	11.6517720	0.34166500
H	3.87091300	10.4020630	0.56337100
C	3.55470800	7.77249900	0.58681800
C	2.48141400	7.21498400	0.68499200
C	1.22462000	6.54482700	0.79010400
C	1.15642500	5.15909400	0.59830400
H	2.05429600	4.58004000	0.37088400
N	-0.00000200	4.49871700	0.68336200
C	-1.14389400	5.14003600	0.95842400
C	-1.16127900	6.51446700	1.16263400
C	0.02995400	7.22601400	1.07814200
H	0.04886400	8.30539100	1.23261000
H	-2.10331100	7.01481500	1.38494100
H	-2.05033100	4.53413000	1.01268900
I	-0.01197500	2.24935900	0.34168400
H	11.9764980	8.30539400	-0.54924800
H	14.1286760	7.01482000	-0.70157100
H	14.0756950	4.53413400	-0.32932300
H	9.97106400	-0.08132400	0.37087600
H	2.05430000	-0.08132500	0.31247500

2- Structures

2-Ag$^+$ $\varphi_1 = 20.2°$
E = -2659.48092105 Hartrees
G = -2658.898033 Hartrees
E(TZVPP)= -2662.14170565 Hartrees

C	0.00000000	0.00000000	0.00000000
H	-1.02086300	-0.35348600	0.18989500
H	0.28535700	0.72997200	0.77268700
H	0.68751600	-0.85764300	0.02279700
O	-0.00805900	0.61752000	-1.28463700
C	1.20036700	1.08111000	-1.71666500
C	1.43764200	2.44992700	-1.70545900
C	2.64568000	2.99216200	-2.17354600
C	2.84511000	4.40847300	-2.15546800
C	2.95494700	5.61903700	-2.15157100
C	3.07984900	7.04294000	-2.15878100
C	4.35521400	7.65335100	-2.09859000
C	5.53332600	6.84618500	-2.01609500
C	6.53682400	6.16946800	-1.93197500
C	7.69871100	5.34446100	-1.82724700
C	7.55970000	3.95188100	-1.91420700
N	8.60653800	3.12346500	-1.83180900
C	9.83641500	3.63173900	-1.64902600
C	10.0636610	4.99933100	-1.54694000
C	8.98524600	5.87114900	-1.63969500
H	9.12624400	6.95019200	-1.56748400
H	11.0774910	5.36960400	-1.39835800
H	10.6635310	2.92281600	-1.58284800
Ag	8.28304000	0.97711000	-2.06230300
N	8.05429800	-1.18293000	-2.34599200
C	6.84827700	-1.75024300	-2.24552100
C	6.63063200	-3.11447200	-2.48123300
C	5.31078100	-3.65434000	-2.38590600
C	4.18743800	-4.10887600	-2.31884800
C	2.87693800	-4.67979300	-2.24165200
C	1.73271500	-3.84822800	-2.18939500
C	1.86721100	-2.42570300	-2.20950000
C	1.98881800	-1.21625500	-2.21191100
C	2.19306000	0.19902200	-2.19540000
C	3.40136800	0.74100100	-2.66346200
C	3.63916400	2.10981600	-2.65051900

O	4.85008200	2.57282300	-3.07783500
C	4.84792600	3.19719000	-4.35931400
H	4.15121600	4.04724000	-4.38424900
H	5.86664900	3.56269800	-4.53674500
H	4.57745300	2.46852400	-5.13850000
H	4.16861800	0.07897900	-3.06487500
C	0.45420800	-4.44998600	-2.11267600
C	0.33633400	-5.84687300	-2.08496800
C	1.46941400	-6.65208900	-2.13607400
C	2.73235800	-6.07423600	-2.21575600
H	3.62509100	-6.69822200	-2.25639200
H	1.36767100	-7.73717100	-2.11490200
H	-0.65708200	-6.29144600	-2.02626300
C	-0.72285200	-3.63748400	-2.07974400
C	-1.73176600	-2.96332800	-2.07363300
C	-2.90526000	-2.14815000	-2.08959800
C	-4.19853600	-2.68595700	-2.00983900
C	-5.28956800	-1.82553400	-2.03830400
C	-5.06720300	-0.45717500	-2.14132900
N	-3.83075000	0.06234500	-2.21542800
C	-2.77294400	-0.75603100	-2.19417100
H	-1.77705400	-0.30729500	-2.24953900
Ag	-3.50754800	2.21757800	-2.35016200
N	-3.26815200	4.39300900	-2.44497000
C	-4.34210400	5.19873300	-2.47142900
C	-4.22714700	6.58453700	-2.47159400
C	-2.96189400	7.15685000	-2.43494600
C	-1.83399700	6.32125600	-2.40617600
C	-2.04714500	4.93625900	-2.41989400
H	-1.19160800	4.25877800	-2.41187900
C	-0.50416100	6.84211400	-2.35350500
C	0.62197300	7.29122700	-2.29516600
C	1.93112400	7.86668300	-2.22880300
C	2.06891400	9.26230700	-2.23743800
C	3.32893700	9.84862600	-2.17835200
C	4.46635900	9.05088500	-2.10917900
H	5.45749500	9.50175900	-2.06085300
H	3.42493100	10.9344150	-2.18586500
H	1.17289900	9.88046300	-2.29225100
H	-2.83440900	8.23992200	-2.42729000
H	-5.12472800	7.20166800	-2.49607700
H	-5.32290500	4.72066700	-2.49310200
H	-5.90396900	0.24309300	-2.16448100
H	-6.30920000	-2.20506800	-1.98060800
H	-4.33495500	-3.76493700	-1.92840800
C	7.74031000	-3.90561500	-2.81811100

C 8.99223700 -3.31046100 -2.90959200
C 9.11054900 -1.94544900 -2.67179600
H 10.0796500 -1.44887100 -2.74428800
H 9.87605700 -3.89290800 -3.16791300
H 7.60909700 -4.97192000 -3.00558600
H 6.00941000 -1.11100900 -1.96398000
H 6.56919400 3.51135300 -2.06107200
H 0.67111700 3.11191000 -1.30262200

2-$PdCl_2$ Central arene parallel to Cl-Pd-Cl $\varphi_1 = 33.0°$
E = -4462.06462 Hartrees
G = -4461.474022 Hartrees
E(TZVPP)= -4465.344427 Hartrees
C 0.00000000 0.00000000 0.00000000
H 0.53525900 0.49560200 0.82649700
H -0.71052200 -0.72659100 0.41209700
H -0.55428500 0.75170600 -0.58507200
O 0.88989400 -0.72171700 -0.81642100
C 1.90888300 -0.04910300 -1.39073200
C 2.03277900 1.33748800 -1.38182200
C 3.12138000 1.97207400 -1.99899800
C 3.19589100 3.39911500 -2.00464700
C 3.20811900 4.61450000 -2.00368000
C 3.22757700 6.04110500 -2.00980200
C 2.02083500 6.78214500 -2.02897900
C 0.75983500 6.10588500 -2.04952300
C -0.31741800 5.54753800 -2.07562600
C -1.58681300 4.88967900 -2.10817800
C -1.65000000 3.48913800 -2.14092400
N -2.81284200 2.83186300 -2.15505700
C -3.96595300 3.51859900 -2.15887100
C -3.98942800 4.90880700 -2.14873500
C -2.79076200 5.60748100 -2.11590700
H -2.77047300 6.69767600 -2.09778800
H -4.94723200 5.42735900 -2.15844900
H -4.87934100 2.92509400 -2.14285100
Pd -2.86535700 0.74000200 -2.17287400
Cl -4.79059800 0.77329600 -0.81302100
Cl -0.87797000 0.68276000 -3.43204800
N -2.96210400 -1.33448900 -2.23410600
C -1.85425900 -2.07850800 -2.16824200
C -1.90653000 -3.48049400 -2.20520300
C -0.69601700 -4.23647400 -2.12544600
C 0.33955100 -4.86586000 -2.07079800
C 1.57108500 -5.59135200 -2.02848100
C 2.80037400 -4.88749500 -2.01476600

C	2.81989900	-3.46088200	-2.02160100
C	2.83210300	-2.24549600	-2.02091900
C	2.90653700	-0.81844300	-2.02669600
C	3.99517900	-0.18384200	-2.64381100
C	4.11906400	1.20273600	-2.63490200
O	5.13806300	1.87536400	-3.20917900
C	6.02770000	1.15362200	-4.02585000
H	6.58194300	0.40171400	-3.44099000
H	5.49222400	0.65825300	-4.85234600
H	6.73826900	1.88016900	-4.43795000
H	4.74481700	-0.80983500	-3.12339800
C	4.00701500	-5.62865700	-1.99399400
C	3.97107600	-7.03029200	-1.98561700
C	2.75611700	-7.70732800	-1.99550300
C	1.56272400	-6.99298700	-2.01701500
H	0.60405600	-7.51171000	-2.02818500
H	2.73937400	-8.79742300	-1.98717500
H	4.91292700	-7.57881100	-1.96832700
C	5.26804800	-4.95248700	-1.97299300
C	6.34526800	-4.39409200	-1.94655400
C	7.61463100	-3.73621100	-1.91368300
C	8.81857000	-4.45400400	-1.90413200
C	10.0172190	-3.75530600	-1.87106500
C	9.99373300	-2.36509000	-1.86253300
N	8.84062100	-1.67837100	-1.86813600
C	7.67779100	-2.33563800	-1.88247800
H	6.76840200	-1.73444000	-1.83562100
Pd	8.89319300	0.41339700	-1.85296000
Cl	10.8180020	0.37774000	-3.21338900
Cl	6.90584100	0.47184000	-0.59376700
N	8.99016700	2.48814900	-1.79447900
C	10.1901280	3.07801900	-1.67747700
C	10.3226390	4.46016800	-1.61837500
C	9.18564200	5.25257200	-1.69807000
C	7.93464400	4.63421700	-1.82411600
C	7.88227700	3.23219500	-1.85944300
H	6.92364000	2.71176400	-1.92436300
C	6.72402700	5.39014300	-1.90258600
C	5.68842200	6.01957900	-1.95591400
C	4.45681900	6.74504300	-1.99662900
C	4.46502500	8.14668700	-2.00693400
C	3.27154100	8.86092900	-2.02681300
C	2.05663200	8.18379100	-2.03622900
H	1.11471700	8.73223600	-2.05226000
H	3.28817900	9.95100800	-2.03423400
H	5.42364800	8.66550100	-1.99615600

H 9.24972400 6.34065500 -1.66225400
H 11.3143330 4.89920800 -1.51806900
H 11.0532240 2.41366300 -1.65878700
H 10.9071320 -1.77160400 -1.87845100
H 10.9750100 -4.27385200 -1.85993200
H 8.79828500 -5.54421900 -1.92103100
C -3.15730000 -4.09874300 -2.33398600
C -4.29421900 -3.30631300 -2.41456600
C -4.16186400 -1.92423300 -2.35361900
H -5.02494400 -1.25986400 -2.37280500
H -5.28572500 -3.74529800 -2.51694900
H -3.22127800 -5.18678600 -2.37114600
H -0.89573400 -1.55810500 -2.10134900
H -0.74064200 2.88800500 -2.18923400
H 1.28317600 1.96348900 -0.90219800

2-I^+ φ_1 = 23.1°
E = -2960.81532804 Hartrees
G = -2960.22834 Hartrees
E(TZVPP)= -2963.43351566 Hartrees
C 0.00000000 0.00000000 0.00000000
H -0.60187200 0.80932900 -0.42869900
H -0.67333600 -0.79068500 0.36883700
H 0.65838300 -0.41573100 -0.77828900
O 0.75882000 0.57200200 1.04485800
C 1.75198000 -0.16475900 1.58869000
C 1.78574400 -1.55649000 1.54439100
C 2.83956200 -2.27493500 2.13675600
C 2.84754000 -3.70555800 2.11631500
C 2.83757700 -4.92083700 2.14065800
C 2.85039000 -6.34919100 2.17797700
C 4.07116800 -7.05008600 2.32421800
C 5.30668200 -6.33690400 2.43469800
C 6.37071300 -5.75914300 2.51563200
C 7.63444100 -5.09505800 2.56984700
C 7.69437500 -3.69761500 2.47984900
N 8.86663300 -3.05755700 2.49504900
C 10.0241410 -3.72376700 2.60727100
C 10.0446660 -5.10844100 2.71395100
C 8.84108500 -5.80368600 2.69216800
H 8.82258500 -6.89165700 2.76506200
H 10.9972150 -5.62911700 2.80691400
H 10.9391160 -3.12829900 2.60887500
I 8.89295500 -0.80582700 2.25517800
N 8.90251100 1.46239000 2.00066900
C 7.73505300 2.10618100 2.01825600

C	7.66113700	3.49908700	1.89880700
C	6.38834500	4.14387400	1.94956000
C	5.29866600	4.67389300	2.01460600
C	4.04026300	5.34915700	2.10067400
C	2.83365700	4.61951000	2.21928100
C	2.83097200	3.18933700	2.23728400
C	2.80037400	1.97429500	2.23924900
C	2.79896700	0.54341100	2.22726000
C	3.84942300	-0.18044900	2.80780300
C	3.88686300	-1.56999100	2.76049000
O	4.95700800	-2.23242300	3.28638600
C	4.75404200	-2.79755800	4.57570000
H	3.92268300	-3.51796200	4.56511800
H	5.67959600	-3.32177000	4.84381000
H	4.55368300	-2.00742100	5.31562400
H	4.66244400	0.34802500	3.30590800
C	1.61781200	5.33803000	2.31722200
C	1.61941000	6.73984300	2.28628600
C	2.81330100	7.44191400	2.16039900
C	4.01698500	6.75149200	2.07070400
H	4.95895000	7.29178200	1.97632600
H	2.80605200	8.53156900	2.13549600
H	0.67036300	7.27029800	2.36397700
C	0.37928900	4.63931000	2.46927200
C	-0.67824600	4.06459900	2.62009600
C	-1.91350100	3.37764300	2.81623800
C	-3.08825300	4.04537100	3.20071500
C	-4.25885100	3.31996000	3.38949200
C	-4.23970100	1.94559300	3.19033600
N	-3.11432000	1.31864800	2.81892700
C	-1.97466700	1.98984800	2.63164300
H	-1.08658800	1.42788200	2.32450800
I	-3.15617800	-0.91405900	2.47231600
N	-3.17448100	-3.16562300	2.07519600
C	-4.31931700	-3.78984300	1.76872400
C	-4.33877500	-5.15178700	1.49025600
C	-3.14754100	-5.86741700	1.52607800
C	-1.95130500	-5.20386300	1.84773800
C	-2.02031300	-3.83253500	2.12306600
H	-1.12287700	-3.27003700	2.39158300
C	-0.69073000	-5.87185900	1.90546900
C	0.39089200	-6.41839000	1.96962600
C	1.64943900	-7.09091400	2.07508300
C	1.68232800	-8.49247900	2.10071200
C	2.89079700	-9.16774500	2.23692800
C	4.07799100	-8.45226000	2.35224900

H	5.02912400	-8.97289500	2.46345900
H	2.90690400	-10.2574330	2.25709100
H	0.74596700	-9.04428400	2.01813300
H	-3.12971400	-6.93576900	1.30788800
H	-5.28224800	-5.63877800	1.24531400
H	-5.22521700	-3.18089200	1.74786400
H	-5.12920500	1.32739600	3.32521100
H	-5.18511200	3.80877700	3.68995900
H	-3.06918900	5.12549600	3.34998500
C	8.86512500	4.20708700	1.74609700
C	10.0691480	3.51239300	1.72287300
C	10.0554660	2.12884500	1.85685300
H	10.9722360	1.53619900	1.85133800
H	11.0188040	4.03354100	1.60548400
H	8.84392200	5.29305600	1.64893600
H	6.83007400	1.50575500	2.13026400
H	6.78195200	-3.09627500	2.40632600
H	1.00146100	-2.11620900	1.03706900

3- Structures

3-Ag^+ $\varphi_1 = 42.8°$
E = -2936.910552 Hartrees
G = -2936.398436 Hartrees
E(TZVPP)= -2939.574349 Hartrees

C	0.00000000	0.00000000	0.00000000
C	0.00583700	1.37797900	0.25796600
C	-1.18453700	1.96617500	0.70954300
C	-2.30690100	1.16483400	0.88709000
C	-2.21696700	-0.19617300	0.61797000
N	-1.08030000	-0.76503500	0.18208600
Ag	-0.95653700	-2.92673800	-0.22381900
N	-0.99430400	-5.05907400	-0.75755500
C	0.10002200	-5.81390900	-0.61676400
C	0.13882100	-7.16447100	-0.99279800
C	1.35705800	-7.89506100	-0.83053900
C	2.41592300	-8.46921400	-0.68586300
C	3.66937000	-9.13803100	-0.51099700
C	4.85873800	-8.38393400	-0.37059100
C	6.08592900	-9.06387400	-0.18706600
C	6.11102100	-10.4655400	-0.15641800
C	4.93594200	-11.1953740	-0.30479200
C	3.72190500	-10.5384730	-0.47957200
H	2.79699800	-11.1040630	-0.59210200
H	4.96607300	-12.2847740	-0.28088100
H	7.06430000	-10.9741810	-0.01327500
C	7.30365500	-8.33035200	-0.01441700
C	8.34687000	-7.73191100	0.15014800
C	9.57458100	-7.02691700	0.34718300
C	10.7628600	-7.68113400	0.71000400
C	11.9207170	-6.93374900	0.88546000
C	11.8733870	-5.55765500	0.69163800
N	10.7416390	-4.92434900	0.34206800
C	9.62133200	-5.63639600	0.17790200
H	8.71233500	-5.09894500	-0.09882300
Ag	10.7418120	-2.77005300	-0.04729800
N	10.7100560	-0.63774700	-0.54446700
C	11.8312720	-0.01839300	-0.94894400
C	11.8452950	1.32420300	-1.31044500
C	10.6620520	2.05024600	-1.25827000
C	9.48498300	1.41046200	-0.83804800
C	9.56795400	0.05613200	-0.48592400
H	8.67192000	-0.46591000	-0.14378700
C	8.23238100	2.09476600	-0.75909200
C	7.16811300	2.67342600	-0.68200100

C	5.92800100	3.37942700	-0.56165700
C	4.71206800	2.66928700	-0.41659900
C	3.50758200	3.39487100	-0.25414900
C	2.26715400	2.70093100	-0.08567600
C	1.21570800	2.11538400	0.06865400
C	3.53181500	4.79671200	-0.25445600
C	4.73192100	5.48270200	-0.41218700
C	5.92423000	4.78120500	-0.56222500
H	6.86897400	5.31274400	-0.67574900
H	4.73885900	6.57276400	-0.41333500
H	2.59533400	5.34048500	-0.13071500
C	4.70314500	1.24121400	-0.43674700
C	4.70323100	0.02631600	-0.46748100
C	4.71155600	-1.39777500	-0.49158400
C	3.69454500	-2.14596000	0.20515200
C	3.70755900	-3.59469900	0.19976900
C	4.74469900	-4.31746000	-0.49444800
C	5.68138900	-3.55859800	-1.16478400
C	5.66463900	-2.13299600	-1.16515900
H	6.42956400	-1.59823100	-1.72964500
H	6.46011700	-4.07463100	-1.72781800
C	4.77876500	-5.74210400	-0.46286900
C	4.81771300	-6.95566500	-0.41597100
N	2.69767200	-4.12260800	0.87417900
S	1.82266000	-2.88957100	1.47937500
N	2.67706500	-1.63917800	0.88366600
H	10.6357390	3.10405000	-1.53796400
H	12.7778170	1.78796000	-1.63059600
H	12.7448290	-0.61438900	-0.98449900
H	12.7678140	-4.94565900	0.82002100
H	12.8599520	-7.40751300	1.16925800
H	10.7647250	-8.76257500	0.85103600
C	-1.02723900	-7.73535800	-1.52240900
C	-2.16226400	-6.94413200	-1.65847900
C	-2.10753800	-5.61054600	-1.27037300
H	-2.98154300	-4.96499800	-1.37284700
H	-3.08736600	-7.35051200	-2.06603100
H	-1.03283800	-8.78388300	-1.82237300
H	0.99260300	-5.35577700	-0.18385400
H	-3.07991200	-0.84990200	0.75617000
H	-3.24927000	1.58488100	1.23723100
H	-1.21831900	3.03589500	0.91921800
H	0.91698200	-0.47778600	-0.34744200

3-Ag^+ $\varphi_1 = 137.2°$

E = -2936.910552 Hartrees
G = -2936.398436 Hartrees
E(TZVPP)= -2939.574349 Hartrees

C	0.00000000	0.00000000	0.00000000
C	-0.08306000	-1.35430400	-0.35220100
C	1.09395900	-1.99413100	-0.77250100
C	2.27724300	-1.26815300	-0.82466800
C	2.26331000	0.07442000	-0.46307900
N	1.14214000	0.69381600	-0.05853300
Ag	1.17410300	2.82604400	0.43892700
N	1.17405100	4.98028000	0.82855300
C	0.05381600	5.69240900	0.66425400
C	0.00710000	7.08291200	0.83369300
C	-1.22053700	7.78798400	0.63650700
C	-2.26370300	8.38648000	0.47183500
C	-3.48136100	9.12009600	0.29907200
C	-4.70860100	8.44025800	0.11551400
C	-5.89789200	9.19445600	-0.02499700
C	-5.84523800	10.5948930	0.00636200
C	-4.63115200	11.2516960	0.18117800
C	-3.45614600	10.5217620	0.32965100
H	-2.50283000	11.0303250	0.47281700
H	-4.60092500	12.3410970	0.20503800
H	-6.77008900	11.1605590	-0.10625000
C	-7.15137300	8.52572400	-0.19992600
C	-8.21024400	7.95160500	-0.34468600
C	-9.42844700	7.22099000	-0.50706200
C	-10.5943510	7.79170700	-1.03719700
C	-11.7293260	7.00042400	-1.17337700
C	-11.6746960	5.66695000	-0.78486700
N	-10.5616110	5.11565000	-0.27154000
C	-9.46734700	5.87054700	-0.13062200
H	-8.57489600	5.41255700	0.30271100
Ag	-10.5239750	2.98344400	0.26269000
N	-10.6479610	0.82179000	0.66875500
C	-11.7846580	0.25303100	1.10469400
C	-11.8747220	-1.10798500	1.37373100
C	-10.7524650	-1.90944400	1.19602600
C	-9.56206200	-1.32135200	0.74438700
C	-9.56776900	0.05664500	0.48652100
H	-8.65076500	0.53438800	0.13907300
C	-8.35228600	-2.05886800	0.55491900
C	-7.30089600	-2.64448900	0.40047900
C	-6.06051800	-3.33848800	0.23191500
C	-4.85601000	-2.61295100	0.06943000
C	-3.64011200	-3.32313000	-0.07570800

C -2.39998100 -2.61716700 -0.19608800
C -1.33568900 -2.03855100 -0.27321400
C -3.64393600 -4.72490900 -0.07631800
C -4.83626900 -5.42636400 0.07375500
C -6.03634200 -4.74033000 0.23156300
H -6.97283900 -5.28406900 0.35533100
H -4.82937200 -6.51642800 0.07257600
H -2.69921800 -5.25648100 -0.18990000
C -4.86487000 -1.18487900 0.04932400
C -4.86471100 0.03001900 0.01861600
C -4.85631700 1.45410700 -0.00546900
C -5.87322500 2.20233100 0.69137000
C -5.86016100 3.65106800 0.68599800
C -4.82304400 4.37379100 -0.00829400
C -3.88645000 3.61489200 -0.67872200
C -3.90326100 2.18929200 -0.67911900
H -3.13841100 1.65450300 -1.24368400
H -3.10775100 4.13089700 -1.24181900
C -4.78886500 5.79843300 0.02330000
C -4.74977300 7.01199000 0.07018100
N -6.86997400 4.17900300 1.36050100
S -7.74497300 2.94598600 1.96576500
N -6.89065600 1.69557000 1.36997200
H -10.7863510 -2.97917300 1.40563500
H -12.8171060 -1.52794700 1.72393100
H -12.6475140 0.90685300 1.24302000
H -12.5486550 5.02135400 -0.88743300
H -12.6543060 7.40666900 -1.58133900
H -10.5998680 8.84014100 -1.33748300
C 1.19534700 7.73702400 1.19681800
C 2.35313000 6.98955500 1.37240700
C 2.30576600 5.61348300 1.17841700
H 3.20013500 5.00142400 1.30690400
H 3.29233600 7.46323700 1.65643800
H 1.19724500 8.81844900 1.33797500
H -0.85515100 5.15504000 0.38727400
H 3.17690100 0.67036500 -0.49862300
H 3.20972800 -1.73194800 -1.14487000
H 1.06757600 -3.04791700 -1.05225600
H -0.89599400 0.52207400 0.34219600

3-$PdCl_2$ Central arene parallel to Cl-Pd-Cl $\varphi_1 = 37.2°$
E = -4739.49390696 Hartrees
G = -4738.973786 Hartrees
E(TZVPP)= -4742.778098 Hartrees
Pd 0.00000000 0.00000000 0.00000000

N	-0.03443500	2.08931400	0.03829300
C	1.09817800	2.79705000	0.02551600
C	1.10107900	4.19901600	0.05659100
C	-0.13163600	4.86281100	0.12186500
C	-1.29832800	4.11156800	0.14810800
C	-1.21547200	2.72452100	0.09776000
H	-2.10268200	2.09261000	0.07867200
H	-2.27690900	4.58687100	0.20108600
H	-0.15830900	5.95255700	0.15332900
C	2.34146700	4.91106100	0.03018300
C	3.39617700	5.51113100	0.01017400
C	4.63680200	6.22450500	-0.01244900
C	5.86307800	5.51703100	-0.02424000
C	7.07584400	6.24782800	-0.05438900
C	7.04801600	7.64921000	-0.06019300
C	5.83615800	8.33223600	-0.04144100
C	4.63756700	7.62645500	-0.02058500
H	3.68277400	8.15213400	-0.00917300
H	5.82564000	9.42264200	-0.04606000
H	7.99256900	8.19270600	-0.08316300
C	8.32319400	5.54958100	-0.09950800
C	9.36922400	4.93801600	-0.16051300
C	10.5952200	4.20750100	-0.24614400
C	10.5741760	2.80454100	-0.22162200
N	11.6959330	2.08400600	-0.28991100
C	12.8831260	2.70069300	-0.40120400
C	12.9861900	4.08572500	-0.45077700
C	11.8325050	4.85339000	-0.36668200
H	11.8737050	5.94284400	-0.39376000
H	13.9683590	4.54662900	-0.54656900
H	13.7602030	2.05492900	-0.42284000
Pd	11.6486550	0.00092200	-0.24977100
N	11.6963140	-2.08242600	-0.28823900
C	12.8837160	-2.69884400	-0.39867900
C	12.9872570	-4.08385500	-0.44767400
C	11.8337600	-4.85183500	-0.36419200
C	10.5962130	-4.20625300	-0.24480400
C	10.5746740	-2.80328900	-0.22044300
H	9.63095900	-2.26031700	-0.15460400
C	9.37023000	-4.93740700	-0.16035500
C	8.32486700	-5.55005200	-0.09989100
C	7.07754200	-6.24829400	-0.05538000
C	5.86495100	-5.51716600	-0.02543000
C	4.63844900	-6.22411600	-0.01357800
C	4.63880900	-7.62607700	-0.02159500
C	5.83716600	-8.33224800	-0.04242400

C	7.04929700	-7.64964800	-0.06109600
H	7.99365700	-8.19348900	-0.08387200
H	5.82627200	-9.42249200	-0.04700300
H	3.68385300	-8.15146100	-0.01012500
C	3.39805800	-5.51036100	0.00930300
C	2.34337700	-4.91026200	0.02967200
C	1.10280300	-4.19855300	0.05655700
C	1.09941000	-2.79660100	0.02533700
H	2.03289400	-2.23396300	0.02093800
N	-0.03341500	-2.08923200	0.03861100
C	-1.21422200	-2.72482100	0.09872400
C	-1.29659400	-4.11189000	0.14928900
C	-0.12966300	-4.86274600	0.12255400
H	-0.15595200	-5.95249700	0.15418200
H	-2.27499000	-4.58751600	0.20280600
H	-2.10164200	-2.09320200	0.07994500
C	5.88510700	-4.09052000	-0.00842000
C	5.91248600	-2.87606700	0.01319200
C	5.92152100	-1.45034600	0.02349500
C	6.97826700	-0.72200800	0.67679500
C	6.97821400	0.72210600	0.67667000
C	5.92125900	1.45016600	0.02338100
C	4.92413700	0.71080500	-0.57474700
C	4.92421100	-0.71128300	-0.57464300
H	4.10546100	-1.23244600	-1.07005400
H	4.10524000	1.23174800	-1.07015000
C	5.91169100	2.87590000	0.01270900
C	5.88272100	4.09035500	-0.00698000
N	8.00373000	1.24085900	1.33755900
S	8.88947000	0.00017800	1.89309700
N	8.00381500	-1.24062200	1.33775000
H	11.8752780	-5.94128300	-0.39096700
H	13.9696390	-4.54446800	-0.54265400
H	13.7606370	-2.05286000	-0.42020000
Cl	13.5783130	0.00211300	1.10016500
Cl	9.65384600	0.00048200	-1.50832200
H	9.63067300	2.26129500	-0.15527700
H	2.03186900	2.23472300	0.02165200
Cl	-1.94276200	-0.00098500	-1.33141500
Cl	2.00294700	0.00044900	1.23927200

3-$PdCl_2$ Central arene parallel to Cl-Pd-Cl $\varphi_1 = 120.9°$
E = -4739.49390696 Hartrees
G = -4738.973786 Hartrees
E(TZVPP)= -4742.77268 Hartrees

Pd	0.00000000	0.00000000	0.00000000
N	-0.02761300	2.08434500	0.04207200
C	1.10429200	2.78635300	0.14752600
C	1.10242300	4.18800200	0.20992500
C	-0.13144000	4.85232100	0.18015400
C	-1.29662200	4.10504900	0.07955000
C	-1.20895100	2.71974600	0.00359500
H	-2.08951100	2.08882700	-0.11499800
H	-2.27542000	4.58203900	0.05201400
H	-0.15980900	5.94115300	0.23434300
C	2.33718300	4.90570700	0.29425200
C	3.38469100	5.51612000	0.34515100
C	4.62649200	6.22795100	0.35948800
C	5.84820400	5.51429400	0.34900700
C	7.06480900	6.23560600	0.29816600
C	7.04780200	7.63722300	0.28694600
C	5.84040500	8.32769300	0.31916000
C	4.63712500	7.62999600	0.34958000
H	3.68605000	8.16243800	0.36032400
H	5.83688400	9.41793500	0.31259700
H	7.99541300	8.17472200	0.25130600
C	8.30666700	5.52573500	0.24484500
C	9.34869200	4.90733100	0.17878500
C	10.5798400	4.18469100	0.08924100
C	10.5806800	2.78152300	0.09149500
N	11.7138760	2.07895700	0.01579100
C	12.8926990	2.71271500	-0.07753600
C	12.9756220	4.09996600	-0.10374700
C	11.8103760	4.84833200	-0.01470000
H	11.8355220	5.93852900	-0.02450000
H	13.9511380	4.57735300	-0.18582700
H	13.7781900	2.07843900	-0.10347800
Pd	11.6854290	0.00004600	0.03139400
N	11.7138790	-2.07885800	0.01560500
C	12.8927280	-2.71260400	-0.07754700
C	12.9756620	-4.09985100	-0.10374700
C	11.8104050	-4.84822000	-0.01483700
C	10.5798520	-4.18458800	0.08898300
C	10.5806870	-2.78141500	0.09118400
H	9.64906300	-2.21510400	0.13619900
C	9.34871100	-4.90723300	0.17840100
C	8.30673400	-5.52572000	0.24469700
C	7.06487300	-6.23558400	0.29815000
C	5.84826100	-5.51428600	0.34902100
C	4.62655100	-6.22794000	0.35964100
C	4.63718900	-7.62998500	0.34983500

C	5.84047200	-8.32767200	0.31936300
C	7.04786700	-7.63720300	0.28700000
H	7.99547300	-8.17470700	0.25131600
H	5.83695800	-9.41792900	0.31287200
H	3.68611800	-8.16243300	0.36068600
C	3.38474900	-5.51611000	0.34532100
C	2.33723900	-4.90570400	0.29437900
C	1.10248100	-4.18800500	0.20996100
C	1.10435400	-2.78635400	0.14757600
H	2.03610900	-2.22236200	0.20880000
N	-0.02753800	-2.08434300	0.04202000
C	-1.20887300	-2.71974700	0.00340600
C	-1.29655000	-4.10505000	0.07934400
C	-0.13137800	-4.85232400	0.18006300
H	-0.15975200	-5.94115600	0.23423900
H	-2.27534600	-4.58203900	0.05170000
H	-2.08941700	-2.08882900	-0.11529800
C	5.85958300	-4.08668100	0.38694600
C	5.88669600	-2.87369300	0.43482700
C	5.91717600	-1.44627700	0.46299800
C	5.32428400	-0.72157200	1.55693600
C	5.32423100	0.72157800	1.55690100
C	5.91711700	1.44627800	0.46295400
C	6.51529700	0.71198700	-0.53780600
C	6.51531500	-0.71198400	-0.53778800
H	7.01139400	-1.22985100	-1.35862300
H	7.01139600	1.22984200	-1.35864000
C	5.88665300	2.87369200	0.43481100
C	5.85955400	4.08668500	0.38697500
N	4.78155700	1.24161700	2.64948000
S	4.31075000	0.00001700	3.57740400
N	4.78166800	-1.24159900	2.64955000
H	11.8355540	-5.93841600	-0.02462300
H	13.9511910	-4.57723700	-0.18569800
H	13.7782090	-2.07830800	-0.10334000
Cl	13.5967780	-0.00011500	1.40123500
Cl	9.75949300	0.00006800	-1.33614600
H	9.64905500	2.21522400	0.13665000
H	2.03605300	2.22235700	0.20865500
Cl	-1.77459800	-0.00007600	-1.54633800
Cl	1.78796700	0.00001400	1.52607400

$3\text{-}I^{+}$ $\varphi_1 = 45.2°$
E = -3238.24499414 Hartrees
G = -3237.73031 Hartrees
E(TZVPP)= -3240.86626 Hartrees

C	0.00000000	0.00000000	0.00000000
C	0.07278800	1.38617400	0.18487700
C	-1.12201000	2.07228600	0.46222300
C	-2.31593800	1.36493900	0.54212800
C	-2.30281800	-0.01024300	0.34127600
N	-1.15931300	-0.65649200	0.07653100
I	-1.18175900	-2.90210800	-0.28944500
N	-1.18092000	-5.14534200	-0.67894100
C	-0.03441300	-5.81996500	-0.57305900
C	0.03360600	-7.19642500	-0.82219200
C	1.28271200	-7.87844500	-0.70111100
C	2.34936900	-8.44589200	-0.58623000
C	3.58897500	-9.14385700	-0.42749700
C	4.80528800	-8.42867900	-0.31818700
C	6.00720700	-9.15193100	-0.12939800
C	5.97995700	-10.5521870	-0.05999600
C	4.77788400	-11.2423730	-0.17705100
C	3.58844900	-10.5443580	-0.35932900
H	2.64157200	-11.0772720	-0.44576600
H	4.76755100	-12.3310270	-0.12359900
H	6.91602400	-11.0916080	0.08437700
C	7.25530400	-8.46090800	-0.01962100
C	8.32242500	-7.88998000	0.06528500
C	9.57559000	-7.20888100	0.13697400
C	10.7785140	-7.87511100	0.42277400
C	11.9677530	-7.15563400	0.46058000
C	11.9390110	-5.78951000	0.21003500
N	10.7859770	-5.16192200	-0.06119000
C	9.63020800	-5.82850700	-0.09749800
H	8.71879200	-5.26461500	-0.30896500
I	10.7925070	-2.92508400	-0.48675600
N	10.7728640	-0.68963000	-0.89762300
C	11.8716580	-0.09516400	-1.38308700
C	11.8863300	1.26652400	-1.65726600
C	10.7349390	2.01257800	-1.43255700
C	9.58687900	1.37882600	-0.92977100
C	9.65672400	0.00482400	-0.66391200
H	8.79548700	-0.52851900	-0.25006300
C	8.36472200	2.08229400	-0.70299400
C	7.31194700	2.66001100	-0.53215500
C	6.06987200	3.34575800	-0.34584300
C	4.86136100	2.61342700	-0.27020900
C	3.64717600	3.31844800	-0.09668900
C	2.40321800	2.61627100	-0.00415200
C	1.33161100	2.05336300	0.08460300
C	3.65473100	4.71792900	-0.00361200

C	4.85018800	5.42460400	-0.08119600
C	6.05163300	4.74442400	-0.25191600
H	6.99310000	5.29013600	-0.31440300
H	4.84554300	6.51214400	-0.00793300
H	2.70928800	5.24320400	0.13150300
C	4.87559600	1.18665600	-0.36573700
C	4.90461500	-0.02567400	-0.44292500
C	4.92283900	-1.45180700	-0.49408900
C	5.89434500	-2.19663900	0.26508000
C	5.87267700	-3.64128500	0.26914500
C	4.88971100	-4.36387700	-0.49636700
C	3.99860800	-3.60950800	-1.23244900
C	4.01355100	-2.18565700	-1.22895600
H	3.27798700	-1.65198200	-1.83190600
H	3.25473700	-4.12535100	-1.84058500
C	4.85511500	-5.78887300	-0.45675800
C	4.82511400	-7.00213600	-0.39407600
N	6.81946300	-4.17102100	1.03515400
S	7.64583800	-2.94260800	1.69743700
N	6.86064200	-1.69215600	1.02442800
H	10.7106290	3.08185800	-1.64557600
H	12.7923500	1.72881500	-2.04783800
H	12.7442370	-0.72955600	-1.55028700
H	12.8436760	-5.17860200	0.22290000
H	12.9167380	-7.64406100	0.67971700
H	10.7686310	-8.94933000	0.61055700
C	-1.15403800	-7.85537500	-1.18310100
C	-2.33644600	-7.13110300	-1.28170500
C	-2.31755200	-5.76550800	-1.02364500
H	-3.21677200	-5.15021300	-1.09103200
H	-3.27284900	-7.61442900	-1.55887800
H	-1.13635900	-8.92739500	-1.38251200
H	0.85606700	-5.25962100	-0.27987300
H	-3.21227400	-0.61212200	0.38941800
H	-3.25755400	1.86939300	0.75688800
H	-1.10080300	3.15226900	0.61212300
H	0.89764000	-0.58387600	-0.21355000

3-I^{+} $\varphi_1 = 133.9°$
E = -3238.24499414 Hartrees
G = -3237.73031 Hartrees
E(TZVPP)= -3240.866261 Hartrees

C	0.00000000	0.00000000	0.00000000
C	0.06985000	1.37405000	-0.26563200

C	-1.07827500	2.00792700	-0.76810200
C	-2.22974200	1.26195500	-0.99270600
C	-2.21508000	-0.09977200	-0.71873900
N	-1.11621100	-0.69437200	-0.23359900
I	-1.13579600	-2.92990300	0.17689500
N	-1.12931600	-5.16681700	0.60197900
C	0.02651400	-5.83330100	0.56594100
C	0.08113800	-7.21374500	0.80001700
C	1.33435300	-7.89477600	0.72858200
C	2.40150200	-8.46568400	0.64390900
C	3.64961700	-9.15671300	0.53437900
C	4.85156500	-8.43349000	0.34569100
C	6.06788300	-9.14869800	0.23655000
C	6.06838000	-10.5491920	0.30481400
C	4.87891400	-11.2471760	0.48700800
C	3.67683800	-10.5569660	0.60387000
H	2.74074800	-11.0963710	0.74814900
H	4.88921900	-12.3358280	0.54052700
H	7.01525500	-11.0821300	0.21849700
C	7.30751300	-8.45076500	0.07786100
C	8.37418700	-7.88334400	-0.03697900
C	9.62330000	-7.20133500	-0.15804500
C	10.8109930	-7.86034900	-0.51868100
C	11.9933960	-7.13607800	-0.61733300
C	11.9744510	-5.77042200	-0.35959000
N	10.8377820	-5.15020400	-0.01511000
C	9.69127700	-5.82482600	0.09081400
H	8.80076900	-5.26444100	0.38384000
I	10.8385620	-2.90691500	0.37404300
N	10.8161760	-0.66125700	0.73972600
C	11.9597460	-0.01498200	1.00412200
C	11.9729250	1.36023000	1.20478000
C	10.7789900	2.06757900	1.12504100
C	9.58412000	1.38143700	0.84806200
C	9.65685400	-0.00476100	0.66337100
H	8.75916100	-0.58865800	0.45010500
C	8.32527500	2.04860700	0.74795500
C	7.25364200	2.61148500	0.65932500
C	6.00966500	3.31364500	0.56693800
C	4.79546900	2.60859900	0.39350800
C	3.58693900	3.34090600	0.31806300
C	2.34484900	2.65515300	0.13186700
C	1.29205700	2.07745400	-0.03892400
C	3.60515600	4.73956900	0.41206900
C	4.80660700	5.41976900	0.58266200
C	6.00209200	4.71311900	0.66006100

H	6.94754400	5.23841400	0.79502900
H	4.81122700	6.50730500	0.65598800
H	2.66366800	5.28526400	0.34977200
C	4.78125900	1.18183600	0.29790800
C	4.75227600	-0.03049200	0.22065000
C	4.73408500	-1.45662200	0.16947400
C	3.76257500	-2.20144300	0.92864200
C	3.78424200	-3.64608900	0.93271900
C	4.76722000	-4.36869500	0.16723900
C	5.65830600	-3.61433300	-0.56888200
C	5.64334900	-2.19048700	-0.56541600
H	6.37888600	-1.65681400	-1.16840100
H	6.40217000	-4.13018600	-1.17701700
C	4.80179700	-5.79369000	0.20692100
C	4.83176900	-7.00695100	0.26968200
N	2.83740700	-4.17581600	1.69868100
S	2.01107400	-2.94739100	2.36098000
N	2.79623800	-1.69694600	1.68793100
H	10.7578260	3.14758500	1.27478200
H	12.9145960	1.86470300	1.41925700
H	12.8692110	-0.61685700	1.05213900
H	12.8736590	-5.15511900	-0.42708000
H	12.9298380	-7.61944900	-0.89429700
H	10.7933550	-8.93241700	-0.71783200
C	-1.12185200	-7.88014400	1.08513300
C	-2.31116200	-7.16076500	1.12268300
C	-2.28242000	-5.79457000	0.87255400
H	-3.18712900	-5.18372600	0.88522800
H	-3.26019800	-7.64933700	1.34128300
H	-1.11197900	-8.95442100	1.27258400
H	0.93797700	-5.26928900	0.35499100
H	-3.08772300	-0.73409800	-0.88585000
H	-3.13581200	1.72434900	-1.38303900
H	-1.05397000	3.07723900	-0.98095800
H	0.86130800	-0.53343800	0.41357300

4- Structures

4-Ag^{+} $\varphi_1 = 34.7°$
E = -3044.454165 Hartrees
G = -3043.761324 Hartrees
E(TZVPP)= -3047.513848 Hartrees

N	0.00000000	0.00000000	0.00000000
C	-0.84670600	1.02969600	-0.04888700
C	-0.41799100	2.36136300	-0.12618600
C	0.96182100	2.61010600	-0.11572200
C	1.83682700	1.53206000	-0.04626300
C	1.31910800	0.24068300	-0.00181800
H	1.98210000	-0.62576700	0.03336100
H	2.91592900	1.68235900	-0.03680600
H	1.33241500	3.63441500	-0.17039800
C	-1.40785600	3.38097900	-0.27039700
C	-2.31122200	4.17485100	-0.43145000
C	-3.38456500	5.10433400	-0.60142200
C	-4.69918700	4.62891200	-0.81825200
C	-5.75253400	5.56566300	-0.95046200
C	-5.47838400	6.93952000	-0.87927500
C	-4.17916700	7.39297900	-0.67616900
C	-3.13719100	6.48250900	-0.53500600
H	-2.11736900	6.82819900	-0.36647400
H	-3.97846700	8.46306800	-0.62299700
H	-6.30028800	7.64748100	-0.98520400
C	-7.10661200	5.14197000	-1.15476100
C	-8.27625300	4.86169100	-1.32462100
C	-9.67483300	4.63895300	-1.53064000
C	-10.2209110	3.34727700	-1.55871500
N	-11.5257160	3.12139300	-1.74534900
C	-12.3541400	4.16271500	-1.91660500
C	-11.9039620	5.47843200	-1.90548500
C	-10.5514270	5.72224000	-1.70980600
H	-10.1604150	6.74020800	-1.69490300
H	-12.6101540	6.29545500	-2.04972000
H	-13.4111990	3.93699800	-2.06925600
Ag	-12.3507080	1.06044900	-1.77972100
N	-13.2167110	-0.94700800	-2.12736400
C	-14.5358450	-1.18753000	-2.12502000
C	-15.0537000	-2.47884300	-2.08027200
C	-14.1787980	-3.55698200	-2.01103400
C	-12.7989530	-3.30840000	-2.00108000
C	-12.3701040	-1.97679400	-2.07867900
H	-11.2989770	-1.75808600	-2.06616700
C	-11.8091460	-4.32809500	-1.85705700

C	-10.9057500	-5.12198300	-1.69624300
C	-9.83230200	-6.05139000	-1.52657400
C	-8.51770100	-5.57583700	-1.30990000
C	-8.22781800	-4.17852100	-1.22886700
C	-7.88401200	-3.01538700	-1.14993400
C	-7.28491100	-1.71743400	-1.10132500
C	-7.93668500	-0.56903300	-0.58522000
C	-7.24468900	0.64688600	-0.55545500
C	-5.93244300	0.77064400	-1.02657300
C	-5.33322800	2.06854100	-0.97798200
C	-4.98923900	3.23162300	-0.89913200
C	-5.28068800	-0.37775400	-1.54269200
C	-5.97266600	-1.59367100	-1.57243600
H	-5.48698400	-2.47536400	-1.99163600
C	-3.94041200	-0.33711700	-2.04139000
C	-2.81556900	-0.37104100	-2.50428800
C	-1.46994100	-0.40627400	-3.00088700
C	-0.84508600	-1.64031200	-3.26546800
C	0.49598800	-1.67614200	-3.64639800
C	1.21822300	-0.48869000	-3.77784400
C	0.59263100	0.73914200	-3.54774700
C	-0.74473500	0.78620800	-3.16534900
H	-1.24016300	1.74134700	-2.98214600
H	1.15074100	1.66778800	-3.67608200
H	2.26501200	-0.51910000	-4.08282100
H	0.97331600	-2.63331600	-3.86152800
H	-1.42609600	-2.56318000	-3.19457200
H	-7.73036500	1.52857300	-0.13623700
C	-9.27697900	-0.60963400	-0.08657300
C	-10.4018010	-0.57572700	0.37638500
C	-11.7473550	-0.54057200	0.87318000
C	-12.4723900	-1.73312100	1.03791900
C	-13.8096910	-1.68616000	1.42054700
C	-14.4353860	-0.45836800	1.65059200
C	-13.7133230	0.72914900	1.51884300
C	-12.3723150	0.69342300	1.13768200
H	-11.7914370	1.61635600	1.06653200
H	-14.1907320	1.68629700	1.73389900
H	-15.4821250	-0.42804200	1.95574600
H	-14.3676730	-2.61485300	1.54909500
H	-11.9768800	-2.68822600	0.85475900
C	-7.46423000	-6.51248000	-1.17792700
C	-7.73823700	-7.88636000	-1.24923300
C	-9.03743100	-8.33994800	-1.45220000
C	-10.0795330	-7.42958300	-1.59310200
H	-11.0993430	-7.77536900	-1.76151700

H -9.23802200 -9.41006500 -1.50543500
H -6.91623800 -8.59423900 -1.14349800
C -6.11016400 -6.08869300 -0.97371100
C -4.94051700 -5.80844000 -0.80385400
C -3.54192600 -5.58581200 -0.59776000
C -2.99574200 -4.29418700 -0.56950100
H -3.64100800 -3.42235300 -0.69958700
N -1.69094300 -4.06843000 -0.38266700
C -0.86262300 -5.10983200 -0.21140100
C -1.31290700 -6.42551400 -0.22272400
C -2.66543600 -6.66919000 -0.41860200
H -3.05652800 -7.68712700 -0.43365900
H -0.60681300 -7.24261900 -0.07848000
H 0.19443600 -4.88421300 -0.05860300
Ag -0.86575200 -2.00750900 -0.34817300
H -14.5494910 -4.58124300 -1.95613100
H -16.1328240 -2.62901300 -2.08931900
H -15.1987430 -0.32100200 -2.16003300
H -9.57555100 2.47551200 -1.42864800
H -1.91780300 0.81086600 -0.06180500

4-Ag^+ $\varphi_1 = 147.06°$
E = -3044.454165 Hartrees
G = -3043.761324 Hartrees
E(TZVPP)= -3047.513848 Hartrees
N 0.00000000 0.00000000 0.00000000
C -1.30477100 -0.22573600 -0.18708400
C -1.85111100 -1.51731800 -0.21447000
C -0.97481600 -2.60066100 -0.03414200
C 0.37767800 -2.35700500 0.16197700
C 0.82812400 -1.04137100 0.17238100
H 1.88516000 -0.81576900 0.32536200
H 1.08362100 -3.17407900 0.30712800
H -1.36604000 -3.61855700 -0.04848800
C -3.24965500 -1.73999800 -0.42085300
C -4.41924200 -2.02039400 -0.59091400
C -5.77321900 -2.44431600 -0.79545900
C -6.82674700 -1.50778900 -0.92780500
C -8.14123100 -1.98349400 -1.14485500
C -8.38830800 -3.36171300 -1.21136900
C -7.34616400 -4.27196400 -1.07004600
C -6.04707600 -3.81823100 -0.86670700
H -5.22504800 -4.52602600 -0.76063900
H -7.54662500 -5.34210000 -1.12325000
H -9.40802900 -3.70760400 -1.38010500
C -9.21471300 -1.05419000 -1.31492300

C	-10.1180910	-0.26034000	-1.47602500
C	-11.1078610	0.75934700	-1.62037600
C	-10.6790440	2.09103000	-1.69697600
N	-11.5256710	3.12078000	-1.74597100
C	-12.8447950	2.88015000	-1.74493800
C	-13.3625910	1.58878400	-1.70121800
C	-12.4876720	0.51067000	-1.63168000
H	-12.8583510	-0.51363800	-1.57757500
H	-14.4416950	1.43853200	-1.71130400
H	-13.5077260	3.74664400	-1.78017000
Ag	-10.6600860	5.12825800	-1.39741900
N	-9.83517800	7.18926900	-1.36299800
C	-10.6635620	8.23062700	-1.53417200
C	-10.2133540	9.54633700	-1.52289200
C	-8.86082000	9.79008300	-1.32715900
C	-7.98425200	8.70674800	-1.14811300
C	-8.53036100	7.41509200	-1.17630800
H	-7.88503400	6.54329400	-1.04627700
C	-6.58566300	8.92948500	-0.94211600
C	-5.41601600	9.20978400	-0.77231600
C	-4.06196000	9.63359400	-0.56810300
C	-3.00848000	8.69693500	-0.43630100
C	-3.29838600	7.29963400	-0.51742100
C	-3.64224600	6.13652200	-0.59634100
C	-4.24144700	4.83861900	-0.64481100
C	-3.58982100	3.69019000	-1.16101700
C	-4.28188900	2.47431600	-1.19069600
C	-5.59407900	2.35063200	-0.71940800
C	-6.19326500	1.05271300	-0.76794400
C	-6.53702200	-0.11043500	-0.84682900
C	-6.24570000	3.49906300	-0.20319000
C	-5.55363400	4.71492900	-0.17349200
H	-6.03918400	5.59663800	0.24582200
C	-7.58593700	3.45848400	0.29560600
C	-8.71076100	3.49234200	0.75855600
C	-10.0563920	3.52733800	1.25519100
C	-10.6814820	4.76124900	1.51977800
C	-12.0225890	4.79681700	1.90061000
C	-12.7446200	3.60923300	2.03193100
C	-12.1187910	2.38152300	1.80183700
C	-10.7813890	2.33471700	1.41955200
H	-10.2857870	1.37967800	1.23631600
H	-12.6767500	1.45277100	1.93005400
H	-13.7914470	3.63944500	2.33680000
H	-12.5001170	5.75389500	2.11572300
H	-10.1006380	5.68422900	1.44896500

H -3.79630300 1.59260600 -1.60996700
C -2.24958300 3.73065100 -1.65982100
C -1.12477100 3.69641800 -2.12277500
C 0.22084300 3.66091300 -2.61941200
C 0.94616800 4.85324800 -2.78437900
C 2.28350900 4.80588300 -3.16680100
C 2.90897200 3.57789100 -3.39640700
C 2.18662800 2.39057300 -3.26445800
C 0.84556300 2.42670200 -2.88351000
H 0.26444800 1.50392600 -2.81227200
H 2.66384500 1.43325800 -3.47920800
H 3.95575300 3.54725400 -3.70139200
H 2.84171800 5.73441400 -3.29553300
H 0.45084800 5.80850600 -2.60152300
C -1.69386600 9.17245000 -0.21962400
C -1.44664000 10.5506360 -0.15292900
C -2.48875000 11.4610200 -0.29366200
C -3.78795400 11.0074640 -0.49662900
H -4.60995700 11.7153610 -0.60222100
H -2.28816400 12.5311250 -0.24028700
H -0.42682600 10.8964090 0.01548700
C -0.62038200 8.24304500 -0.05009000
C 0.28310900 7.44923400 0.11058400
C 1.27309900 6.42968800 0.25447600
C 0.84459200 5.09797900 0.33209700
H -0.22648000 4.87899700 0.31957800
N 1.69146400 4.06839900 0.38089800
C 3.01052900 4.30926100 0.37860600
C 3.52806600 5.60070300 0.33375300
C 2.65289300 6.67861100 0.26441800
H 3.02332000 7.70296600 0.20946800
H 4.60715200 5.75114500 0.34283100
H 3.67364300 3.44290300 0.41376500
Ag 0.82566700 2.06082100 0.03311600
H -8.46978200 10.8080390 -1.31210700
H -10.9195170 10.3633970 -1.66704900
H -11.7206220 8.00494700 -1.68687800
H -9.60793900 2.30979600 -1.68340300
H -1.94988100 0.64608300 -0.31805700

4-$PdCl_2$ φ_1 =43.6°
E = -4847.018743 Hartrees
G = -4846.319907 Hartrees
E(TZVPP)= -4850.703032 Hartrees
Pd 0.00000000 0.00000000 0.00000000
Cl -1.57282200 -0.31303800 1.71108000

Cl	1.44705700	0.35781700	-1.82435100
N	0.35799200	-2.05188700	-0.12294100
C	1.57827700	-2.48927900	-0.44376200
C	1.86436800	-3.85335300	-0.59915100
C	3.19557800	-4.24986900	-0.94438900
C	4.34134000	-4.53047300	-1.23031300
C	5.71176200	-4.82426700	-1.52355300
C	6.66789500	-3.78085500	-1.49235000
C	6.28245200	-2.43353500	-1.22824800
C	6.03240300	-1.26392200	-1.02072100
C	5.84584200	0.12714100	-0.74917800
C	4.82646900	0.85140000	-1.37361000
C	4.67479000	2.22196500	-1.13587600
C	5.56916900	2.87037800	-0.24285800
C	5.38250700	4.26140600	0.02878000
C	5.13271700	5.43117300	0.23572300
C	4.74736500	6.77849400	0.50002100
C	3.38261500	7.08245000	0.72786400
C	2.38628000	6.05577800	0.64101000
C	1.52503100	5.20823500	0.51818100
C	0.44857900	4.28065800	0.33868500
C	0.67003200	2.89620100	0.25068300
N	-0.33924200	2.03487000	0.10407800
C	-1.60113400	2.47721900	0.01373700
C	-1.90030000	3.83314900	0.04497700
C	-0.86768300	4.74637700	0.21266100
H	-1.06223800	5.81881500	0.24821000
H	-2.93497800	4.15940900	-0.05069600
H	-2.37396000	1.71177100	-0.06024400
H	1.67916200	2.48160900	0.28303200
C	3.00474200	8.40340000	1.01007500
C	3.95682900	9.41698200	1.06030000
C	5.29701400	9.13232700	0.81661700
C	5.70359300	7.82181100	0.53149000
C	7.07397900	7.52796200	0.23830700
C	8.21973700	7.24730700	-0.04756600
C	9.55100200	6.85095700	-0.39269800
C	9.83735300	5.48691300	-0.54785300
N	11.0576880	5.04968100	-0.86874300
C	12.0523680	5.92805000	-1.06315500
C	11.8530550	7.29739100	-0.92102300
C	10.5921370	7.77004600	-0.58062500
H	10.4014090	8.83711800	-0.46116400
H	12.6875160	7.97888100	-1.08123200
H	13.0127410	5.50418800	-1.35844700
Pd	11.4156810	2.99774200	-0.99213400

Cl	12.9894400	3.31111100	-2.70234700
Cl	9.96777200	2.63931600	0.83143900
N	11.7547340	0.96284000	-1.09691100
C	13.0166290	0.52034400	-1.00723400
C	13.3156190	-0.83561400	-1.03883000
C	12.2828340	-1.74869100	-1.20625900
C	10.9665710	-1.28280800	-1.33169900
C	10.7453070	0.10164600	-1.24331400
H	9.73621000	0.51636000	-1.27507900
C	9.89000400	-2.21026900	-1.51101700
C	9.02888500	-3.05798200	-1.63365800
C	8.03265200	-4.08473300	-1.72022300
C	8.41063000	-5.40573000	-2.00208100
C	7.45865500	-6.41942400	-2.05196000
C	6.11845600	-6.13482900	-1.80829900
H	5.36882000	-6.92582600	-1.83045400
H	7.76520100	-7.44178900	-2.27465900
H	9.46377700	-5.62373900	-2.17988200
H	12.4772480	-2.82113900	-1.24209500
H	14.3503030	-1.16199400	-0.94366200
H	13.7895770	1.28569400	-0.93357700
H	9.06608600	4.73402900	-0.38423100
H	6.04672500	9.92324600	0.83902800
H	3.65038200	10.4392840	1.28326500
H	1.95160400	8.62145300	1.18789900
C	6.58855000	2.14612700	0.38156300
C	6.74022600	0.77555900	0.14383200
C	7.79436000	0.03855800	0.76802300
C	8.72112400	-0.58070200	1.25123100
C	9.88586500	-1.27140400	1.72282700
C	11.0255080	-0.53968800	2.10341600
C	12.1889270	-1.21117400	2.46883800
C	12.2313330	-2.60674400	2.45912400
C	11.0987930	-3.33551600	2.09092100
C	9.92897500	-2.67526600	1.72614100
H	9.04205100	-3.23392200	1.42359500
H	11.1267180	-4.42634900	2.08982100
H	13.1460890	-3.12826400	2.74604300
H	13.0712720	-0.63918600	2.76095300
H	10.9798350	0.55103000	2.08213000
H	7.28232200	2.64792500	1.05512200
C	3.62071600	2.95896200	-1.76017000
C	2.69408300	3.57828400	-2.24355300
C	1.52945800	4.26892500	-2.71550200
C	0.39000700	3.53712100	-3.09649800
C	-0.77329700	4.20851800	-3.46244200

C	-0.81578300	5.60408900	-3.45285200
C	0.31655900	6.33294500	-3.08421600
C	1.48627300	5.67278100	-2.71893600
H	2.37305200	6.23151100	-2.41611300
H	0.28858000	7.42377800	-3.08320900
H	-1.73044900	6.12553900	-3.74019000
H	-1.65549000	3.63646300	-3.75487700
H	0.43573200	2.44640700	-3.07512500
H	4.13265000	0.34960000	-2.04712100
C	0.82303500	-4.77226900	-0.41147900
C	-0.43781300	-4.29944500	-0.07103900
C	-0.63687200	-2.93009100	0.07129000
H	-1.59716900	-2.50607300	0.36661900
H	-1.27240600	-4.98081100	0.08900700
H	1.01355100	-5.83935600	-0.53115500
H	2.34967900	-1.73650600	-0.60730400

4-$PdCl_2$ $\varphi_1 = 145.3°$
E = -4847.024273 Hartrees
G = -4846.32565 Hartrees
E(TZVPP)= -4850.697291 Hartrees

Pd	0.00000000	0.00000000	0.00000000
Cl	-1.86615600	-0.35834900	-1.42329500
Cl	1.94093600	0.35113700	1.26543400
N	-0.61524100	1.96809500	0.30351000
C	0.28280100	2.95399700	0.34988400
C	-0.09930400	4.30380600	0.32914600
C	0.90293200	5.32239700	0.28737200
C	1.75699600	6.18249000	0.22331500
C	2.76382200	7.19608000	0.15418000
C	4.11417500	6.84036600	-0.07967200
C	4.48642400	5.47490200	-0.26346600
C	4.81338400	4.31537500	-0.41935300
C	5.20573400	2.94960300	-0.57925900
C	6.43059700	2.51498600	-0.06350300
C	6.83575100	1.18248100	-0.17698700
C	5.98571200	0.26000700	-0.84110400
C	6.37802400	-1.10577400	-1.00102200
C	6.70488400	-2.26532800	-1.15691400
C	7.07708300	-3.63078800	-1.34083700
C	6.09707100	-4.65201400	-1.29429500
C	4.72579100	-4.31443600	-1.06337300
C	3.56408100	-4.02330700	-0.86795100
C	2.19178600	-3.68507300	-0.64921800
C	1.81766400	-2.34806900	-0.44777800
N	0.54332800	-1.99261300	-0.26974300

C	-0.41798400	-2.92851900	-0.25779600
C	-0.12721400	-4.27755900	-0.42401400
C	1.18953600	-4.66469200	-0.62997800
H	1.45552700	-5.71206300	-0.77657400
H	-0.93563300	-5.00703700	-0.40048400
H	-1.43919600	-2.56896700	-0.13645700
H	2.56220600	-1.55201200	-0.40354100
C	6.47114800	-5.99086800	-1.47213700
C	7.80251000	-6.32792700	-1.69546200
C	8.77445200	-5.33443100	-1.74743000
C	8.42744200	-3.98653400	-1.57461400
C	9.43430900	-2.97296800	-1.64352300
C	10.2883640	-2.11286300	-1.70751600
C	11.2906090	-1.09427600	-1.74915300
C	10.9085030	0.25552700	-1.77011700
N	11.8065260	1.24143900	-1.72352800
C	13.1177400	0.95037500	-1.72036400
C	13.5769290	-0.36020200	-1.73404900
C	12.6563030	-1.40011200	-1.72217800
H	12.9774020	-2.44187400	-1.69103900
H	14.6490150	-0.55187800	-1.72547600
H	13.8008580	1.79648400	-1.66465300
Pd	11.1912020	3.20957200	-1.42029800
Cl	13.0570980	3.56811800	0.00328700
Cl	9.25045600	2.85836600	-2.68600300
N	10.6478670	5.20221300	-1.15086900
C	11.6091810	6.13811600	-1.16294800
C	11.3184130	7.48717800	-0.99690400
C	10.0016640	7.87433500	-0.79096500
C	8.99941700	6.89471600	-0.77157600
C	9.37353600	5.55769100	-0.97285400
H	8.62899600	4.76162900	-1.01697200
C	7.62713100	7.23296600	-0.55281900
C	6.46541500	7.52407400	-0.35740300
C	5.09414200	7.86163000	-0.12640800
C	4.72002400	9.20049200	0.05129800
C	3.38866300	9.53752100	0.27466100
C	2.41676800	8.54398700	0.32682700
H	1.37190700	8.79697600	0.50681900
H	3.10726900	10.5819600	0.41130200
H	5.48972100	9.97118200	0.01003500
H	9.73567400	8.92172100	-0.64447300
H	12.1268300	8.21665500	-1.02053700
H	12.6303900	5.77854300	-1.28424300
H	9.85708900	0.54277100	-1.80568800
H	9.81931400	-5.58744300	-1.92738400

H	8.08386200	-7.37235700	-1.83225700
H	5.70141300	-6.76152900	-1.43103100
C	4.76085000	0.69462500	-1.35687100
C	4.35568200	2.02711900	-1.24335900
C	3.11246300	2.46074700	-1.79904000
C	2.07133200	2.86898200	-2.27593100
C	0.81963500	3.38418200	-2.74948800
C	-0.29541300	2.53718000	-2.86553200
C	-1.53686800	3.06084000	-3.21170700
C	-1.67515900	4.42697500	-3.46485500
C	-0.56560300	5.26983800	-3.37179100
C	0.67773900	4.75684700	-3.01354100
H	1.54616700	5.40952000	-2.91481900
H	-0.67066500	6.33673300	-3.57498500
H	-2.64911900	4.83654400	-3.73830600
H	-2.39322700	2.38658600	-3.26824000
H	-0.20209200	1.47057100	-2.65361700
H	4.11527500	-0.01519400	-1.87340600
C	8.07898400	0.74884400	0.37865600
C	9.12006700	0.34051500	0.85557500
C	10.3716550	-0.17477900	1.32932000
C	11.4867730	0.67212100	1.44547300
C	12.7281200	0.14835500	1.79188300
C	12.8662360	-1.21777500	2.04514500
C	11.7566120	-2.06053300	1.95194900
C	10.5133770	-1.54744300	1.59347500
H	9.64490300	-2.20003900	1.49465000
H	11.8615380	-3.12742800	2.15521600
H	13.8401070	-1.62742100	2.31879800
H	13.5845430	0.82251900	1.84851700
H	11.3935970	1.73871900	1.23343600
H	7.07618300	3.22480600	0.45301300
C	-1.46500700	4.60964100	0.30262400
C	-2.38563400	3.56973400	0.31471500
C	-1.92645400	2.25915600	0.30078200
H	-2.60958900	1.41305200	0.24521300
H	-3.45772200	3.76141700	0.30651100
H	-1.78611400	5.65140700	0.27170400
H	1.33422400	2.66674200	0.38515000

$4\text{-}I^+$ $\varphi_1 = 37.5°$

E = -3345.783239 Hartrees

G = -3345.085559 Hartrees

E(TZVPP)= -3348.799331 Hartrees

N	0.00000000	0.00000000	0.00000000

C	0.83846400	1.01939700	-0.19158900
C	0.40269200	2.34651800	-0.09133500
C	-0.95037300	2.57667600	0.20131900
C	-1.80665000	1.49493400	0.37529400
C	-1.29535500	0.20859100	0.27666100
H	-1.91729600	-0.67625600	0.42455300
H	-2.86224100	1.64033400	0.60236700
H	-1.31419600	3.60027500	0.29649500
C	1.33943600	3.41544700	-0.22609000
C	2.13725000	4.32782400	-0.28808100
C	3.04421600	5.43393900	-0.32546600
C	4.41987500	5.24353100	-0.05195000
C	5.27297600	6.37310800	-0.04773100
C	4.75989700	7.64507500	-0.33886900
C	3.40942000	7.81325800	-0.62669400
C	2.55445000	6.71661300	-0.61291300
H	1.49262800	6.83943900	-0.82634200
H	3.02018900	8.80544600	-0.85514000
H	5.43532900	8.50054500	-0.33216300
C	6.65755800	6.23432300	0.28550000
C	7.82717400	6.15112200	0.59758400
C	9.19783600	6.02783300	0.97849900
C	9.75919900	4.75536100	1.14119900
N	11.0347630	4.59271700	1.49349100
C	11.8288550	5.64844100	1.71458500
C	11.3486240	6.94581800	1.57888000
C	10.0240150	7.14142600	1.20471500
H	9.61862800	8.14686600	1.08656600
H	12.0129080	7.78935600	1.76434000
H	12.8612070	5.43941800	2.00179100
I	11.8675150	2.46489200	1.63698400
N	12.6834750	0.36381700	1.69876200
C	13.9788770	0.15526700	1.42226300
C	14.4902120	-1.13105800	1.32365000
C	13.6339340	-2.21282800	1.49747300
C	12.2808360	-1.98271500	1.78999100
C	11.8450180	-0.65560500	1.89022300
H	10.7994260	-0.42242200	2.10050000
C	11.3441120	-3.05167700	1.92462600
C	10.5462810	-3.96404300	1.98655300
C	9.63928200	-5.07012900	2.02402900
C	8.26361900	-4.87968400	1.75054900
C	7.75127400	-3.57113600	1.48563200
C	7.32735600	-2.44973600	1.28542300
C	6.83356000	-1.12511800	1.05929600
C	7.60632000	-0.18192300	0.32920200

C	7.09802700	1.10770500	0.13400400
C	5.85003300	1.48905600	0.63952200
C	5.35617300	2.81363700	0.41335800
C	4.93223600	3.93501400	0.21305600
C	5.07727700	0.54586200	1.36959200
C	5.58555500	-0.74376700	1.56478500
H	5.00477200	-1.46104200	2.14490100
C	3.80730700	0.91933300	1.91049400
C	2.74566700	1.27619000	2.38468300
C	1.50003000	1.74420100	2.91970300
C	0.47927200	0.84095300	3.26351700
C	-0.73590600	1.31530100	3.74973900
C	-0.94630600	2.68787600	3.89388500
C	0.06648100	3.58902600	3.55861900
C	1.28644700	3.12473500	3.07654300
H	2.08534400	3.81931900	2.81395100
H	-0.09235700	4.66067700	3.68618800
H	-1.89644000	3.05587400	4.28382300
H	-1.52088700	0.61062700	4.02873800
H	0.65451200	-0.23134200	3.15564900
H	7.67880200	1.82498100	-0.44611600
C	8.87633000	-0.55540200	-0.21160200
C	9.93803300	-0.91207800	-0.68578700
C	11.1835910	-1.38012800	-1.22095600
C	11.3970830	-2.76067100	-1.37784400
C	12.6169740	-3.22502600	-1.86005400
C	13.6297730	-2.32392700	-2.19541000
C	13.4194610	-0.95134000	-2.05122200
C	12.2043670	-0.47693400	-1.56485300
H	12.0291940	0.59537100	-1.45697200
H	14.2044490	-0.24670400	-2.33029600
H	14.5798500	-2.69196900	-2.58544500
H	12.7757390	-4.29668300	-1.98765500
H	10.5981700	-3.45521500	-1.11519400
C	7.41048900	-6.00923300	1.74633800
C	7.92355600	-7.28122800	2.03739000
C	9.27403300	-7.44944900	2.32518600
C	10.1290260	-6.35281900	2.31144000
H	11.1908380	-6.47565900	2.52491600
H	9.66324400	-8.44165300	2.55359500
H	7.24812500	-8.13669800	2.03057700
C	6.02590100	-5.87040800	1.41313800
C	4.85630000	-5.78725600	1.10098400
C	3.48572900	-5.66398200	0.71973400
C	2.92424400	-4.39152600	0.55735800
H	3.52188100	-3.49158400	0.71741300

N 1.64875600 -4.22891900 0.20475200
C 0.85486900 -5.28466200 -0.01695300
C 1.33523100 -6.58202900 0.11840600
C 2.65976000 -6.77760100 0.49286100
H 3.06525500 -7.78303100 0.61073200
H 0.67110600 -7.42558400 -0.06754900
H -0.17743500 -5.07566600 -0.30435700
I 0.81592200 -2.10109300 0.06160800
H 13.9977960 -3.23641400 1.40230100
H 15.5458350 -1.27642800 1.09671000
H 14.6008160 1.04013300 1.27447900
H 9.16140500 3.85543600 0.98162900
H 1.88402900 0.78618100 -0.40196200

4-I$^+$ $\varphi_1 = 143.5°$
E = -3345.783239 Hartrees
G = -3345.085559 Hartrees
E(TZVPP)= -3348.799331 Hartrees
N 0.00000000 0.00000000 0.00000000
C -1.27551200 0.16249800 0.35256600
C -1.83707000 1.43490900 0.51507600
C -1.01114700 2.54859800 0.28838200
C 0.31340600 2.35313900 -0.08604900
C 0.79384000 1.05581500 -0.22155200
H 1.82616100 0.84690900 -0.50895400
H 0.97749100 3.19675200 -0.27188400
H -1.41669600 3.55399300 0.40635500
C -3.20767500 1.55805300 0.89624800
C -4.37723900 1.64136700 1.20849700
C -5.76179500 1.78038200 1.54174700
C -6.61507400 0.65094300 1.54596900
C -7.99069500 0.84155400 1.81949200
C -8.48027100 2.12430700 2.10691600
C -7.62513500 3.22082700 2.12067600
C -6.27468200 3.05243600 1.83285900
H -5.59912400 3.90780600 1.82612200
H -8.01421900 4.21307800 2.34909600
H -9.54207600 2.24729600 2.32034000
C -8.89783200 -0.26441600 1.78203200
C -9.69563000 -1.17680600 1.72001700
C -10.6323370 -2.24580200 1.58552900
C -10.1965010 -3.57289700 1.68591200
N -11.0349630 -4.59235600 1.49463500
C -12.3303730 -4.38385400 1.21815700
C -12.8417200 -3.09754500 1.11937700
C -11.9854440 -2.01574300 1.29300500

H	-12.3493210	-0.99217300	1.19771800
H	-13.8973560	-2.95221700	0.89246500
H	-12.9523090	-5.26874600	1.07051300
I	-10.2189900	-6.69343400	1.43307700
N	-9.38613300	-8.82124800	1.28986500
C	-10.1801240	-9.87697800	1.51129700
C	-9.69983300	-11.1743530	1.37578300
C	-8.37527400	-11.3699540	1.00143900
C	-7.54920100	-10.2563550	0.77486000
C	-8.11061600	-8.98388500	0.93739800
H	-7.51290900	-8.08395600	0.77752900
C	-6.17860000	-10.3796370	0.39372800
C	-5.00904500	-10.4629680	0.08144700
C	-3.62451500	-10.6018340	-0.25196100
C	-2.77136500	-9.47229700	-0.25631000
C	-3.28363200	-8.16374400	0.00869200
C	-3.70752000	-7.04234400	0.20895700
C	-4.20147100	-5.71778800	0.43505800
C	-3.42873200	-4.77445000	1.16497000
C	-3.93713000	-3.48486100	1.36010500
C	-5.18522600	-3.10367900	0.85471200
C	-5.67912500	-1.77908900	1.08071500
C	-6.10292300	-0.65765100	1.28095700
C	-5.95798700	-4.04702200	0.12483100
C	-5.44956500	-5.33660400	-0.07035000
H	-6.03032300	-6.05398100	-0.65036100
C	-7.22806000	-3.67369400	-0.41593400
C	-8.28975800	-3.31701000	-0.89012700
C	-9.53527000	-2.84881800	-1.42528100
C	-10.5561510	-3.75189900	-1.76916300
C	-11.7712170	-3.27735900	-2.25547800
C	-11.9814030	-1.90474700	-2.39959800
C	-10.9684930	-1.00376200	-2.06426900
C	-9.74862000	-1.46825200	-1.58215000
H	-8.94961900	-0.77380000	-1.31953000
H	-11.1271510	0.06791400	-2.19184800
H	-12.9314570	-1.53659600	-2.78958700
H	-12.5562820	-3.98190800	-2.53455500
H	-10.3810690	-4.82422500	-1.66134700
H	-3.35634300	-2.76746500	1.94006600
C	-2.15864300	-5.14772600	1.70572200
C	-1.09688800	-5.50426500	2.17989100
C	0.14872500	-5.97224900	2.71498400
C	0.36224900	-7.35277100	2.87202800
C	1.58219300	-7.81704500	3.35418100
C	2.59500800	-6.91588900	3.68933900

C	2.38466300	-5.54332600	3.54500000
C	1.16952000	-5.06899700	3.05867100
H	0.99432200	-3.99670900	2.95067000
H	3.16966600	-4.83864400	3.82391700
H	3.54512400	-7.28386800	4.07934200
H	1.74098500	-8.88868400	3.48190800
H	-0.43667800	-8.04735800	2.60953900
C	-1.39574300	-9.66277200	-0.52993900
C	-0.90606000	-10.9454770	-0.81740100
C	-1.76107900	-12.0420870	-0.83105400
C	-3.11151800	-11.8738380	-0.54308800
H	-3.78698100	-12.7292830	-0.53625800
H	-1.37191100	-13.0343010	-1.05949300
H	0.15573600	-11.0683550	-1.03093500
C	-0.48872800	-8.55669600	-0.49260100
C	0.30906700	-7.64429600	-0.43071300
C	1.24588200	-6.57542100	-0.29601900
C	0.81021800	-5.24826800	-0.39635000
H	-0.23530700	-5.01497600	-0.60683200
N	1.64875600	-4.22893000	-0.20476100
C	2.94408600	-4.43761600	0.07195800
C	3.45527200	-5.72399400	0.17066800
C	2.59891200	-6.80567500	-0.00328300
H	2.96264900	-7.82929700	0.09196800
H	4.51084200	-5.86947200	0.39779000
H	3.56609600	-3.55281200	0.21981600
I	0.83304200	-2.12774700	-0.14330400
H	-7.96984500	-12.3753940	0.88342200
H	-10.3640370	-12.0178950	1.56151500
H	-11.2124490	-9.66796100	1.79860600
H	-9.15089700	-3.80604300	1.89616900
H	-1.87311400	-0.73749300	0.51247900

Computational Methods

Calculations were performed with M06-2X/def2-SVP using the Gaussian16 suite of electronic structure program and visualized with WebMO. Calculations were performed using the ultrafine grid (int=ultrafine) and tight SCF convergence criteria (SCF=tight). Structures for **1-**, **2-**, **3-**, and **4-Ag$^+$**, **$PdCl_2$**, and **I^+** were optimized and confirmed minima by vibrational frequency calculations. Transition states were optimized and confirmed by vibrational frequency calculations for the **1-** systems. To test basis set dependence, single point energy calculations for minima and transition states were performed with the def2-TZVPP basis set. Minima and transitions states were also optimized using ωB97x-d/def2-SVP. Relative energies in kcal/mol are listed in the tables below.

Table S1. Comparison of relative energies (kcal/mol) of 1-Ag$^+$ structures for basis sets and density functional.

	**M06-2X**		
	**def2-SVP**	**def2-TZVPP**	**wb97x-d/def2-SVP**
1-Ag+ 65	0	0.0	0.0
1-Ag+ 123	0	0.0	0.0
TS 1-Ag+	5.2	5.2	3.9
Internal TS 1-Ag+	0.2	0.1	0.1

Table S2. Comparison of relative energies (kcal/mol) of 2-Ag$^+$ structures for basis sets and density functional.

	**M06-2X**		
	**def2-SVP**	**def2-TZVPP**	**wb97x-d/def2-SVP**
2-Ag+ both out of plane	0	0.0	0.0
2-Ag+ one in/one out	0.6	0.1	0.4
2-Ag+ both in plane	1.3	0.4	0.9

Table S3. Comparison of relative energies (kcal/mol) of 1-$PdCl_2$ structures for basis sets and density functional.

M06-2X

	def2-SVP	def2-TZVPP	wb97x-d/def2-SVP
1-PdCl2 arene perp	0.0	0.0	0.0
1-PdCl2 all parallel	5.2	4.4	4.3
1-PdCl2 Cl Out of Plane	2.7	2.2	2.2
TS 1-PdCl2	5.5	5.1	5.1

Table S4. Comparison of relative energies (kcal/mol) of 2-, 3-, and 4-$PdCl_2$ structures for basis sets and density functional.

	M06-2X		
	def2-SVP	def2-TZVPP	wb97x-d/def2-SVP
2-PdCl2 all parallel	0	0.0	0.0
2-PdCl2 arene perpendicular	9.5	6.8	10.9
3-PdCl2 all in plane	0.0	0.0	0.0
3-PdCl2 are perpendicular	5.4	3.4	5.8
4-PdCl2 all parallel	0	0.0	0.0
4-PdCl2 arene perpendicular	3.5	3.6	4.0

Table S5. Comparison of relative energies (kcal/mol) of 1-I^+ structures for basis sets and density functional.

	M06-2X		
	def2-SVP	def2-TZVPP	wb97x-d/def2-SVP
1-I + 52.5	0	0.0	0.0
1-I+ 122.2	0	0.0	0.0
TS 1-I+	4.2	4.4	2.8
Internal TS 1-I+	0.2	0.1	0.3

Table S6. Comparison of relative energies (kcal/mol) of 2-Ag^+ structures for basis sets and density functional.

	**M06-2X**		
	**def2-SVP**	**def2-TZVPP**	**wb97x-d/def2-SVP**
2-I+ both out of plane	0.5	0.2	0.0
2-I+ one in/one out	0.6	0.2	0.3
2-I+ both in plane	0.0	0.0	0.2
